# 1,3-Diphenylureido hydroxamate as a promising scaffold for generation of potent antimalarial histone deacetylase inhibitors

**DOI:** 10.1038/s41598-023-47959-z

**Published:** 2023-11-29

**Authors:** Maurício T. Tavares, Arne Krüger, Sun L. Rei Yan, Karoline B. Waitman, Vinícius M. Gomes, Daffiny Sumam de Oliveira, Franciarli Paz, Sebastian Hilscher, Mike Schutkowski, Wolfgang Sippl, Claudia Ruiz, Mônica F. Z. J. Toledo, Neuza M. A. Hassimotto, João A. Machado-Neto, Antti Poso, Michael D. Cameron, Thomas D. Bannister, Giuseppe Palmisano, Carsten Wrenger, Thales Kronenberger, Roberto Parise-Filho

**Affiliations:** 1 Department of Pharmacy, Faculty of Pharmaceutical Sciences, University of São Paulo, Av. Prof. Lineu Prestes 580, São Paulo, 05508-000 Brazil; 2 Department of Molecular Medicine, The Herbert Wertheim Institute for Biomedical Innovation and Technology, Jupiter, FL 33458 USA; 3 Department of Cancer Biology, Dana-Farber Cancer Institute, Boston, MA 02115 USA; 4 Department of Biological Chemistry and Molecular Pharmacology, Harvard Medical School, Boston, MA 02115 USA; 5 Unit for Drug Discovery, Department of Parasitology, Institute of Biomedical Sciences, University of São Paulo, Av. Prof. Lineu Prestes 1374, São Paulo, 05508-900 Brazil; 6 GlycoProteomics Laboratory, Department of Parasitology, Institute of Biomedical Sciences, University of Sao Paulo, São Paulo, Brazil; 7 School of Natural Sciences, Faculty of Science and Engineering, Macquarie University, Sydney, Australia; 8 Faculty of Biosciences, Martin-Luther-University of Halle-Wittenberg, 06120 Halle/Saale, Germany; 9 Food Research Center-(FoRC-CEPID) and Department of Food Science and Nutrition, Faculty of Pharmaceutical Science, University of São Paulo, São Paulo, SP Brazil; 10 Department of Pharmacology, Institute of Biomedical Sciences, University of São Paulo, São Paulo, Brazil; 11 Department of Pharmaceutical and Medicinal Chemistry, Institute of Pharmaceutical Sciences, Eberhard-Karls-Universität, Tuebingen, Auf der Morgenstelle 8, 72076 Tübingen, Germany; 12 Tuebingen Center for Academic Drug Discovery & Development (TüCAD2), 72076 Tübingen, Germany; 13 School of Pharmacy, Faculty of Health Sciences, University of Eastern Finland, P.O. Box 1627, 70211 Kuopio, Finland

**Keywords:** Screening, Computational chemistry, Antiparasitic agents, Pharmacokinetics

## Abstract

We report a series of 1,3-diphenylureido hydroxamate HDAC inhibitors evaluated against sensitive and drug-resistant *P. falciparum* strains. Compounds **8a–d** show potent antiplasmodial activity, indicating that a phenyl spacer allows improved potency relative to cinnamyl and di-hydrocinnamyl linkers. In vitro*,* mechanistic studies demonstrated target activity for *Pf*HDAC1 on a recombinant level, which agreed with cell quantification of the acetylated histone levels. Compounds **6c**, **7c**, and **8c**, identified as the most active in phenotypic assays and *Pf*HDAC1 enzymatic inhibition. Compound **8c** stands out as a remarkable inhibitor, displaying an impressive 85% inhibition of *Pf*HDAC1, with an IC_50_ value of 0.74 µM in the phenotypic screening on *Pf*3D7 and 0.8 µM against multidrug-resistant *Pf*Dd2 parasites. Despite its potent inhibition of *Pf*HDAC1, **8c** remains the least active on human HDAC1, displaying remarkable selectivity. In silico studies suggest that the phenyl linker has an ideal length in the series for permitting effective interactions of the hydroxamate with *Pf*HDAC1 and that this compound series could bind as well as in *Hs*HDAC1. Taken together, these results highlight the potential of diphenylurea hydroxamates as a privileged scaffold for the generation of potent antimalarial HDAC inhibitors with improved selectivity over human HDACs.

## Introduction

Infectious diseases are among the leading causes of death globally, collectively second only to cardiovascular diseases. Malaria, a parasitic infection caused by *Plasmodium* spp., has been among mankind’s deadliest diseases, affecting over 247 million people in 2021 in endemic countries and causing 619,000 deaths worldwide^1^. Despite large investments to develop alternative pharmacological interventions such as malaria vaccines, effective malaria control likely relies upon the continued development of new small-molecule antimalarial drugs^2^. To date, the antimalarial agents can be grouped into seven main classes: arylaminoalcohols (quinine derivatives), 4- and 8-aminoquinolines (chloroquine and primaquine derivatives, respectively), analogues of artemisinin, antifolates (*e.g.* pyrimethamine), antibiotics (*e.g.* tetracycline), and other agents (*e.g.* atovaquone)^3^. A major limitation of current antimalarial chemotherapy is the rapid spread of drug-resistant *Plasmodium falciparum* parasites following first-line treatments (artemisinin-based combination therapies) or following alternative drug combination protocols^4–7^. A strategy to combat malaria drug resistance is to identify and develop new antimalarial drugs acting on novel parasite targets, likely to be used to complement the existing treatment options.

The transcriptional control in malaria parasites is complex and has been the subject of extensive investigation. Moreover, there is increasing evidence that targeting transcriptional regulation represents a potential new therapeutic approach for malaria^8^. In this sense, histone deacetylase enzymes (HDACs) are well-known key regulators of transcription and human HDACs are validated targets for some types of hematological cancers^9^. In eukaryotes, a homeostatic balance of the acetylation state of histones is modulated by the coordinated activity of histone acetyltransferases (HATs) and HDACs^10^. HATs increase the acetylation of certain amino acid residues on histone proteins, resulting in a less condensed segment of DNA that can be transcribed. On the other hand, HDACs regulate target genes through the deacetylation of key lysine residues in histones and non-histone substrates, thus promoting DNA condensation and suppression of gene expression^11^. This epigenetic regulation is observed in all stages of the *Plasmodium* life cycle, is critical to parasite stress response, and is thought to contribute to the transcriptional regulation of drug resistance^12,13^. Human HDACs can be classified into three classes of zinc-dependent enzymes and one NAD^+^-dependent class also called sirtuins^14^. To date, three classes of HDACs have been identified in *P. falciparum*: (1) *Pf*HDAC1 (PFI1260c) is a predominant nuclear class I HDAC enzyme; (2) *Pf*HDAC2 (PF14_0690) and *Pf*HDAC3 (PF10_0078) are assigned to class II HDACs; (3) and two sirtuins *Pf*Sir2A (PF13_0152) and *Pf*Sir2B (PF14_0489)^15^. Noteworthy, *Pf*Sir2A and 2B are recognized as nonessential enzymes and are primarily involved in the regulation of *var* gene expression, involved with antigenic variation making them less appealing as drug targets^16^. On the other hand, *Pf*HDAC1 proved to be the highly conserved isozyme among all species and is involved in gametocytogenesis, schizogony, and hepatocyte invasion besides exhibiting moderate similarity (~ 61%) to human HDACs (*h*HDACs)^17–19^.

**Figure 1:**
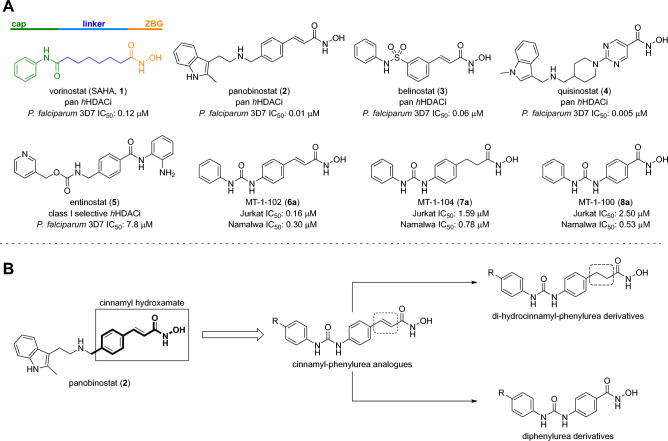
(**A**) Structures of some FDA-approved HDACis that present potent antiplasmodial activity and compounds **6a–8a**, analogues of panobinostat. (**B**) Design of our urea-derived analogues of panobinostat. ZBG: zinc-binding group.

Recent studies have highlighted the potential of HDAC inhibitors (HDACis) as antiplasmodial agents. Moreover, FDA-approved drugs targeting human HDACs, such as vorinostat (SAHA, **1**), panobinostat (**2**), belinostat (**3**), and quisinostat (**4**), have been repurposed for malaria treatment, exhibiting submicromolar to low nanomolar potency over *P. falciparum* 3D7 parasites (Fig. 1A)^20–23^. However, selectivity towards the plasmodial HDAC *versus* human HDACs is desired in malarial treatment for avoiding potential off-target effects.

A typical HDACi has a zinc-binding group (ZBG) that coordinates with the active zinc (Zn^2+^) ion at the catalytic cavity; a capping group (cap) that makes distal and mostly hydrophobic interactions with the target; and a linker of appropriate size to connect the cap and ZBG (Fig. 1A). The ZBG most commonly used is a hydroxamic acid, likely due to its bivalent high affinity coordination of Zn^2+^. The strength of this interaction confers high potency but often limits HDACi utility due to low HDAC selectivity, giving a pan-HDACi profile targeting multiple classes of HDACs and eliciting unwanted side effects. Selectivity concerns must be weighed against potency evaluations, where HDACis have proven to be highly active against laboratory strains and clinical isolates of both *P. falciparum* and *P. vivax*, besides demonstrating the ability to prevent schizont maturation in *P. vivax*^24,25^. Non-hydroxamate ZBGs, such as the *N*-(2-aminophenyl)benzamide moiety observed in entinostat (**5**, Fig. 1A), generally give lower potency (~ 65-fold reduction for **5** *vs.* SAHA (**1**))^26,27^. This suggests that hydroxamic acids can be a privileged scaffold for the generation of potent antiplasmodial HDACis, with the caveat that selectivity issues must be overcome by maximizing other interactions with the target protein^28^.

Recently, our groups identified a series of cinnamyl and phenyl urea-derived analogues of **2**, that showed potent cytotoxic activity against hematological tumor cells^29^. Given the structural similarity of **2** and our cytotoxic hit compounds **6a–8a** (Fig. 1A), we considered the design of additional urea-containing HDACis and their evaluation as new antiplasmodial agents against drug-sensitive and multidrug-resistant *P. falciparum* strains (*Pf*3D7 and *Pf*Dd2, respectively), as well as against *Pf*HDAC1 and a selective panel of representative human HDACs. Nineteen structural variations were explored, and we herein provide evidence that optimal compounds in the series promote an increase of acetylated-H3 and -H4 on a cellular level, validating relevant HDAC inhibition in a cellular context. Subsequent structure–activity relationship (SAR) and in silico studies show that *Pf*HDAC and human HDAC1 (*Hs*HDAC1 and 6) share common structural binding features, which prompt us to suggest that our compounds bind to the *Plasmodium*’s HDAC1 homologue. Preliminary drug metabolism and pharmacokinetics (DMPK) studies indicated that compounds **6c**, **7c**, and **8a–d** were stable in human microsomes and did not significantly inhibit several human CYP450 enzymes responsible for first-pass drug metabolism in vivo.

## Results

**Figure 2:**
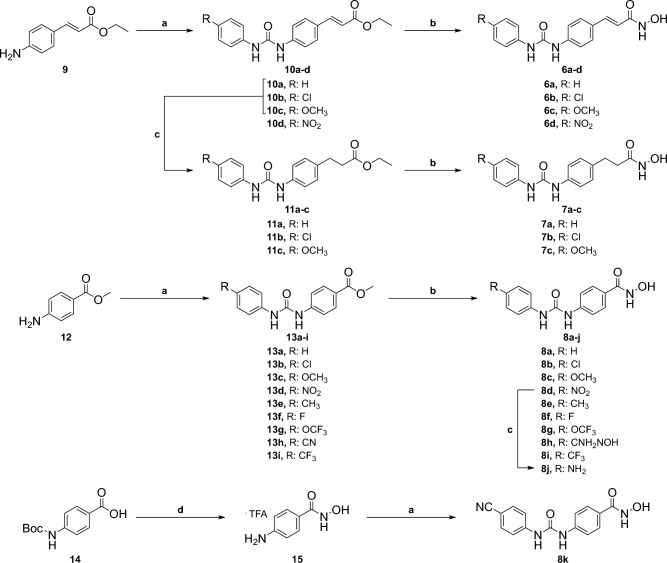
Design and synthesis of analogues **6a–d**, **7a–c**, and **8a–k**. Reagents and conditions: (**a**) appropriate phenyl isocyanate, DCM, r.t., 16 h; (**b**) NH_2_OH (50 wt % in H_2_O), NaOH, THF/MeOH (1:1), 0 °C—r.t., 2 h; (**c**) 10% Pd/C, H_2_, EtOH, r.t., 18 h. (**d**) i. *O*-(*tert*-butyldimethylsilyl)hydroxylamine, HATU, DIPEA, DCM, r.t., 16 h; ii. TFA, DCM, r.t., 3 h.

**Design and synthesis of the urea-derived HDAC inhibitors** The original concept that supported the synthesis of the series considered the cinnamyl hydroxamate of **2** as the primary linker-ZBG moiety (Fig. 1B) which was attached to four different *para*-phenylureas (compounds **6a–d**, Fig. 2). Subsequently, the cinnamyl double bond was hydrogenated (compounds **7a–c**, Fig. 2) to evaluate the influence of a saturated linker over the biological activity (Fig. 1B). Moreover, the double bond was removed from the original scaffold (Fig. 1B), generating the preliminary phenyl-hydroxamates **8a–d** (Fig. 2). After our first round of antiplasmodial screening, we synthesized additional compounds **8e–k** and **15** to generate SAR insights with respect to the optimization of the capping moiety and the identification of ways to increase productive interactions in the target binding pocket (Fig. 2).

The synthesis started with the addition of ethyl (*E*)-3-(4-aminophenyl)acrylate (**9**) to different phenyl isocyanates in dichloromethane (DCM), generating intermediates **10a–d**. Intermediates **11a–c** have been prepared through catalytic hydrogenation of **10a–c** with palladium on activated charcoal in ethanol as solvent. The final products **6a–d** and **7a–c** were obtained by reaction of intermediates **10a–d** and **11a–c** with aqueous hydroxylamine under basic conditions. The synthesis of compounds **8a–i** started with the addition of methyl 4-aminobenzoate (**12**) to appropriate phenyl isocyanates generating intermediates **13a–i**. The final compounds **8a–i** were prepared under same conditions as described above for **6a–d** and **7a–c**. The amino compound **8j** was obtained through standard catalytic hydrogenation of **8d** with palladium. Compound **8k** has been prepared by coupling 4-(boc-amino)benzoic acid (**14**) and *O*-(*tert*-butyldimethylsilyl)hydroxylamine under HATU conditions. Deprotection with trifluoroacetic acid (TFA) provided **15**, which was converted into **8 k** after addition to 4-cyanophenyl isocyanate in DCM^23^.

**Antiplasmodial activity and effects on cell viability of compounds 6a–d, 7a–c, 8a–k** A preliminary screen for compounds **6a–d**, **7a–c**, and **8a–d** against *P. falciparum* 3D7 in three different concentrations (200, 20, and 2 μM) was used to investigate their antiplasmodial potential (Fig. S1). Subsequently, compounds have been submitted to dose–response assays to determine their IC_50_ values (Table 1). To evaluate the therapeutic window as well as the selectivity index (SI) of the compounds, the cytotoxic effect against human hepatocarcinoma cells (HepG2) has been also determined (Supporting information, Figs. S1, S2).

**Table 1:** Antiplasmodial activity and cytotoxicity of compounds **6a–d**, **7a–c**, **8a–k**, and **15**.

Compound			*Pf*HDAC1 % Inhibition @ 1.0/10 µM	IC_50_ (µM)		SI^a^
	R_1_	R_2_		*Pf*3D7	HepG2	
6a	H		n.d.^b^	1.72 (1.42–2.03)	22.9	13
6b	Cl		n.d.	7.88 (6.79–9.11)	28.8	3
6c	OCH_3_		69/82	1.03 (0.80–1.25)	13.3	12
6d	NO_2_		n.d.	1.26 (0.69–1.73)	10.4	8
7a	H		n.d.	4.57 (4.02–5.10)	> 50	> 10
7b	Cl		n.d.	7.04 (0.02–15.35)	> 50	> 7
7c	OCH_3_		50/88	5.82 (4.77–7.24)	> 50	> 8
8a	H		n.d.	0.56 (0.36–0.79)^c^	56.0	100
8b	Cl		n.d.	1.31 (0.93–1.93)	> 200	> 153
8c	OCH_3_		38/85	0.74 (0.49–1.06)	> 200	> 271
8d	NO_2_		n.d.	0.55 (0.34–0.78)	54.0	100
8e	CH_3_		n.d.	0.34 (0.19–0.56)	> 200	> 588
8f.	F		n.d.	0.40 (0.32–0.49)	115.7	289
8 g	OCF_3_		n.d.	13.9 (11.52–17.01)	60.5	4
8 h			n.d.	0.39 (0.31–0.48)	> 200	> 512
8i	CF_3_		n.d.	1.30 (1.1–1.64)	25.1	19
8j	NH_2_		n.d.	0.35 (0.23–0.47)	> 200	> 570
8 k	CN		n.d.	0.26 (0.22–0.29)	> 200	> 769
15			n.d.	6.30 (5.24–7.41)	> 200	31
Nexturastat A (16)			58/92	0.23 (0.19–0.29)	21.5	93
Vorinostat (SAHA, 1)			83/n.d.	0.36 (0.26–0.51)	2.87	8
Chloroquine	n.a.^d^		n.a.	0.015	> 100	> 5000

^a^SI: selectivity index, the ratio between *Pf*3D7 IC_50_/HepG2 IC_50_.

^b^n.d.: not determined.

^c^Values in brackets indicate 95% confidence intervals.

^d^n.a.: not applicable.

The main finding of this series is that the use of a diphenylurea cap-linker moiety correlates with higher antiplasmodial potency relative to both cinnamyl and di-hydrocinnamyl cap linkers. Avoiding the use of a cinnamyl hydroxamates gave a benefit with respect to target selectivity, as reflected by mild (IC_50_ > 50 µM) or no measurable cytotoxicity on HepG2 cells (IC_50_ > 200 µM), which differed from the more toxic cinnamyl hydroxamates, corroborating previous findings of the cinnamyl series on HS-5 human hematological cells^26^.

**Figure 3:**
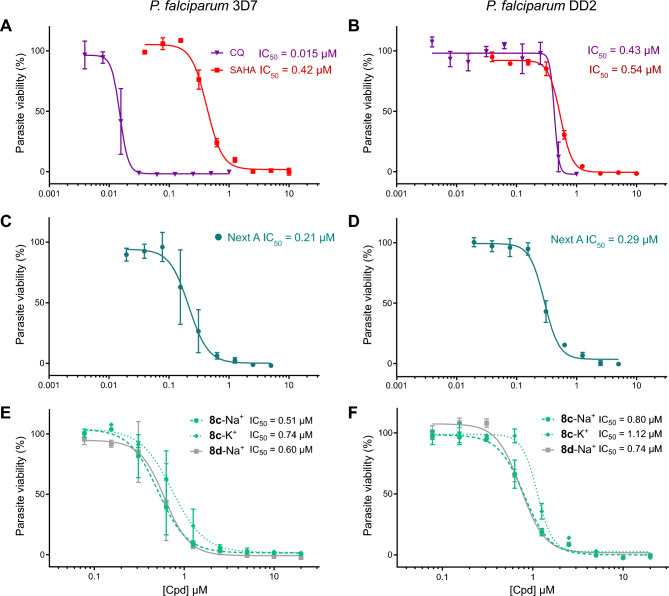
HDAC inhibitors remain effective against CQ-resistant *Plasmodium falciparum* strain Dd2. Antiplasmodial activity against *P. falciparum* 3D7 (**A**, **C** and **E**) and *P. falciparum* Dd2 (**B**, **D**, **F**). Compounds were tested as DMSO stocks in a two-fold serial dilution from 20 µM to 78 nM and proliferation of the parasite was assessed by measuring dsDNA using the SYBR Green I assay (Smilkstein et al. 2004 with modifications)^30^. Relative parasite proliferation was calculated by normalizing measured fluorescence of compounds-treated wells against the chloroquine-treated control and subtracting the background from both. Curves were plotted using non-linear regression based on means from three independent experiments measured in triplicate. Error bars indicate standard deviation, whose values are provided in the respective table. For curves that do not reach the zero-point IC_50_ was not calculated and is indicated as an approximation.

Because the emergence of drug resistance is an important consideration, we assessed the potency of our best compounds against the *Plasmodium falciparum* Dd2 strain (herein named Dd2). While *Pf*Dd2 was ~ 28 × less susceptible to chloroquine (CQ, Fig. 3A, B), SAHA (**1**), and nexturastat A (NextA, **16**, a selective human HDAC6 inhibitor), we observed mostly retained efficacy against this strain with our best compounds (Fig. 3A–D). In particular, the HDACi **8c**, our best non-toxic compound in terms of antiparasitic effects (Fig. 3E, F), and a close analogue (**8d**) show minimal loss in effectiveness against *Pf*Dd2 (~ 1.6 × drop in potency). Interestingly, both NextA (**16**) and SAHA (**1**) have some degree of toxicity *vs.* HepG2 cells (IC_50_ = 21.5 and 96.8 µM, respectively, see Supporting Information, Fig. S4), which is not observed for **8c** (IC_50_ > 200 µM), suggesting that **8c** has the potential to be well-tolerated at therapeutic doses.

### Molecular modelling

The potential binding mode for representative compounds of each synthesized scaffold (**6c**, **7c**, and **8c**) to *Pf*HDAC1, *Hs*HDAC1 and *Hs*HDAC6 was studied using molecular modelling. Briefly, we generated a homology model of *Pf*HDAC1 and *Hs*HDAC6 (based on the PDB ID: 6DVO), while the *Hs*HDAC1 structure was retrieved (PDB 5ICN), which was used for the docking of compounds. Additionally, for *Pf*HDAC1 and HDAC6, we generated mono and bidentate models for the methoxy-substituted compounds, in order to compare their influence on the binding mode, while HDAC1 was exclusively simulated as monodentate. Those restrictions were imposed in order to isolate the effects rising from the different cap changes. Those binding mode models underwent classical molecular dynamics simulation. The predicted binding energy and frequency of interactions along the trajectory were used as parameters for the binding discussion.

The model suggests that the overall binding mode of our compounds within *Pf*HDAC1 is similar to the previously suggested for *Hs*HDAC1 and 6 (Supporting Information, Fig. S5 and Table S1), in several aspects but not the linker. This is supported by the high similarity between the amino acids composing each pocket (Supporting Information, Fig. S6).

From the ZBG perspective, the *Hs*HDAC6 structures usually display monodentate Zn^2+^ coordination mode for sterically bulky HDAC6-selective phenyl hydroxamate inhibitors, while flexible saturated acyl groups retain bidentate coordination^31^. This monodentate binding mode is energetically accessible (0.5 kcal/mol difference between mono and bi)^31^ and common for bulky inhibitors that cannot bind deeper in the HDAC6 narrow pocket, a requirement for effective bidentate coordination. In our simulations, we analyzed both mono and bidenticity and opted to discuss the later as it generated stabler binding conformations (Table S2) and better agreed with the *Plasmodium* counterparts.

Further, the linker interactions with Ser90/Asp97 (HsHDAC6/PfHDAC1, Table 2 and Supporting information, Table S1), seem to be more frequent than previously observed in *Hs*HDAC1^29^, are exemplified in the model. We suggest that this interaction, together with tighter hydrophobic contacts, contributes to the overall lower binding energy in *Pf*HDAC1 when compared to the human counterpart (Supporting information, Table S2). A more comprehensive study of the interactions performed by the cap fragments suggests that stability relies on hydrophobic and pi-mediated contacts. Among the hydrophobic contacts contributed to stabilizing the interaction of our compounds with either *Hs*HDAC1 or *Pf*HDAC1 (such as Phe202/203, Leu269/271 and Tyr301/303, present in all studied isoforms) and towards selectivity against HDAC6. Specifically, interactions between the cap group and the amino acids Pro23 (numbering follows *Hs*HDAC6), Phe142 and Tyr304 seem more frequent than the *Hs*HDAC1 and *Pf*HDAC1 counterparts. HsHDAC6 also engages more frequently in pi-mediated interactions with Phe142 and His173, while *Plasmodium*’s HDAC only uses these residues as polar contacts to the linker.

**Table 2:**
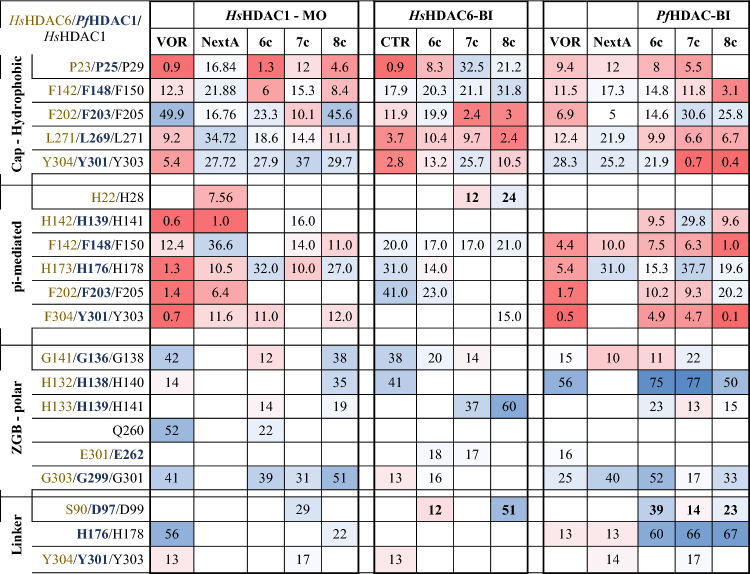
Summary of Protein–ligand interaction frequency during the analyzed trajectory for each compound.

Frequency is displayed as (%) of the hydrogen bond, water-mediated interactions or pi-pi interactions, and separated according to the compound moiety performing it. The full description can be found in Table Table S1.

Comparison between multiple crystal structures showing that cyclic linkers exhibit selectivity versus HDAC6, independently from the saturation state: *i.e.* saturated, partially unsaturated, and aromatic, showing entropy-driven binding^31^. However, HDAC6 specific inhibitor’s cap displays a preference for the shallow portion of the pocket, meaning the two covering loops (L1 and L2). Nonbranched cap inhibitors point toward loop L1, in crystal structures, whereas the branched ligands would interact with both loops L1 and L2, accordingly, our best inhibitors engage more frequently with the L1.

### Target engagement validation via in vitro enzymatic assay and Western Blot analysis

**Figure 4:**
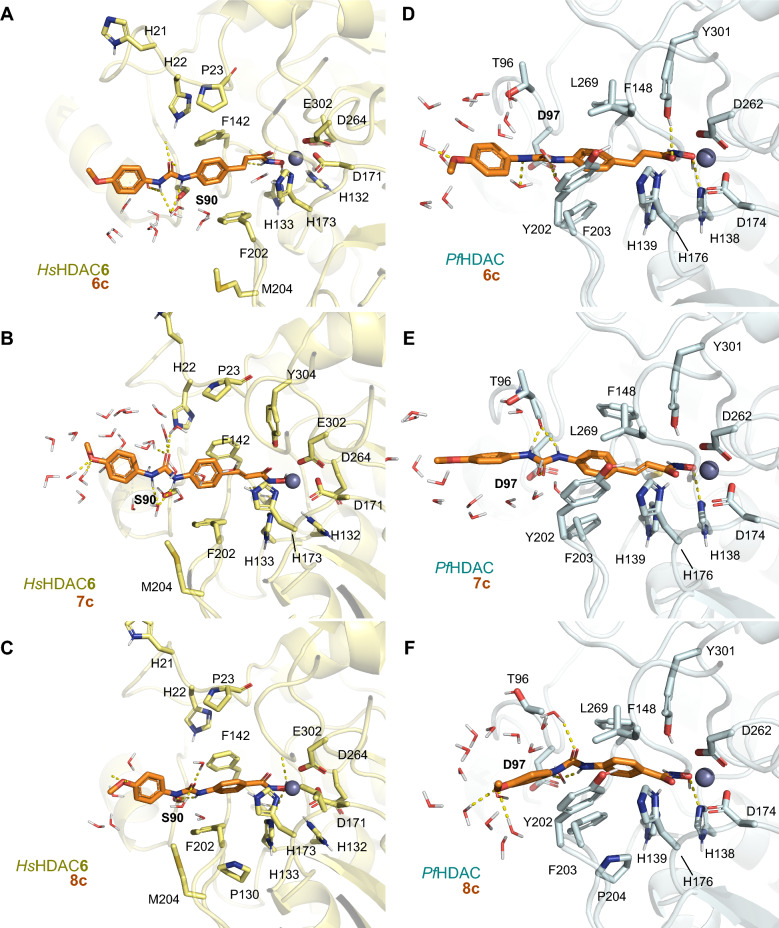
Relevant frames from the MD simulation, display the potential binding mode of our compounds, **6c** (**A, D**), 7c (**B, E**) and **8c** (**C, F**) within the conserved binding site of the *Pf*HDAC1 model.

Our compounds were biochemically evaluated for human HDAC1/8 (Class I), and HDAC6 (Class IIb) inhibition. It is evident that compounds from the cinnamyl series **6a**–**d** (IC_50_ = 9.0–14.3 nM, Table 3) and phenyl series **8a**–**k** (IC_50_ = 8.5–21.3 nM) demonstrated significant activity against the human HDAC6, markedly outperforming their activity against other isoforms. In contrast, the dihydrocinnamyl series **7a**–**c** (IC_50_ = 151.4–184.9 nM) proved to be the least active/selective against HDAC6. It is noteworthy that all three series exhibited a degree of selectivity for this isoform compared to HDAC1/8. Interestingly, all series were less active against HDAC1 (**6a**–**6d**, IC_50_ = 1.1–2.7 μM; **7a**–**7c**, IC_50_ = 3.5–4.8 μM; **8a**–**k**, IC_50_ = 3.7–8.2 μM), indicating a high degree of selectivity, especially against HDAC1, as indicated in Table 3. However, it is relevant to mention that all compounds showed moderate activity against class I HDAC8, with **7a**–**c** being four times less active in this isoform compared to other compounds of the series (Table 3 and respective binding mode are depicted in the Fig. 4).

**Table 3:** Potential *Pf*HDAC inhibitors tested by means of previously reported biochemical in vitro deacetylation assays in human isoforms^32,33^.

Compound			IC_50_ (nM ± SD)			HDAC1/6 SI^a^
	R_1_	R_2_	HDAC1	HDAC6	HDAC8	
6a	H		1352.1 ± 64.2	12.4 ± 0.7	95.1 ± 4.6	109
6b	Cl		2722.7 ± 281.0	14.3 ± 2.2	505.8 ± 62.8	190
6c	**OCH**_**3**_		**1584.9 ± 62.2**	**7.7 ± 0.9**	**63.7 ± 4.2**	**206**
6d	NO_2_		1114.3 ± 56.3	9.0 ± 0.7	99.1 ± 10.9	124
7a	H		4886.5 ± 210.1	152.4 ± 11.6	462.4 ± 27.6	32
7b	Cl		4775.3 ± 446.1	184.9 ± 21.6	567.5 ± 40.5	26
7c	**OCH**_**3**_		**3556.3 ± 184.8**	**151.4 ± 11.1**	**362.2 ± 22.7**	**23**
8a	H		5333.4 ± 366.1	8.5 ± 0.8	93.5 ± 5.3	627
8b	Cl		5236.0 ± 476.4	15.0 ± 0.4	85.1 ± 9.6	349
8c	**OCH**_**3**_		**6180.2 ± 230.9**	**15.9 ± 0.4**	**136.5 ± 8.2**	**389**
8d	NO_2_		3741.1 ± 231.9	13.4 ± 0.5	68.1 ± 6.8	279
8 h	CH_3_		8298.5 ± 481.4	21.3 ± 0.5	164.1 ± 9.1	390
8 k	F		3749.7 ± 562.8	11.6 ± 0.2	51.5 ± 6.1	323
NextA (16)			3176.9 ± 131.3	1.3 ± 0.1	553.4 ± 32.1	2444
Vorinostat (SAHA, 1)			100.1 ± 7.0	27.0^b^	420.0 ± 80.1	4

IC_50_ values [nM, mean ± SD] of the inhibitors, as well as reference compounds for selective HDAC6 inhibition (Nexturastat A, **16**), and Vorinostat (SAHA, **1**) as a non-selective inhibitor of Zn^2+^ -dependent HDACs.

Relevant compound’s values are in [bold].

^a^SI: selectivity index, the ratio between *Hs*HDAC1 IC_50_/*Hs*HDAC6 IC_50_.

^b^Ref.:^34^.

**Figure 5:**
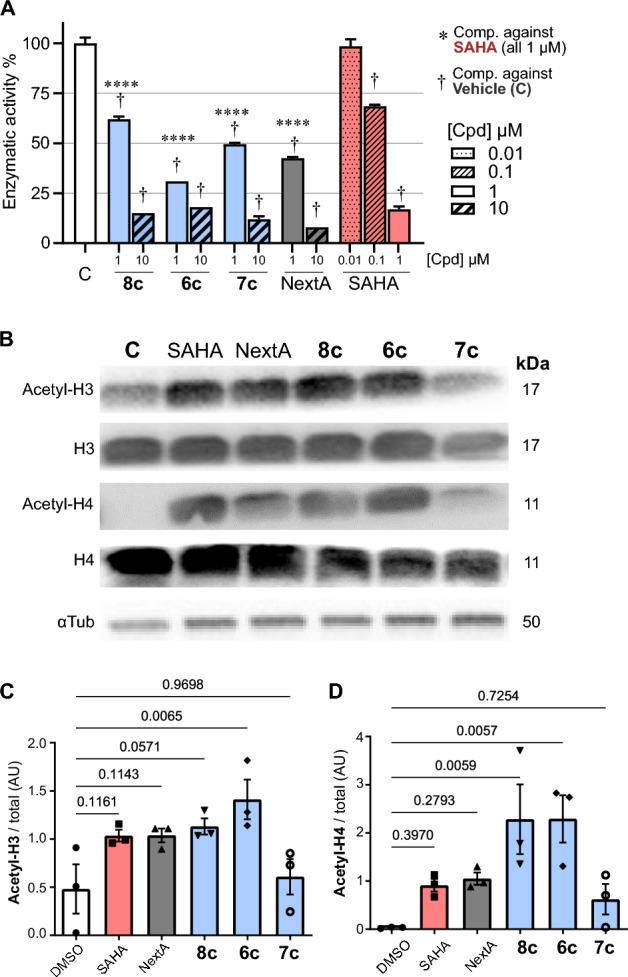
Hydroxamic acid derivatives as potent in vitro* Pf*HDAC1 inhibitors. (**A**) Residual activity (%) of a set of representative inhibitors (**6c–8c**) against recombinant *Pf*HDAC1 tested in two concentrations and compared against the controls NextA (**16**) and SAHA (**1**). Enzyme activity was calculated by normalizing the data against the DMSO treated samples and discounting the background. Data are presented as shown above, with mean ± S.D. (n = 2). Differences to this value were analyzed by one way ANOVA using Dunnett’s method for multiple comparison (asterisks), where ****P < 0.001, comparing against the SAHA 1 µM treatment. Comparison of group/concentration against the DMSO control is represented as † for P < 0.0001. (**B**) Immunoblotting analysis for histones (H3 and H4, unmodified and acetylated), and α-tubulin (αTub) in infected erythrocytes (trophozoites), incubated with DMSO (0.05%, control), SAHA (**1**), NextA (**16**) or compounds **6c–8c**, with 10 × IC_50_ for 4 h, representative gel from N = 3 (see Supporting Informationfor all gels). Numbers on side of the bands represent the predicted mass (kDa) for each protein. (**C**,**D**) Band quantification of histone H3 (**C**) and H4 (**D**), respectively, normalizing their acetylated detection against their unmodified version. Differences to this value were analyzed by one way ANOVA using the Dunnett’s post correction, comparing each group against the DMSO control, as rank tests where P values are explicitly depicted.

Given the high potency of **6c**, **7c**, and **8c** in the *Hs*HDAC panel and the malaria cell-based data, we decided to further characterize their mechanism of action. In this sense, the mode of action of our compounds (**6c**, **7c**, and **8c**) was addressed by an in vitro* Pf*HDAC1 biochemical assay, monitoring their ability to inhibit the lysine acetylation of a model peptide (Fig. 5A). All tested compounds significantly inhibited *Pf*HDAC1 with SAHA **1** being the most potent displaying IC_50_ values between 0.1 and 1 µM. Compounds **7c** and **8c** displayed similar potency to NextA (**16**), inhibiting roughly 50% of the enzyme activity at 1 µM, while **6c** was the most potent with residual activity of 26% on 1 µM.

Further, immunoblotting analysis for acetylated histones (H3 and H4, Fig. 5B and Supporting Information, Fig. S7, Fig. S8) in infected erythrocytes suggest a non-statistically significant increase in acetylated H3 and H4 for SAHA (**1**) and NextA (**16**) treated cells.

Increased H3 acylation did reach statistical significance in the case of compounds **8c** and **7c** (two-fold increase in acetylated-H3), while compounds **8c** and **6c** demonstrated a significant elevation in acetylated-H4 (Fig. 5C, D).

**DMPK assessment** A preliminary DMPK profile has been conducted for compounds **6c**, **7c**, and **8a–d** (Table 4). All compounds were metabolically stable in human liver microsomes (half-life, t_1/2_ > 39 min), though were cleared faster with mouse microsomes (t_1/2_ < 14 min). Noteworthy, **8d** presented the highest t_1/2_ on both human and mouse microsomes (t_1/2_: > 120 and 13.8, respectively). Overall, all compounds did not significantly inhibit four tested CYP450 enzymes that are commonly responsible for human drug first-pass metabolism. Only CYP1A2 was significantly inhibited (22–48% at 10 μM) by **6c**, **7c**, and **8a–b**.

**Table 4:** Microsome stability assessment and CYP inhibition profile of selected HDACis.

Compound	CYP450% inhibition @ 10 µM				Half-life−T = t_1/2_ (in minutes)^a^		Intrinsic clearance—Cl_int_ (µL/min/mg)	
	1A2	2C9	2D6	3A4	Human	Mouse	Human	Mouse
**6c**	32	18	*^b^	*	39.6	4.4	17.0	158
**7c**	22	*	*	*	> 120	4.1	< 6.0	169
**8a**	48	*	*	*	> 120	10.1	< 6.0	69.0
**8b**	42	*	*	*	66.0	4.0	10.0	173
**8c**	*	*	*	*	98.8	2.5	7.0	281
**8d**	*	*	*	*	> 120	13.8	< 6.0	50.0
**Furafylline**^**c**^	81	*	*	*	n.a.	n.a.	n.a.	n.a.
**Sulfaphenazole**	*	94	*	*	n.a.	n.a.	n.a.	n.a.
**Quinidine**	*	*	89	*	n.a.	n.a.	n.a.	n.a.
**Ketoconazole**^**d**^	*	*	*	97	n.a.	n.a.	n.a.	n.a.
**Sunitinib**	n.a.^e^	n.a.	n.a.	n.a.	22.3	9.1	31.0	76.0

^a^Half-life (t_1/2_) in 1 mg/mL hepatic microsomes.

^b^Inhibition < 10%.

^c^Tested concentration: 40 µM.

^d^Tested concentration: 1.0 µM.

^e^n.a.: not applicable.

## Discussion

The preliminary screening of compounds **6a–d**, **7a–c**, and **8a–d** against *P. falciparum* 3D7 strain indicated that the diphenylurea cap-linker moiety has a privileged profile regarding antiplasmodial potency, compared either to cinnamyl or di-hydrocinnamyl derivatives. Compounds **6c**, **7c**, and **8c**, identified as the most active in phenotypic assays (IC_50_ = 1.03 µM; 5.82 µM; and 0.74 µM, respectively, Table 3) and enzymatic inhibition in *Pf*HDAC1 (82%, 88%, and 85% at 10 µM, respectively), exhibited IC_50_ values for HDAC1 of 1584.9 nM, 3556.3 nM, and 6180.2 nM, respectively. Surprisingly, despite **8c** inhibiting *Pf*HDAC1 by 85%, the results obtained for *Hs*HDAC1 revealed it to be the least active. These data indicate a high degree of selectivity towards different isoforms, especially for HDAC1. Noteworthy, the phenyl-hydroxamate and *p*-amino phenyl-hydroxamate moieties are known weak HDAC1 inhibitors, however displaying intrinsic HDAC6 selectivity^31,35^.

This difference in potency might be related to the better interaction among the diphenylurea compounds and the *Pf*HDAC binding cavity. Noteworthy, previous findings for this set of compounds made over a panel of solid and hematological cancer cells have indicated that the cinnamyl linker was the best one to explore human HDACs, thus presenting the most potent activities^26^. Taken together, these findings might indicate that 1,3-diphenylureido hydroxamate is a relevant scaffold to design of potent yet more selective antimalarial HDAC inhibitors, a combination that has been elusive, especially with a hydroxamate ZBG. Curiously, both electron-donating (EDG) and electron-withdrawing (EWG) groups at the *para* position of the capping ring generated potent compounds as **8c** and **8d,** although **8d** was the most potent antiplasmodial compound of the series and the methoxy derivative **8c** presented the highest selectivity index (SI) over human HepG2 cells (SI > 271, Table 1). Noteworthy, the second round of optimized inhibitors gave a significant improvement in selectivity (SI up to > 769, Table 1) without sacrificing potency, as observed for **8e–f**, **8h**, and **8j–k**. On the other hand, compounds **8g** and **8i** had impaired potency/selectivity that could be caused by the presence of the trifluoromethoxy and trifluoromethyl groups, respectively, at the *para* position (Table 1). The importance of the phenylurea cap to the antimalarial potential of the series can be observed by compound **15**, which is the linker-ZBG portion of compound **8a–k**. Even though **15** preserved some activity over 3D7 parasites, lacking the capping motif caused a 24-fold reduction in potency compared to **8k**, thus indicating that the phenylurea moiety is indeed playing a significant role in the way that these compounds interact with the target.

On-target activity confirmation against recombinant *Pf*HDAC1 shows that compounds from all tested scaffolds have potency on the same level as NextA (**16**) but are less potent than SAHA (**1**). While the increased ratio of acetylated-H4 upon treatment confirms **8c** and **6c** target engagement on a cellular level, further improvements in compound permeability are desirable. It is worth noting, however, that compound **8c** exhibited a relevant inhibitory activity against *Pf*HDAC1, while conversely displaying marginal inhibitory action against *Hs*HDAC1.

Our modelling results support the idea that these compounds would bind on both *Pf*HDAC1 and *Hs*HDAC1, with higher predicted binding affinity to *Pf*HDAC1 independently from the scaffold. In *Hs*HDAC1, compounds with a methoxylated cap had weaker hydrophobic interactions than chlorinated ones, which is consistent with their decreased potency in this isoform. Compounds with the Cl-substituted cap perform the worst in terms of predicted energy for the *Hs*HDAC1, in comparison to the Methoxy and unsubstituted counterparts, which agrees with the on-target IC_50_ values (Supporting information, Table S2). *Hs*HDAC6 and *Pf*HDAC1 display higher potency, with no significant differences in the interactions of their cap moieties. The bidentate interaction pattern of the hydroxamate moiety appears to slightly favor hydrophobic interactions in *Pf*HDAC1, which would need to be further supported by calculations that allow polarization and/or QM integration. Additionally, in terms of comparison between predicted binding energy and on-target IC_50_ values, HDAC6 bidentate interaction’s predicted binding energy reflects much better the determined IC_50_s than the monodentate.

The *Pf*HDAC1 is closely related to *Hs*HDAC1, whose main targets are the acetylated H3 and H4, while *Pf*HDAC2 and *Pf*HDAC3 belong to HDAC class II, such as *Hs*HDAC6^18,36^. Our docking models using a *Pf*HDAC6-*like* model supports that longer acyl linkers could function better by occupying its larger pocket, which is in line with the previous *Hs*HDAC activity of these scaffolds. Interestingly, despite our best efforts, no acetylated-α-tubulin, as evidence for HDAC6-like inhibition (*i.e.* HDAC class II inhibition), was detected. We hypothesized that this fact is due to the non-conservation of the acetylation site of the plasmodial homologue, which could result in the no recognition by the human-targeting acetyl-α-tubulin antibody employed.

The unavailability of a recombinant HDAC class II from *Plasmodium falciparum*, together with the discussion that catalytic activity without endogenous cofactors is controversial^37^, supports our concerns that *Pf*HDAC2-3 would be experimentally challenging. Other groups have used human HDAC1 and 6 activity assays as surrogates to assess *Plasmodium*’s HDAC activity^38^. The comparison between our *Pf*HDAC1 inhibitory data (~ 50% inhibition at 1 µM) with this previously published *Hs*HDAC1 dataset (IC_50_ values ranging from 0.9 to 3.2 µM)^29^ would support this correlation. Moreover, our initial compounds^29^ are privileged against *Hs*HDAC6 (with at least ~ ten-fold selectivity against HDAC1), which would encourage further studies on *Plasmodium*’s class II HDACs.

Nardella and co-workers have disclosed novel HDAC–DNA methyltransferase (DNMT) inhibitors, designed by derivatizing the pan-histone deacetylase inhibitor SAHA (**1**) with procainamide^39^. These compounds have acyl linkers with different lengths, with hexyl linkers (n = 6) yielding the most potent derivatives in combination with a basic cap. Shortening of this linker, between the phenyl group and the hydroxamic acid, resulted in a complete loss of antimalarial activity, which disagree with our results, where cinnamyl hydroxamates were less potent antimalarials. It is also important to highlight that Nardella’s design was restricted to saturated acyl linkers, which are flexible and less bulky than our best compounds.

Interestingly, our lead compounds generally retain high potency against the CQ-resistant laboratory *Plasmodium falciparum* strain Dd2. Previous studies, testing HDACi against field-isolated parasites^25,40^ reported higher ex vivo IC_50_ values, when compared to the laboratory 3D7 strain. Despite being tested in similar conditions, strains from Gaboa reported around a three-fold increase in IC_50_ values compared to the laboratory strain^40^, whereas Indonesian isolates were even more resistant with median IC_50_ values ranging from 20–35 times the value found with the 3D7 strain^25^.

Regarding the DMPK assessment, all tested compounds (**6c**, **7c**, and **8a–d**, Table 3) started to be depleted in the absence of NADPH. It is known that NADPH is a required cofactor for cytochrome P450 (CYP450) and flavin monooxygenase enzymatic functions. Moreover, depletion in the absence of NADPH is a strong indication of hydrolysis mediated by other enzymes like proteases and esterases, which is a well-known metabolic target observed for hydroxamates^40^. Besides primary degradation by hydrolysis, the rate of disappearance of compounds **8a–c** was significantly higher upon the addition of NADPH, suggesting the involvement of parallel clearance mechanisms for these compounds. Among all tested CYP450 isozymes, all compounds were mainly susceptible to CYP1A2 which agrees with previous findings for **1** ^41^.

Previous screening of a small library of HDAC inhibitors (180 chemotypes^42^) identified the compound FNDR-20123, among others enriched phenylethyltriazoles, with IC_50_ values against *P. falciparum* culture in the low nanomolar range. FNDR-20123’s phenylethyltriazole cap-linker moiety is a bioisostere from our best-performing phenylurea scaffold, suggesting that a bulkier linker is tolerated, as long as its flexibility is considered. The inhibitor has a high half-life (t_1/2_ 2–9.21 h), higher than **8c**’s by an order of magnitude, which together with their high treatment dosage (50 mg/kg) would have contributed to the consistent reduced parasitaemia. This points out that despite the high antiplasmodial potency of our compounds and excellent target engagement profile, further optimization should rely on minimizing degradation by primary metabolism and favouring ADME properties, mainly solubility. Our best compounds, however, attend to the Malaria Venture lead-like criteria^43^ for antimalarial potency (IC_50_ < 0.1 mM) and parasite selectivity (SI > 100).

## Conclusions

Herein, a series of 1,3-diphenylureido hydroxamates with known HDAC inhibitory activity have been synthesized and evaluated against sensitive and drug-resistant *P. falciparum* strains. Compounds **8a–d** presented potent antiplasmodial activity indicating that the phenyl linker generated compounds with improved potency compared to cinnamyl and di-hydrocinnamyl linkers. Compound **8c** presented the highest SI of the first round of screened compounds and a stable preliminary metabolic profile. In vitro mechanistic studies confirmed *Pf*HDAC1 on-target activity at a recombinant level, consistent with cellular quantification of acetylated histone levels. Notably, compound **8c** demonstrated potent inhibition of *Pf*HDAC1, contrasting with marginal activity against *Hs*HDAC1. In silico studies suggest that the phenyl linker would have the ideal length among the series for interaction with the *Pf*HDAC1 catalytic cavity and that our compound series could bind as well as in *Hs*HDAC1. Taken together, these results highlight the potential of diphenylurea hydroxamates as a privileged scaffold for the generation of potent antimalarial HDAC inhibitors with improved selectivity over human cells.

## Experimental section (methods)

**Chemistry** Chemicals and solvents were purchased from various sources including Merck, Aldrich, Oakwood Chemicals, and Combi-Blocks Inc*.* All reactions sensitive to air and/or water were conducted using dry solvents in anhydrous conditions and under argon atmosphere. The reactions were monitored by thin-layer chromatography (TLC) on Merck silica gel (60 F 254) with UV light (λ = 254 nm). Flash chromatography was carried out using Merck silica gel (particle size 0.040–0.063 nm) on an Isolera Prime system (Biotage). ^1^H and ^13^C NMR spectra were acquired using a 300/75 MHz Bruker spectrometer. The solvent residual peak (DMSO-*d*_*6*_, chemical shifts: 2.50/39.52) served as the internal standard. Analytical High-Performance Liquid Chromatography was performed on a Shimadzu Prominence instrument with the following settings: column, C-18 Gemini (5 μm, 150 × 4.6 mm), mobile phase, 5–100% H_2_O/CH_3_CN containing 0.1% TFA at a flow rate of 1.0 mL/min for 25 min, UV detection at 254 nm. Purity of tested compounds was > 95%, determined through analytical HPLC. All tested compounds were analyzed using a high liquid chromatograph (Shimadzu) coupled to an accurate Q-TOF mass spectrometer, Compact model (Bruker Daltonics), and electrospray ionization interface. Isolated compounds were dissolved in DMSO and subjected to separation using a Kinetex 1.7 μm EVO C18 100 Å (100 × 2.1 mm; Phenomenex Ltd.), with a mobile phase composed of 0.1% formic acid in a mixture of water and acetonitrile. The flow rate was 0.4 mL/min with a gradient program: initial 10% B, 100% B at 5 min, 25% B at 7 min, and a 5 min post-run at 10% B. Injection volume was 20 μL, and column temperature was maintained at 40 °C. The Q-TOF/MS operated in positive mode with specific parameters: ion gas source (N_2_) temperature 200 ℃; nebulizer pressure 45 psi; and capillary voltage of 2,800 V. Mass spectrometer was operated in MS scan mode with internal mass calibration using sodium formate^29^.

**Synthesis of compound 10a.** Ethyl (*E*)-3-(4-(3-phenylureido)phenyl)acrylate (**10a**). General Procedure A: To a solution of ethyl 4-aminocinnamate (**9**) (5 mmol, 1 eq.) in DCM (10 mL), phenyl isocyanate (0.543 mL, 1 eq.) was added. The mixture was stirred at room temperature and argon atmosphere for 16 h. The resulting suspension was filtered *in vacuo*, and washed with DCM (3 $\times$ 30 mL) to afford **10a** as a white solid (1.296 g, 83%). ^1^H NMR (300 MHz, DMSO-*d*_*6*_) δ 8.92 (s, 1H), 8.74 (s, 1H), 7.71–7.43 (m, 7H), 7.30 (t, *J* = 7.7 Hz, 2H), 7.01 (t, *J* = 7.2 Hz, 1H), 6.49 (d,* J* = 15.9 Hz, 1H), 4.19 (q, *J* = 6.9 Hz, 2H), 1.26 (t, *J* = 7.0 Hz, 3H). ^13^C NMR (75 MHz, DMSO-*d*_*6*_) δ 166.4, 152.2, 144.2, 141.9, 139.4, 129.3 (2C), 128.8 (2C), 127.5, 122.0, 118.3 (2C), 117.9 (2C), 115.5, 59.8, 14.2^29^.

**Synthesis of compound 6a.** (*E*)-*N*-hydroxy-3-(4-(3-phenylureido)phenyl)acrylamide (**6a**). General Procedure B: In a round bottom flask, 0.243 g of sodium hydroxide (6.08 mmol, 8 eq.) was dissolved in 1.059 mL of aqueous hydroxylamine solution (50% wt., 38 mmol, 50 eq.) at 0 °C. Then, a solution containing **10a** (0.76 mmol, 1 eq.) in tetrahydrofuran (THF) and methanol (1:1, 6 mL) was added dropwise. The mixture was stirred at room temperature for 2 h. The pH of the mixture was adjusted to 7.0 with the addition of 2.0 N HCl and poured into 20 mL of cold water. The suspension was filtered under vacuum, washed with water (3 $\times$ 30 mL) and dried in a vacuum pump to afford the title compound as a white solid (0.29 g, 99%). mp: 202–204 °C. ^1^H NMR (300 MHz, DMSO-*d*_6_) δ 10.68 (s, 1H), 8.97 (br s, 1H), 8.87 (s, 1H), 8.71 (s, 1H), 7.57–7.42 (m, 7H), 7.29 (t, *J* = 7.9 Hz, 2H), 6.99 (t, *J* = 7.3 Hz, 1H), 6.36 (d, *J* = 15.8 Hz, 1H). ^13^C NMR (75 MHz, DMSO-*d*_6_) δ 163.2, 152.3, 141.0, 139.5, 138.1, 129.1, 128.8 (2C), 128.3 (2C), 122.0, 118.3 (2C), 118.1 (2C), 116.6. HRMS calc. for C_16_H_16_N_3_O_3_: [M + H]^+^, m/z 298.1191. Found 298.1188 ^29^.

**Synthesis of compound 10b.** Ethyl (*E*)-3-(4-(3-(4-chlorophenyl)ureido)phenyl)acrylate (**10b**). General Procedure A was followed using ethyl 4-aminocinnamate (**9**) and 4-chlorophenyl isocyanate to afford the title compound as a white solid (1.223 g, 71%). ^1^H NMR (300 MHz, DMSO-*d*_*6*_) δ 8.95 (s, 1H), 8.88 (s, 1H), 7.70 – 7.45 (m, 7H), 7.34 (d, *J* = 8.8 Hz, 2H), 6.49 (d, *J* = 16.0 Hz, 1H), 4.18 (q, *J* = 7.0 Hz, 2H), 1.26 (t, *J* = 7.1 Hz, 3H). ^13^C NMR (75 MHz, DMSO-*d*_*6*_) δ 166.4, 152.1, 144.1, 141.7, 138.4, 129.3 (2C), 128.6 (2C), 127.6, 125.6, 119.9 (2C), 118.1 (2C), 115.6, 59.8, 14.2^29^.

**Synthesis of compound 6b.** (*E*)-3-(4-(3-(4-chlorophenyl)ureido)phenyl)-*N*-hydroxyacrylamide (**6b**). General Procedure B was followed using intermediate **10b**. The title compound was obtained as a pale yellow solid (0.33 g, 98%). mp: 226 °C—dec. ^1^H NMR (300 MHz, DMSO-*d*_6_) δ 11.27 (br s, 1H), 9.97 (br s, 1H), 9.87 (s, 1H), 9.71 (s, 1H), 7.62–7.26 (m, 9H), 6.37 (d, *J* = 15.7 Hz, 1H). ^13^C NMR (75 MHz, DMSO-*d*_6_) δ 163.2, 152.5, 141.2, 139.0, 138.0, 128.5 (2C), 128.2 (2C), 125.2, 119.8 (2C), 119.6, 118.2 (2C), 116.7. HRMS calc. for C_16_H_15_ClN_3_O_3_: [M + H]^+^, m/z 332.0801. Found 332.0814^29^.

**Synthesis of compound 10c.** Ethyl (*E*)-3-(4-(3-(4-methoxyphenyl)ureido)phenyl)acrylate (**10c**). General Procedure A was followed using ethyl 4-aminocinnamate (**9**) and 4-methoxyphenyl isocyanate to afford the title compound as a white solid (1.62 g, 95%). ^1^H NMR (300 MHz, DMSO-*d*_*6*_) δ 8.85 (s, 1H), 8.55 (s, 1H), 7.71–7.46 (m, 5H), 7.38 (d, *J* = 8.9 Hz, 2H), 6.89 (d,* J* = 8.9 Hz, 2H), 6.48 (d, *J* = 16.0 Hz, 1H), 4.19 (q,* J* = 7.1 Hz, 2H), 3.73 (s, 3H), 1.26 (t, *J* = 7.1 Hz, 3H). ^13^C NMR (75 MHz, DMSO-*d*_*6*_) δ 166.4, 154.7, 152.4, 144.2, 142.2, 132.4, 129.3 (2C), 127.2, 120.2 (2C), 117.8 (2C), 115.3, 114.0 (2C), 59.7, 55.1, 14.2^29^.

**Synthesis of compound 6c.** (*E*)-*N*-hydroxi-3-(4-(3-(4-methoxyphenyl)ureido)phenyl)acrylamide (**6c**). General Procedure B was followed using intermediate **10c**. The title compound was obtained as a white solid (0.327 g, 99%). mp: 210 ºC—dec ^1^H NMR (300 MHz, DMSO-*d*_6_) δ 10.67 (s, 1H), 9.00 (s, 1H), 8.82 (s, 1H), 8.55 (s, 1H), 7.61–7.26 (m, 7H), 6.89 (d, *J* = 8.8 Hz, 2H), 6.36 (d, *J* = 15.8 Hz, 1H), 3.73 (s, 3H). ^13^C NMR (75 MHz, DMSO-*d*_6_) δ 163.2, 154.6, 152.5, 141.2, 138.2, 132.5, 128.3 (2C), 128.1, 120.2 (2C), 118.0 (2C), 116.5, 114.0 (2C), 55.2. HRMS calc. for C_17_H_18_N_3_O_4_: [M + H] + , m/z 328.1297. Found 328.1318^29^.

**Synthesis of compound 10d.** Ethyl (*E*)-3-(4-(3-(4-nitrophenyl)ureido)phenyl)acrylate (**10d**). General Procedure A was followed using ethyl 4-aminocinnamate (**9**) and 4-nitrophenyl isocyanate to afford the title compound as a yellow solid (1.60 g, 90%). ^1^H NMR (300 MHz, DMSO-*d*_*6*_) δ 9.46 (s, 1H), 9.13 (s, 1H), 8.19 (d, *J* = 9.2 Hz, 2H), 7.76–7.60 (m, 5H), 7.59–7.50 (m, 2H), 6.49 (d, *J* = 16.0 Hz, 1H), 4.18 (q, *J* = 7.0 Hz, 2H), 1.26 (t, *J* = 7.1 Hz, 3H). ^13^C NMR (75 MHz, DMSO-*d*_*6*_) δ 166.4, 151.7, 146.1, 144.0, 141.2, 129.3 (2C), 128.1, 125.0 (2C), 118.4 (2C), 117.9, 117.6 (2C), 115.9, 59.8, 14.2^29^.

**Synthesis of compound 6d.** (*E*)-*N*-hydroxy-3-(4-(3-(4-nitrophenyl)ureido)phenyl)acrylamide (**6d**). General Procedure B was followed using intermediate **10d**. The title compound was obtained as a yellow solid (0.32 g, 94%). mp: 167–168 ºC. ^1^H NMR (300 MHz, DMSO-*d*_6_) δ 10.71 (br s, 1H), 9.51 (s, 1H), 9.14 (s, 1H), 9.03 (br s, 1H), 8.19 (d, *J* = 9.0 Hz, 2H), 7.71 (d, *J* = 9.0 Hz, 2H), 7.65–7.39 (m, 5H), 6.39 (d, *J* = 15.7 Hz, 1H). ^13^C NMR (75 MHz, DMSO-*d*_6_) δ 163.1, 151.8, 146.2, 141.1, 140.2, 129.0, 128.3 (2C), 125.1 (2C), 118.6 (2C), 117.9, 117.5 (2C), 117.0. HRMS calc. for C_16_H_15_N_4_O_5_: [M + H] + , m/z 343.1042. Found 343.1065^29^.

**Synthesis of compound 11a.** Ethyl 3-(4-(3-phenylureido)phenyl)propanoate (**11a**). *General Procedure C* In an argonated solution of intermediate **10a** (0.31 g, 1 mmol, 1 eq.) in ethanol (30 mL), 0.24 g of 10% palladium on activated charcoal (10% Pd/C) was added at 0 °C. The resulting mixture was stirred at room temperature under H_2_ atmosphere for 16 h. The product was filtered through a small pad of Celite, and concentrated to afford the product as a white solid (0.312 g, > 99%). ^1^H NMR (300 MHz, DMSO-*d*_*6*_) δ 8.68 (s, 1H), 8.63 (s, 1H), 7.46 (d, *J* = 8.0 Hz, 2H), 7.37 (d, *J* = 7.5 Hz, 2H), 7.28 (t,* J* = 7.3 Hz, 2H), 7.14 (d, *J* = 7.7 Hz, 2H), 6.97 (t, *J* = 6.9 Hz, 1H), 4.06 (q, *J* = 6.9 Hz, 2H), 2.80 (t, *J* = 7.2 Hz, 2H), 2.60 (t, *J* = 7.3 Hz, 2H), 1.17 (t, *J* = 7.0 Hz, 3H). ^13^C NMR (75 MHz, DMSO-*d*_*6*_) δ 166.4, 152.2, 144.2, 141.9, 139.4, 129.3 (2C), 128.8 (2C), 127.5, 122.0, 118.3 (2C), 117.9 (2C), 115.5, 59.8, 14.2^29^.

**Synthesis of compound 7a.** *N*-hydroxy-3-(4-(3-phenylureido)phenyl)propanamide (**7a**). General Procedure B was followed using intermediate **11a**. The title compound was obtained as a white solid (0.22 g, 73%). mp: 304 ºC—dec. ^1^H NMR (300 MHz, DMSO-*d*_6_) δ 9.48 (br s, 1H), 8.84–8.82 (m, 2H), 7.50 (d, *J* = 7.9 Hz, 2H), 7.40 (d, *J* = 8.1 Hz, 2H), 7.31 (t, *J* = 7.7 Hz, 2H), 7.14 (d, *J* = 8.0 Hz, 2H), 6.99 (t, *J* = 7.3 Hz, 1H), 3.39 (br s, 1H), 2.80 (t, *J* = 7.6 Hz, 2H), 2.28 (t, *J* = 7.6 Hz, 2H). ^13^C NMR (75 MHz, DMSO-*d*_6_) δ 168.3, 152.7, 139.9, 137.8, 134.3, 128.7 (2C), 128.4 (2C), 121.6, 118.3 (2C), 118.1 (2C), 34.1, 30.2. HRMS calc. for C_16_H_18_N_3_O_3_: [M + H]^+^, *m/z* 300.1348. Found 300.1369^29^.

**Synthesis of compound 11b.** Ethyl 3-(4-(3-(4-chlorophenyl)ureido)phenyl)propanoate (**11b**). Intermediate **11b** was prepared following the General Procedure C from intermediate **10b**. The title compound was isolated as a white solid (0.33 g, 95%). ^1^H NMR (300 MHz, DMSO-*d*_*6*_) δ 8.64 (s, 1H), 8.59 (s, 1H), 7.49 (d, *J* = 7.7 Hz, 2H), 7.40 (d, *J* = 6.9 Hz, 2H), 7.17 (d, *J* = 7.1 Hz, 2H), 6.99 (d, *J* = 7.4 Hz, 2H), 4.09 (q, *J* = 7.0 Hz, 2H), 2.84 (t, *J* = 7.2 Hz, 2H), 2.62 (t, *J* = 7.3 Hz, 2H), 1.20 (t, *J* = 7.1 Hz, 3H). ^13^C NMR (75 MHz, DMSO-*d*_*6*_) δ 172.2, 152.6, 139.8, 137.8, 133.9, 128.8 (2C), 128.5 (2C), 121.7, 118.3 (2C), 118.2 (2C), 59.8, 35.3, 29.7, 14.1^29^.

**Synthesis of compound 7b.** 3-(4-(3-(4-chlorophenyl)ureido)phenyl)-*N*-hydroxypropanamide (**7b**). General Procedure B was followed using intermediate **11b**. The title compound was obtained as a white solid (0.19 g, 86%). mp: 286 ºC—dec. ^1^H NMR (300 MHz, DMSO-*d*_6_) δ 11.23 (br s, 1H), 9.97 (br s, 1H), 9.14 (s, 1H), 9.09 (s, 1H), 7.49 (d, *J* = 7.7 Hz, 2H), 7.40 (d, *J* = 6.9 Hz, 2H), 7.17 (d, *J* = 7.1 Hz, 2H), 6.99 (d, *J* = 7.4 Hz, 2H), 2.84 (t, *J* = 7.2 Hz, 2H), 2.62 (t, *J* = 7.3 Hz, 2H). ^13^C NMR (75 MHz, DMSO-*d*_6_) δ 168.3, 152.7, 139.9, 137.8, 134.3, 128.7 (2C), 128.4 (2C), 121.6, 118.3 (2C), 118.1 (2C), 34.1, 30.3. HRMS calc. for C_16_H_17_ClN_3_O_3_: [M + H]^+^, *m/z* 334.0958. Found 334.0958^29^.

**Synthesis of compound 11c.** Ethyl 3-(4-(3-(4-methoxyphenyl)ureido)phenyl)propanoate (**11c**). Intermediate **11c** was prepared following the General Procedure C from intermediate **10c**. The title compound was isolated as a white solid (0.34 g, 99%). ^1^H NMR (300 MHz, DMSO-*d*_*6*_) δ 8.51 (s, 1H), 8.45 (s, 1H), 7.40–7.32 (m, 4H), 7.12 (d, *J* = 8.3 Hz, 2H), 6.87 (d, *J* = 8.9 Hz, 2H), 4.05 (q, *J* = 7.1 Hz, 2H), 3.72 (s, 3H), 2.79 (t, *J* = 7.5 Hz, 2H), 2.57 (t, *J* = 7.4 Hz, 2H), 1.17 (t, *J* = 7.1 Hz, 3H). ^13^C NMR (75 MHz, DMSO-*d*_6_) δ 172.2, 154.4, 152.8, 138.0, 133.6, 132.8, 128.5 (2C), 119.9 (2C), 118.2 (2C), 114.0 (2C), 59.7, 55.2, 35.3, 29.7, 14.1^29^.

**Synthesis of compound 7c.** *N*-hydroxy-3-(4-(3-(4-methoxyphenyl)ureido)phenyl)propanamide (**7c**). General Procedure B was followed using intermediate **11c**. The title compound was obtained as a white solid (0.22 g, 90%). mp: 202–204 ºC. ^1^H NMR (300 MHz, DMSO-*d*_6_) δ 10.36 (br s, 1H), 9.81 (br s, 1H), 8.62 (s, 1H), 8.57 (s, 1H), 7.46–7.30 (m, 4H), 7.10 (d, *J* = 7.2 Hz, 2H), 6.87 (d, *J* = 8.0 Hz, 2H), 3.73 (s, 3H), 2.77 (t, *J* = 6.2 Hz, 2H), 2.26 (t, *J* = 6.4 Hz, 2H). ^13^C NMR (75 MHz, DMSO-*d*_6_) δ 168.3, 154.4, 152.8, 137.9, 134.1, 132.9, 128.4 (2C), 119.9 (2C), 118.2 (2C), 113.9 (2C), 55.1, 34.1, 30.2. HRMS calc. for C_17_H_20_N_3_O_4_: [M + H]^+^, *m/z* 330.1453. Found 330.1480^29^.

**Synthesis of compound 13a.** Methyl 4-(3-phenylureido)benzoate (**13a**). The intermediate was prepared following General Procedure A from methyl 4-aminobenzoate (**12**) and phenyl isocyanate. The title compound was isolated as a white solid (0.422 g, 31%). ^1^H NMR (300 MHz, DMSO-*d*_*6*_) δ 9.08 (s, 1H), 8.79 (s, 1H), 7.91 (d, *J* = 6.9 Hz, 2H), 7.61 (d, *J* = 7.0 Hz, 2H), 7.49 (d, *J* = 7.4 Hz, 2H), 7.31 (t, *J* = 6.9 Hz, 2H), 7.01 (t, *J* = 7.0 Hz, 1H), 3.83 (s, 3H). ^13^C NMR (75 MHz, DMSO-*d*_*6*_) δ 165.9, 152.1, 144.4, 139.3, 130.4 (2C), 128.8 (2C), 122.4, 122.2, 118.4 (2C), 117.3 (2C), 51.7^29^.

**Synthesis of compound 8a.** *N*-hydroxy-4-(3-phenylureido)bezamide (**8a**). General Procedure B was followed using intermediate **13a**. The title compound was obtained as a white solid (0.27 g, 99%). mp: 304 ºC—dec. ^1^H NMR (300 MHz, DMSO-*d*_6_) δ 11.07 (s, 1H), 8.90 (s, 2H), 8.74 (s, 1H), 7.72 (d, *J* = 8.6 Hz, 2H), 7.53 (d, *J* = 8.6 Hz, 2H), 7.47 (d, *J* = 8.0 Hz, 2H), 7.30 (t, *J* = 7.8 Hz, 2H), 6.99 (t, *J* = 7.3 Hz, 1H). ^13^C NMR (75 MHz, DMSO-*d*_6_) δ 164.1, 152.3, 142.4, 139.4, 128.8 (2C), 127.8 (2C), 125.8, 122.1, 118.3 (2C), 117.3 (2C). HRMS calc. for C_14_H_14_N_3_O_3_: [M + H]^+^, m/z 272.1035. Found 272.1059^29^.

**Synthesis of compound 13b.** Methyl 4-(3-(4-chlorophenyl)ureido)benzoate (**13b**). The intermediate was prepared following General Procedure A from methyl 4-aminobenzoate (**12**) and 4-chlorophenyl isocyanate. The title compound was isolated as a white solid (0.845 g, 55%). ^1^H NMR (300 MHz, DMSO-*d*_*6*_) δ 9.11 (s, 1H), 8.92 (s, 1H), 7.90 (d, *J* = 8.1 Hz, 2H), 7.59 (d, *J* = 8.1 Hz, 2H), 7.50 (d, *J* = 8.4 Hz, 2H), 7.35 (d, *J* = 8.1 Hz, 2H), 3.83 (s, 3H). ^13^C NMR (75 MHz, DMSO-*d*_*6*_) δ 165.9, 152.1, 144.2, 138.3, 130.4 (2C), 128.6 (2C), 125.8, 122.6, 120.0 (2C), 117.4 (2C), 51.7^29^.

**Synthesis of compound 8b.** 4-(3-(4-chlorophenyl)ureido)-*N*-hydroxybenzamide (**8b**). General Procedure B was followed using intermediate **13b**. The title compound was obtained as a white solid (0.22 g, 70%). mp: 357 ºC—dec. ^1^H NMR (300 MHz, DMSO-*d*_6_) δ 11.06 (s, 1H), 8.95 (s, 1H), 8.90 (s, 2H), 7.71 (d, *J* = 8.3 Hz, 2H), 7.57–7.46 (m, 4H), 7.35 (d, *J* = 8.6 Hz, 2H). ^13^C NMR (75 MHz, DMSO-*d*_6_) δ 164.1, 152.2, 142.2, 138.4, 128.6 (2C), 127.8 (2C), 125.9, 125.6, 119.9 (2C), 117.4 (2C). HRMS calc. for C_14_H_13_ClN_3_O_3_: [M + H]^+^, m/z 306.0645. Found 306.0649^29^.

**Synthesis of compound 13c.** Methyl 4-(3-(4-methoxyphenyl)ureido)benzoate (**13c**). The intermediate was prepared following General Procedure A from methyl 4-aminobenzoate (**12**) and 4-methoxiphenyl isocyanate. The title compound was isolated as a white solid (0.31 g, 20%). ^1^H NMR (300 MHz, DMSO-*d*_*6*_) δ 8.99 (s, 1H), 8.59 (s, 1H), 7.89 (d, *J* = 8.5 Hz, 2H), 7.58 (d, *J* = 8.6 Hz, 2H), 7.37 (d, *J* = 8.7 Hz, 2H), 6.89 (d, *J* = 8.8 Hz, 2H), 3.82 (s, 3H), 3.73 (s, 3H). ^13^C NMR (75 MHz, DMSO-*d*_*6*_) δ 168.3, 154.4, 152.8, 137.9, 134.1, 132.9, 128.4 (2C), 119.9 (2C), 118.2 (2C), 113.9 (2C), 55.1, 51.7^29^.

**Synthesis of compound 8c.** *N*-hydroxy-4-(3–4(-methoxyphenyl)ureido)benzamide (**8c**). General Procedure B was followed using intermediate **13c**. The title compound was obtained as a white solid (0.233 g, 85%). mp: 239 ºC—dec ^1^H NMR (300 MHz, DMSO-*d*_6_) δ 11.04 (s, 1H), 8.88 (s, 1H), 8.82 (s, 1H), 8.54 (s, 1H), 7.70 (d, *J* = 8.6 Hz, 2H), 7.51 (d, *J* = 8.7 Hz, 2H), 7.37 (d, *J* = 8.9 Hz, 2H), 6.88 (d, *J* = 8.9 Hz, 2H), 3.73 (s, 3H). ^13^C NMR (75 MHz, DMSO-*d*_6_) δ 164.2, 154.6, 152.5, 142.6, 132.4, 127.8 (2C), 125.5, 120.2 (2C), 117.1 (2C), 114.0 (2C), 55.2. HRMS calc. for C_15_H_16_N_3_O_4_: [M + H] + , m/z 302.1140. Found 302.1155. Purity: 98% at 254 nm^29^.

**Synthesis of compound 13d.** Methyl 4-(3-(4-nitrophenyl)ureido)benzoate (**13d**). The intermediate was prepared following General Procedure A from methyl 4-aminobenzoate (**12**) and 4-nitrophenyl isocyanate. The title compound was isolated as a yellow solid (1.1 g, 70%). ^1^H NMR (300 MHz, DMSO-*d*_*6*_) δ 9.50 (s, 1H), 9.29 (s, 1H), 8.20 (d, *J* = 8.6 Hz, 2H), 7.92 (d,* J* = 8.1 Hz, 2H), 7.71 (d, *J* = 8.7 Hz, 2H), 7.62 (d, *J* = 8.1 Hz, 2H), 3.83 (s, 3H). ^13^C NMR (75 MHz, DMSO-*d*_*6*_) δ 165.8, 151.7, 145.9, 143.6, 141.3, 130.4 (2C), 125.1 (2C), 123.1, 117.9 (2C), 117.7 (2C), 51.8^29^.

**Synthesis of compound 8d.** *N*-hydroxy-4-(3-(4-nitrophenyl)ureido)benzamide (**8d**). General Procedure B was followed using intermediate **13d**. The title compound was obtained as a yellow solid (0.28 g, 86%). mp: 312 ºC—dec ^1^H NMR (300 MHz, DMSO-*d*_6_) δ 11.10 (s, 1H), 9.49 (s, 1H), 9.15 (s, 1H), 8.93 (s, 1H), 8.20 (d, *J* = 9.0 Hz, 2H), 7.73 (t, *J* = 9.4 Hz, 4H), 7.55 (d, *J* = 8.5 Hz, 2H). ^13^C NMR (75 MHz, DMSO-*d*_6_) δ 164.5, 152.3, 146.6, 142.2, 141.7, 128.3 (2C), 127.0, 125.6 (2C), 118.2 (2C), 118.1 (2C). HRMS calc. for C_14_H_13_N_4_O_5_: [M + H] + , m/z 317.0885. Found 317.0880^29^.

**Synthesis of compound 13e.** Methyl 4-(3-(*p*-tolyl)ureido)benzoate (**13e**). The intermediate was prepared following General Procedure A from methyl 4-aminobenzoate (**12**) and *p*-tolyl isocyanate. The title compound was isolated as a white solid (1.14 g, 80%). HRMS calc. for C_16_H_17_N_2_O_3_: [M + H] + , m/z 285.1234. Found 285.1239.

**Synthesis of compound 8e.** *N*-hydroxy-4-(3-(*p*-tolyl)ureido)benzamide (**8e**). General Procedure B was followed using intermediate **13e**. The title compound was isolated as a white solid (1.13 g, 99%). ^1^H NMR (300 MHz, DMSO-*d*_6_) δ 11.06 (s, 1H), 8.90 (s, 1H), 8.87 (s, 1H), 8.64 (s, 1H), 7.69 (d, *J* = 8.7 Hz, 2H), 7.50 (d, *J* = 8.7 Hz, 2H), 7.34 (d, *J* = 8.4 Hz, 2H), 7.09 (d, *J* = 8.3 Hz, 2H), 2.24 (s, 3H). ^13^C NMR (75 MHz, DMSO-*d*_6_) δ 164.1, 152.3, 142.5, 136.8, 130.9, 129.2 (2C), 127.8 (2C), 125.6, 118.4 (2C), 117.2 (2C), 20.4. HRMS calc. for C_15_H_16_N_3_O_3_: [M + H] + , m/z 286.1186. Found 286.1190.

**Synthesis of compound 13f.** Methyl 4-(3-(4-fluorophenyl)ureido)benzoate (**13f.**). The intermediate was prepared following General Procedure A from methyl 4-aminobenzoate (**12**) and 4-fluorophenyl isocyanate. The title compound was isolated as a white solid (1.07 g, 74%). HRMS calc. for C_15_H_14_FN_2_O_3_: [M + H] + , m/z 289.0983. Found 289.0984.

**Synthesis of compound 8f.** 4-(3-(4-fluorophenyl)ureido)-*N*-hydroxybenzamide (**8f.**). General Procedure B was followed using intermediate **13f**. The title compound was isolated as a white solid (1.06 g, 99%). ^1^H NMR (300 MHz, DMSO-*d*_6_) δ 11.06 (s, 1H), 8.91 (s, 2H), 8.78 (s, 1H), 7.72–7.66 (m, 2H), 7.52–7.49 (m, 2H), 7.48–7.44 (m, 2H), 7.13 (t, *J* = 8.9 Hz, 2H). ^13^C NMR (75 MHz, DMSO-*d*_6_) δ 164.5, 157.9 (d, *J* = 238 Hz, 1C), 152.9, 142.9, 136.2, 128.3, 126.2, 120.6, 120.5, 117.8 (2C), 115.9, 115.7. HRMS calc. for C_14_H_13_FN_3_O_3_: [M + H] + , m/z 290.0935. Found 290.0941.

**Synthesis of compound 13g.** Methyl 4-(3-(4-(trifluoromethoxy)phenyl)ureido)benzoate (**13g**). The intermediate was prepared following General Procedure A from methyl 4-aminobenzoate (**12**) and 4-(trifluoromethoxy)phenyl isocyanate. The title compound was isolated as a white solid (1.1 g, 62%). HRMS calc. for C_16_H_14_F_3_N_2_O_4_: [M + H] + , m/z 355.0900. Found 355.0895.

**Synthesis of compound 8g.** *N*-hydroxy-4-(3-(4-(trifluoromethoxy)phenyl)ureido)benzamide (**8g**). General Procedure B was followed using intermediate **13g**. The title compound was isolated as a white solid (0.66 g, 60%). ^1^H NMR (300 MHz, DMSO-*d*_6_) δ 11.08 (s, 1H), 8.97 (s, 1H), 8.97 (s, 1H), 8.92 (s, 1H), 7.73–7.68 (m, 2H), 7.58–7.55 (m, 2H), 7.53–7.49 (m, 2H), 7.29 (d, *J* = 8.5 Hz, 2H). ^13^C NMR (75 MHz, DMSO-*d*_6_) δ 164.1, 152.3, 142.8, 142.3, 138.8, 130.4, 127.8, 125.9, 121.8 (2C), 120.2 (q, *J* = 238 Hz, 1C) 119.5 (2C), 117.4 (2C). HRMS calc. for C_15_H_13_F_3_N_3_O_4_: [M + H] + , m/z 356.0853. Found 356.0849.

**Synthesis of compound 13h.** Methyl 4-(3-(4-cyanophenyl)ureido)benzoate (**13 h**). The intermediate was prepared following General Procedure A from methyl 4-aminobenzoate (**12**) and 4-cyanophenyl isocyanate. The title compound was isolated as a white solid (0.74 g, 51%). HRMS calc. for C_16_H_14_N_3_O_3_: [M + H] + , m/z 296.1030. Found 296.1045.

**Synthesis of compound 8h.** (*Z*)-*N*-hydroxy-4-(3-(4-(*N*'-hydroxycarbamimidoyl)phenyl)ureido)benzamide (**8h**). General Procedure B was followed using intermediate **13 h**. The title compound was isolated as a white solid (0.49 g, 60%). mp: 97–99 ºC. ^1^H NMR (300 MHz, DMSO-*d*_6_) δ 11.09 (s, 1H), 9.65 (br s, 1H), 8.97 (s, 1H), 8.96 (s, 1H), 8.92 (s, 1H), 7.92–7.89 (m, 2H), 7.78–7.65 (m, 2H), 7.54–7.47 (m, 2H), 7.31–7.27 (m, 2H), 6.01 (br s, 2H). ^13^C NMR (75 MHz, DMSO-*d*_6_) δ 164.5, 159.9, 152.7, 145.1, 142.6, 133.8, 129.4 (2C), 128.3 (2C), 126.6, 118.2 (2C), 117.9 (2C). HRMS calc. for C_15_H_16_N_5_O_4_: [M + H] + , m/z 330.1197. Found 330.1207.

**Synthesis of compound 13i.** Methyl 4-(3-(4-(trifluoromethyl)phenyl)ureido)benzoate (**13i**). The intermediate was prepared following General Procedure A from methyl 4-aminobenzoate (**12**) and 4-(trifluoromethyl)phenyl isocyanate. The title compound was isolated as a white solid (1.27 g, 75%). HRMS calc. for C_16_H_14_F_3_N_2_O_3_: [M + H] + , m/z 339.0951. Found 339.0953.

**Synthesis of compound 8i.** *N*-hydroxy-4-(3-(4-(trifluoromethyl)phenyl)ureido)benzamide (**8i**). General Procedure B was followed using intermediate **13i**. The title compound was isolated as a white solid (0.76 g, 60%). ^1^H NMR (300 MHz, DMSO-*d*_6_) δ 11.09 (s, 1H), 9.19 (s, 1H), 9.06 (s, 1H), 8.93 (s, 1H), 7.74–7.70 (m, 2H), 7.68 (d, *J* = 8.9 Hz, 2H), 7.65 (d, *J* = 9.0 Hz, 2H), 7.55–7.51 (m, 2H). ^13^C NMR (75 MHz, DMSO-*d*_6_) δ 164.5, 152.6, 143.7, 142.5, 128.3, 126.6, 126.5, 125.0, 122.4 (q, *J* = 31.8 Hz), 118.5 (2 C), 118.0 (2 C). HRMS calc. for C_15_H_13_F_3_N_3_O_3_: [M + H] + , m/z 340.0904. Found 340.0910.

**Synthesis of compound 8j.** 4-(3-(4-aminophenyl)ureido)-*N*-hydroxybenzamide (**8j**). General Procedure C was followed using compound **8d** as starting material. The title compound was isolated as a pale yellow solid (0.286 g, 99%). ^1^H NMR (300 MHz, DMSO-*d*_6_) δ 11.09 (s, 1H), 9.19 (s, 1H), 9.06 (s, 1H), 8.93 (s, 1H), 7.74 – 7.70 (m, 2H), 7.68 (d, *J* = 8.9 Hz, 2H), 7.65 (d, *J* = 9.0 Hz, 2H), 7.55 – 7.51 (m, 2H). ^13^C NMR (75 MHz, DMSO-*d*_6_) δ 164.5, 152.6, 143.7, 142.5, 128.3, 126.6, 126.5, 125.0, 122.4, 118.5, 118.0. HRMS calc. for C_15_H_13_F_3_N_3_O_3_: [M + H] + , m/z 340.0904. Found 340.0910.

**Synthesis of compound 15.** 4-amino-*N*-hydroxybenzamide (**15**). (i) To a solution of 4-(boc-amino)benzoic acid (0.24 g, 1 mmol) in DCM (3 mL) was added HATU (0.456 g, 1.2 mmol, 1.2 eq.) under argon atmosphere at room temperature. After stirring at the same temperature for 10 min, *O*-(*tert*-butyldimethylsilyl)hydroxylamine (0.177 g, 1.2 mmol, 1.2 eq.) and DIPEA (0.523 mL, 3.0 mmol, 3 eq.) were added. The resulting mixture was stirred at room temperature for 16 h. The solvent was removed under vacuum, and the crude product was purified by flash chromatography (0 –50% EtOAc/hexane) to afford the Boc-protected intermediate as a white solid (0.110 g, 0.3 mmol). (ii) The preceding intermediate (0.110 g, 0.3 mmol) was placed in a round-bottom flask, and DCM (1 mL) was added followed by TFA (3 mL). The resulting mixture was stirred at room temperature for 3 h. The solvent was removed under vacuum, and the crude product was obtained as a pale yellow solid which was used in the following step without further purification (80 mg, 0.3 mmol, TFA salt). ^1^H NMR (300 MHz, DMSO-*d*_6_) δ 11.25 (br s, 2H), 7.79–7.74 (m, 2H), 7.56 (s, 1H), 7.39 (s, 1H), 7.22 (d, *J* = 8.2 Hz, 2H). ^13^C NMR (75 MHz, DMSO-*d*_6_) δ 164.2, 139.8, 131.5, 128.8 (2C), 120.8 (2C). HRMS calc. for C_7_H_9_N_2_O_2_: [M + H] + , m/z 153.0659. Found 153.0660^44^.

**Synthesis of compound 8 k.** 4-(3-(4-cyanophenyl)ureido)-*N*-hydroxybenzamide (**8 k**). General Procedure A was followed using 4-amino-*N*-hydroxybenzamide (**15**) and 4-cyanophenyl isocyanate to afford the title compound as a pale yellow solid (50 mg, 56%). mp: 220–222 ºC. ^1^H NMR (300 MHz, DMSO-*d*_6_) δ 11.09 (s, 1H), 9.29 (s, 1H), 9.12 (s, 1H), 8.93 (s, 1H), 7.76–7.74 (m, 2H), 7.73–7.70 (m, 2H), 7.66–7.64 (m, 2H), 7.54–7.51 (m, 2H). ^13^C NMR (75 MHz, DMSO-*d*_6_) δ 164.5, 152.4, 144.4, 142.3, 133.8 (2C), 128.3, 126.8, 119.7 (2C), 118.6 (2C), 118.1 (2C), 103.9. HRMS calc. for C_15_H_13_N_4_O_3_: [M + H] + , m/z 297.0982. Found 297.0991.

***Plasmodium falciparum* culture and antiplasmodial activity**. *P. falciparum* 3D7 and Dd2 parasites (Wellcome Trust Dundee) were maintained in continuous culture at 37 °C and an atmosphere consisting of 90% N_2_, 5% O_2_, and 5% CO_2_ as described previously^45^ with modifications^46^. Parasites were maintained in 25 mM 4-(2-hydroxyethyl)-1-piperazineethanesulfonic acid (HEPES) and 11.9 mM sodium bicarbonate buffered RPMI 1640 medium supplemented with D-glucose (11 mM), hypoxanthine (200 μM), Albumax-I (0.5% w/v), and 10 μg/mL gentamicin at 4% haematocrit. Development, parasitaemia, and morphology of parasites were monitored by light microscopy of thin blood smears stained according to the Romanowsky method (Panótico Rápido staining kit; Laborclin, Pinhais, Paraná, Brazil). Parasite cultures were synchronised every second day with sorbitol (5% v/v) for 10 min at 37 °C, prior experiment preparation. Fresh 0^+^ blood was generously provided by “Hospital Novo Atibaia” (Atibaia, SP, Brazil), and approved by the ethics committee at ICB-USP. The antiplasmodial effect of all compounds was validated against *P. falciparum* 3D7 strain conducting SYBR Green I (Invitrogen) drug assays as previously reported^30,46^ as a modification of the original procedure^47^. Briefly, two-fold serial dilutions of compounds were prepared in 96-well plates (N = 3) and incubated for 96 h under normal growth conditions using an initial parasitemia of 0.5% and a haematocrit of 2% in a volume of 100 μL per well. Parasite proliferation was measured by the DNA load via fluorescence, using 100 μL of a lysis buffer with SYBR Green I (0.02% v/v) and incubated for 1 h at room temperature in the dark. Fluorescence was quantified using a CLARIOstar plate reader (BMG Labtech, Germany) at excitation and emission wavelength bands of 485 (± 9) and 530 (± 12) nm, respectively. Focal and gain adjustment was performed using the non-treated controls (highest expected fluorescence signal). Data was acquired via the CLARIOstar (V5.20) and MARS software, manually scaled to 0–100%, and plotted using GraphPad Prism (v9.5.2 for Windows, GraphPad Software, La Jolla California USA, www.graphpad.com). Non-treated parasites, the highest solvent concentration on parasites, CQ on parasites, and the highest drug concentration in the medium were used as controls for maximal growth, solvent control, positive biological control, and native drug fluorescence, respectively.

**Cytotoxicity in human HepG2 cells.**^47^ Immortalised human hepatocytes (HepG2, ATCC® HB-8065™) were maintained in Dulbecco’s modified Eagle medium (DMEM, Atena Biotecnologia) supplemented with 10% (v/v) FBS, 2 mM L-glutamine, 1 mM sodium pyruvate, and Penicillin/Streptomycin. Cells were cultivated under a 5% CO_2_ atmosphere at 37 °C and passaged every 48–72 h using 1 × PBS and 0.25% (w/v) Trypsin-0.53 mM EDTA solution. Cytotoxic effects of compounds were assessed using the cell proliferation reagent WST-1 (Roche; CELLPRO-RO) in a 96-well plate-based screening assay. HepG2 cells were seeded at 10^4^ cells/well (100 μL) in 96-well flat-bottom plates (Sarstedt) the night before the experiment to allow attachment of cells. The next day, two-fold serial dilutions of compounds were prepared in fresh medium in an extra plate, and the medium of cells was replaced by the medium containing the compound dilutions. Non-treated cells (max. proliferation), cells treated with the maximal solvent concentration (DMSO; solvent control), and medium with the maximal compound concentration (native absorbance) used as controls. Plates were incubated for 48 h at 37 °C and 5% CO_2_. Subsequently, 10 μL WST-1 was added to each well and plates were incubated for additional 4 h under standard conditions. WST-1 is a tetrazolium salt and is metabolised by the mitochondrial succinate-tetrazolium-reductase system of living cells and forms formazan, whose absorbance was measured at 450 nm. Absorbance at 630 nm was assessed to check for protein and precipitation background. Both measurements were acquired via the CLARIOstar (V5.20) and MARS software, manually scaled to 0–100%, and plotted with GraphPad Prism (version 9.5.2 for Windows, GraphPad Software, La Jolla California USA).

**In vitro HDAC enzymatic assays.** Recombinant human HDAC1 and HDAC6 were purchased from ENZO Life Sciences AG (Lausen, CH) whereas HDAC8 was produced as described before^33^. In vitro testing of the inhibitors in an enzymatic assay was carried out as described in previous publications^32,33^. For HDAC1 a fluorogenic peptide derived from p53 (Ac-RHKK(Acetyl)-AMC) was used. For HDAC6, the substrate (Abz-SRGGK(thio-TFA)FFRR-NH2) was used as described before^33^. The enzyme inhibition of HDAC8 was determined with a homogenous fluorescence assay and the fluorogenic substrate ZMAL (Z(Ac)Lys-AMC) as described before [J Med Chem, 60 (24) (2017), pp. 10,188–10204]. All measurements were performed in assay buffer (50 mM HEPES, 150 mM NaCl, 5 mM MgCl2, 1 mM TCEP and 0.2 mg/mL BSA, pH 7.4 adjusted with NaOH) at 37 °C. An Envision 2104 Multilabel Plate Reader (PerkinElmer, Waltham, MA), with an excitation wavelength of 380 ± 8 nm and an emission wavelength of 430 ± 8 nm was used to measure the fluorescence intensity.

**In vitro* Pf*HDAC1 enzymatic assay.** *Pf*HDAC inhibition assays were performed by BPS Bioscience (San Diego, CA). Compounds **6c–8c**, and nexturastat A (NextA, **16**) were dissolved in DMSO with the highest concentration at 5 mM. Then, each DMSO solution was directly diluted 10 × fold into the HDAC assay buffer for an intermediate dilution of 10% DMSO in HDAC assay buffer. Then, 5 µL of the intermediate dilution was added to a 50 µL reaction so that the final concentration of DMSO is 1% in all the reactions. The enzymatic reactions for the *Pf*HDAC1 were conducted at 37 ºC for 2 h in a 50 µL mixture containing HDAC assay buffer in duplicate, 5 µg BSA, HDAC substrate (peptide BOC-Ac-Lys-AMC, catalogue number: 50063), *Pf*HDAC1 enzyme, and the tested compound. Enzymatic reactions were stopped by 50 μL/well of 2 × HDAC and the plate was incubated for further 15 min (room temperature). Fluorescence intensity was measured at an excitation of 360 nm and an emission of 460 nm using a Tecan Infinite M1000 microplate reader. All *Pf*HDAC1 activity assays have been performed in duplicates at each tested concentration (1 and 10 μM), besides Vorinostat (SAHA, **1**), which was tested at 0.01, 0.1, and 1.0 μM concentrations. The fluorescent intensity data were analyzed using GraphPad Prism (v9.5.2). In the absence of the compound, the fluorescent intensity (F_t_) in each data set was defined as 100% activity. In the absence of HDAC, the fluorescent intensity (F_b_) in each data set was defined as 0% activity. The percent activity in the presence of each compound was calculated according to the following equation: % activity = (F-F_b_)/(F_t_-F_b_), where “F” is the fluorescent intensity in the presence of the compound.

### Western blotting analysis

**Protein extraction.** After treatment with compounds (10 × IC_50_ for 4 h) and removal of red blood cells by saponin lysis, protein extraction buffer was added (HEPES 10 mM, SDS 1%, MgCl_2_, 6 H2O 1.5 mM, KCl 10 mM, DTT 1 mM, NP-40 0.1%) in the presence of a mixture of protease inhibitors (Amersham Biosciences) and phosphatase (Sigma) and samples frozen -20 °C.

**Western Blot analysis.** Equal amounts of proteins from each extract were solubilized in sample buffer (50 mM Tris–HCl (pH 6.8), 2% SDS, 32% glycerol, 1.5 mM bromophenol blue) and subjected to SDS-PAGE (20%). Proteins were transferred to PVDF membranes, 5% non-fat dry milk in TBS with Tween 20 (0.1%) was used as blocking agent for 1 h at room temperature. After, incubated with the antibodies overnight at 4 °C (Acetyl Histone H3 Lys9 C5B11 Cell Signaling, Acetyl Histone H4 Lys16 E2B8W Cell Signaling, Histone H3 96C10 Cell Signaling and Histone H4 D2X4V Cell Signaling). For the analysis of protein acetylation levels, the membranes were stripped and re-probed with the corresponding anti-total protein. Mouse monoclonal anti-α-Tubulin (B512 Sigma-Aldrich) was used as loading control. Detection was performed by enhanced chemiluminescence using horseradish peroxidase-conjugated secondary antibodies (Vector Laboratories, Burlingame, CA, USA) and SuperSignal TM West Pico PLUS Chemiluminescent substrate kit (Thermo Scientific). Images were acquired using ChemiDoc TM Imaging System (BioRad Laboratories, CA, USA). Quantitative densitometry was carried out using ImageLab software (Bio-Rad Laboratories, CA, USA). The volume density of the chemiluminescent bands was calculated as an integrated optical density × mm^2^ after background correction from each independent experiment (N = 3).

**DMPK evaluation.** To determine stability in hepatic microsomes, the compound (1 μM) was incubated with 1 mg/mL human or mouse hepatic microsomes at 37 °C with continuous shaking^48^. At 0, 5, 10, 20, 40, and 60 min time points, aliquots were removed and acetonitrile was added to quench the reactions and precipitate the proteins. Samples were then centrifuged through 0.45 μm filter plates and half-lives (T_1/2_ s) were determined by LC–MS/MS. To determine cytochrome P450 (CYP450) inhibition, 10 μM compound was incubated with human liver microsomes and selective marker substrates (1A2, phenacetin demethylation to acetaminophen; 2C9, tolbutamide hydroxylation to hydroxytolbutamide; 2D6, bufuralol hydroxylation to 4′-hydroxybufuralol; 3A4, midazolam hydroxylation to 1′-hydroxymidazolam). After a 10 min incubation, the reaction was terminated and the per cent inhibition was determined.

### Molecular modelling

**Homology model and protein preparation.** The human HDAC1 was retrieved from a representative simulation frame of our previous work^29^. The *Plasmodium falciparum* 3D7 HDAC1 homology model was generated from the (UniProt: Q7K6A1_PLAF7, full sequence) using Phyre2 on intensive mode with standard options^49^. Model was validated by checking its Ramachandran plot and overall energy levels, showing low confidence for the C-terminal after His375. Human model of HDAC6 was generated after the *Danio rerio* structure (PDB ID: 6DV0) similarly as described above. All protein structures were prepared using the Protein Wizard Preparation tool, with standard options and the homology model was further refined to remove sterical clashes.

**Molecular docking.** Three-dimensional ligand structures were generated with LigPrep, using Epik to predict their protonation in pH 7.0 ± 1.0, diastereoisomers configuration were derived from the synthesis. The OPLS4 force field was employed for structure generation. Docking was performed using Glide^50,51^ using the Zn^2+^ ion to orient the binding pocket center, employing XP scoring function. Since redocking of vorinostat was poorly performed, for each ligand up to 10 poses were generated, from which we then selected the conformation for MD based on relevant interactions.

**Molecular dynamics simulations.** MD simulations were carried out by using the Desmond engine^52^ with the OPLS4 force-field^53^. The system encompassed the protein–ligand/cofactor complex, a predefined water model (TIP3P)^54^ as a solvent and counterions (Na^+^ or Cl^-^ adjusted to neutralize the overall system charge). The system was treated in a cubic box (13 Å) with a periodic boundary condition (PBC) specifying the size of the box from the box edges to any atom of the protein. Short-range coulombic interactions were calculated using 1 fs time steps and 9.0 Å cut-off value, whereas long-range coulombic interactions were estimated using the Smooth Particle Mesh Ewald (PME) method^55^. Each HDAC + Ligand system was subjected to at least 1 μs simulations (split into five replicas of 200 ns, each) with random seeds. Representative frames of the simulations were retrieved using hierarchical clustering analysis (trj_cluster.py, implemented in Maestro 2023.3, Schrödinger LCC) according to the RMSD of ligand’s heavy atoms (1 Å as cut-off). All the trajectory and interaction data are available on the Zenodo repository (code: 10.5281/zenodo.6984875, made available upon publication). MD trajectories were visualized, and figures were generated using PyMOL v.2.5.2 (Schrödinger LCC, New York, NY, USA).

**MD simulation trajectory analysis.** Protein–ligand interactions and atomic distances were calculated using the Simulation Interaction Diagram analysis pipeline (Maestro 2021.4, Schrödinger LCC). RMSD values of the protein backbone were used to monitor simulation equilibration and protein folding changes (all raw data is available in the repository). MM-GBSA binding energy calculations. Molecular mechanics with generalized Born and surface area (MM-GBSA) predicts the binding free energy of protein–ligand complexes and the ranking of ligands based on the free energy could be correlated to the experimental binding affinities especially in a congeneric series. Every 10th frame from the simulations was considered for energy calculations with thermal_mmgbsa.py script. Calculated free-binding energies were normalized by the number of heavy atoms (HAC), according to the following formula: Ligand Efficiency = (Binding Energy)/(1 + ln(HAC)).

### Statistical analysis

*Western Blot.* Statistical analyses were performed using GraphPad Prism (v9.5.2) (GraphPad Software, San Diego, CA, USA). All results were analyzed for Gaussian distribution and passed the normality test. The statistical differences between the means of the experimental groups were tested through one-way ANOVA analysis followed by Dunnett’s test for multiple comparisons. For all tests, a value of p < 0.05 was considered statistically significant. *Dose–response assays*. Analysis of the IC_50_ values was performed using the nonlinear regression curve fit implemented in GraphPad Prism (v9.5.2) where possible with the four-parameter analysis–variable slope. Residuals were tested for normality via the D'Agostino-Pearson omnibus (K2) test, and for homoscedasticity to check appropriate weighting.

### Supplementary Information

Supplementary Information.

## Data Availability

All data generated or analyzed during this study are included in this published article (and its Supplementary Information files). Moreover, supplementary figures, data collection, further information, refinement statistics as well as ^1^H and ^13^C NMR spectra are available in the “Supporting Information”. All molecular dynamics trajectories and raw data related to the protein–ligand interactions within the simulations are available in the repository: 10.5281/zenodo.6984875.

## Abbreviations

BSA: Bovine serum albumin
CYP450: Cytochrome P450
DCM: Dichloromethane
DIPEA: *N*,*N*-Diisopropylethylamine
DMPK: Drug metabolism and pharmacokinetics
DMSO: Dimethyl sulfoxide
EWG: Electron-withdrawing group
EDG: Electron-donating group
FDA: Food and Drug Administration
HAT: Histone acetyltransferases
HATU: 1-[Bis(dimethylamino)methylene]-1H-1,2,3-triazolo[4,5-b]pyridinium 3-oxide hexafluorophosphate
HDAC: Histone deacetylase
HDACi: Histone deacetylase inhibitor
HPLC: High-performance liquid chromatography
HRMS: High resolution mass spectrometry
IC_50_: Half-maximal inhibitory concentration
NAD: Nicotinamide adenine dinucleotide
NADPH: Nicotinamide adenine dinucleotide phosphate
NMR: Nuclear magnetic resonance
SAHA: Suberoylanilide hydroxamic acid
SAR: Structure–activity relationship
SI: Selectivity index
Sir: Sirtuin
t_1/2_: Half-life
TFA: Trifluoroacetic acid
THF: Tetrahydrofuran
TLC: Thin-layer chromatography
ZBG: Zinc-binding group
