# Supplementary material for: 1,3-Diphenylureido hydroxamate as a promising scaffold for generation of potent antimalarial histone deacetylase inhibitors
Source: Sci Rep. 2023 Nov 29;13:21006. doi: 10.1038/s41598-023-47959-z (PMC10687260; doi:10.1038/s41598-023-47959-z)
Supplement: Supplementary file 1 — Supplementary Information. [file 41598_2023_47959_MOESM1_ESM.docx]

**Supporting Information**

**1,3-diphenylureido hydroxamate as promising scaffold for generation of potent antimalarial histone deacetylase inhibitors**

Maurício T. Tavares,^3,†^ Arne Krüger,^4,†^ Sun L. Rei Yan,^4^ Karoline B. Waitman,^1^ Vinícius M. Gomes,^5^ Daffiny Sumam de Oliveira,^4^ Franciarli Paz,^4^ Sebastian Hilscher,^6^ Mike Schutkowski,^6^ Wolfgang Sippl,^6^ Claudia Ruiz,^2^ Mônica F. Z. J. Toledo,^1^ Neuza M. A. Hassimotto,^7^ João A. Machado-Neto,^8^ Antti Poso,^9^ Michael D. Cameron,^2^ Thomas D. Bannister,^2^ Giuseppe Palmisano,^5^ Carsten Wrenger,^4,*^ Thales Kronenberger,^9,*^ Roberto Parise-Filho^1,*^

^1^ Department of Pharmacy, Faculty of Pharmaceutical Sciences, University of São Paulo, São Paulo, Brazil.

^2^ Department of Molecular Medicine, The Herbert Wertheim Institute for Biomedical Innovation and Technology, Jupiter, Florida 33458, United States.

^3^ a. Department of Cancer Biology, Dana-Farber Cancer Institute, Boston, Massachusetts 02115, United States. b. Department of Biological Chemistry and Molecular Pharmacology, Harvard Medical School, Boston, Massachusetts 02115, United States.

^4^ Unit for Drug Discovery, Department of Parasitology, Institute of Biomedical Sciences, University of São Paulo, São Paulo, Brazil.

^5^ a. GlycoProteomics laboratory, Department of Parasitology, Institute of Biomedical Sciences, University of Sao Paulo, Sao Paulo, Brazil. b. School of Natural Sciences, Faculty of Science and Engineering, Macquarie University, Sydney, Australia.

^6^Faculty of Biosciences, Martin-Luther-University of Halle-Wittenberg, 06120 Halle/Saale, Germany.

^7^Food Research Center-(FoRC-CEPID) and Department of Food Science and Nutrition, Faculty of Pharmaceutical Science, University of São Paulo, São Paulo, SP, Brazil.

^8^ Department of Pharmacology, Institute of Biomedical Sciences, University of São Paulo, São Paulo, Brazil.

^9^a. Department of Pharmaceutical and Medicinal Chemistry, Institute of Pharmaceutical Sciences, Eberhard-Karls-Universität, Tuebingen, Auf der Morgenstelle 8, 72076 Tuebingen, Germany. b. Tuebingen Center for Academic Drug Discovery & Development (TüCAD_2_), 72076 Tuebingen, Germany. c. School of Pharmacy, Faculty of Health Sciences, University of Eastern Finland, P.O. Box 1627, FI-70211 Kuopio, Finland.

^†^M.T.T. and A.K. contributed equally to this work.

*Corresponding Authors:

C.W.: cwrenger@icb.usp.br, Av. Prof. Lineu Prestes 1374, CEP: 05508-900, São Paulo (Brazil).

T.K.: thales.kronenberger@uni-tuebingen.de, Auf der Morgenstelle 8, DE72076, Tübingen (Germany).

R.P.: roberto.parise@usp.br, Av. Prof. Lineu Prestes 580, CEP: 05508-000, São Paulo (Brazil).

**Table of Contents**

[1 Supplementary Figures and Tables S3](#_Toc62049130)

[2 NMR spectra S13](#_Toc62049132)

[3 Chromatograms S4](#_Toc62049158)3

[4 Mass spectra S5](#_Toc62049171)4

# **Supplementary Figures and Tables**


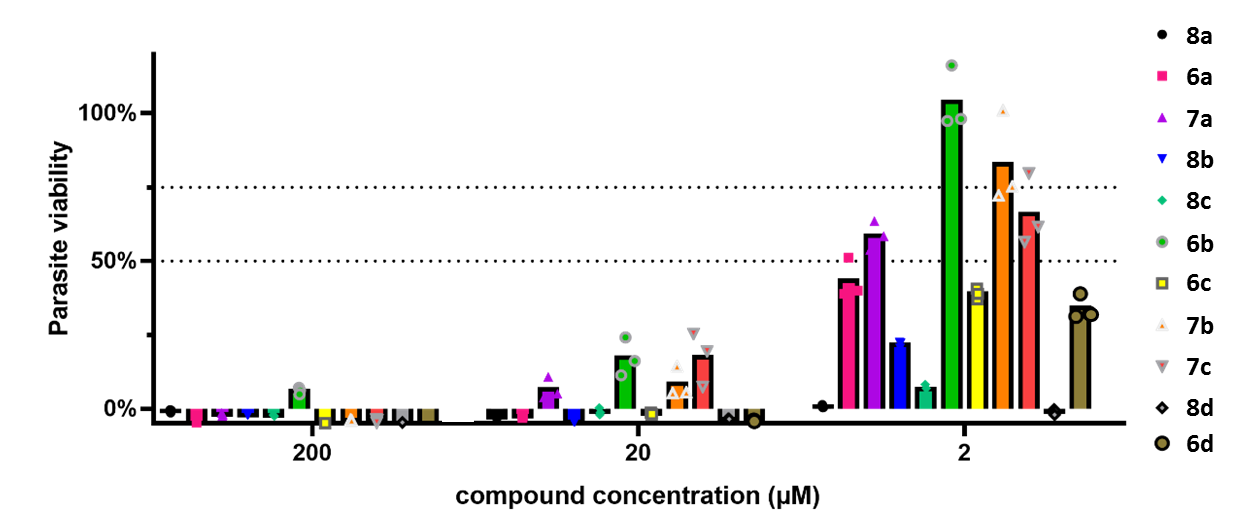


**Figure S1.** Preliminary screening of **6a-d**, **7a-c**, and **8a-d** against *P. falciparum*. Compounds were tested in three different concentrations (200, 20, and 2 μM) using the SYBR Green I fluorescence assay to determine parasite proliferation.


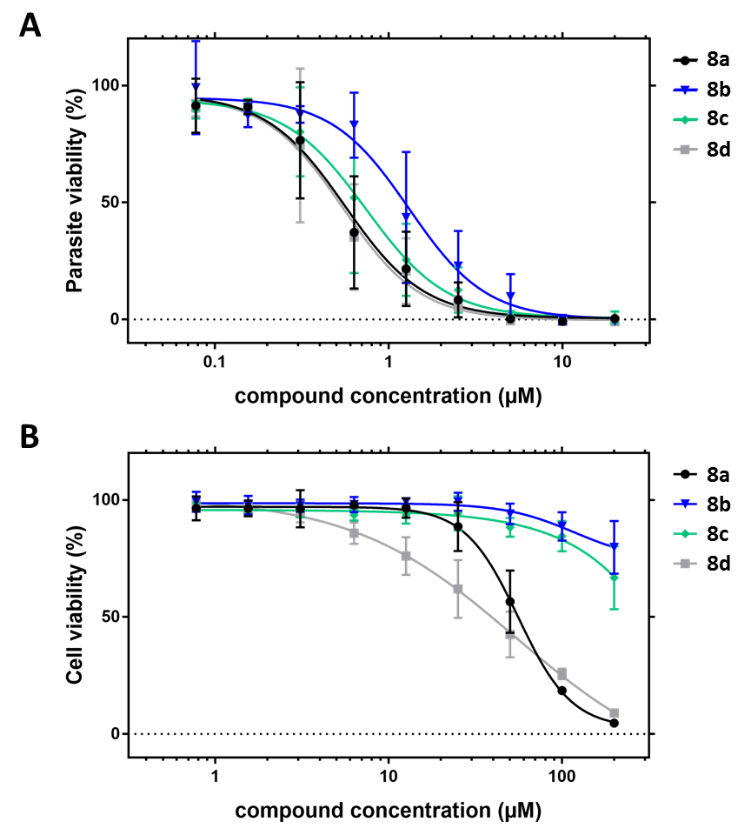


Figure S2. Antiplasmodial effect and cytotoxicity of compounds 8a-d. (A) Antiplasmodial activity against *P. falciparum* 3D7. Compounds were tested as DMSO stocks in a two-fold serial dilution from 20 µM to 78 nM and proliferation of the parasite was assessed by measuring dsDNA using the SYBR Green I assay (Smilkstein et al. 2004, with modifications). (B) Human cytotoxic effect of 8a-d over HepG2 cells. Compounds were tested as DMSO stocks in a two-fold serial dilution from 200 µM to 0.78 µM and viability of cells was assessed by measuring metabolic activity using a WST-1 (Roche) reagent-based assay. 8a (●), 8b (▼), 8c (♦), and 8d (■) curves calculated with GraphPad Prism 7.0 using non-linear regression based on means from three independent experiments measured in triplicate. Error bars indicate standard deviation. For curves that do not reach the zero-point IC_50_ was not calculated and is indicated as an approximation.


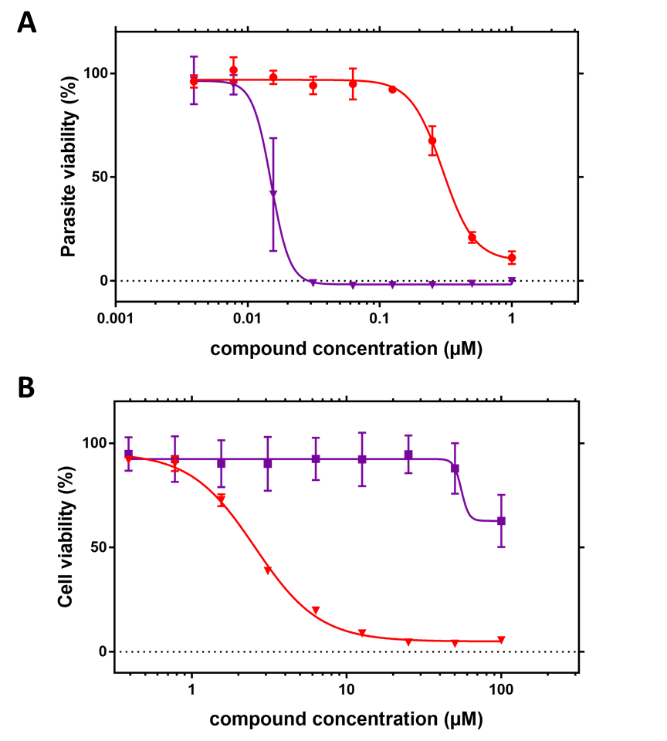


Figure S3. Antiplasmodial effect and cytotoxicity of chloroquine (CQ, in purple) and vorinostat (SAHA, 1, in red). (A) Antiplasmodial activity against *P. falciparum* 3D7. Compounds were tested as DMSO stocks in a two-fold serial dilution from 1 µM to 3.9 nM and proliferation of the parasite was assessed by measuring dsDNA using the SYBR Green I assay (Smilkstein et al. 2004, with modifications). (B) Human cytotoxic effect of CQ and SAHA over HepG2 cells. Compounds were tested as DMSO stocks in a two-fold serial dilution from 100 µM to 0.39 µM and viability of cells was assessed by measuring metabolic activity using a WST-1 (Roche) reagent-based assay. CQ (▼) and SAHA (●) curves calculated with GraphPad Prism 7.0 using non-linear regression based on mean from one experiment measured in triplicate. Error bars indicate standard deviation. For curves that do not reach the zero-point IC_50_ was not calculated and is indicated as an approximation.


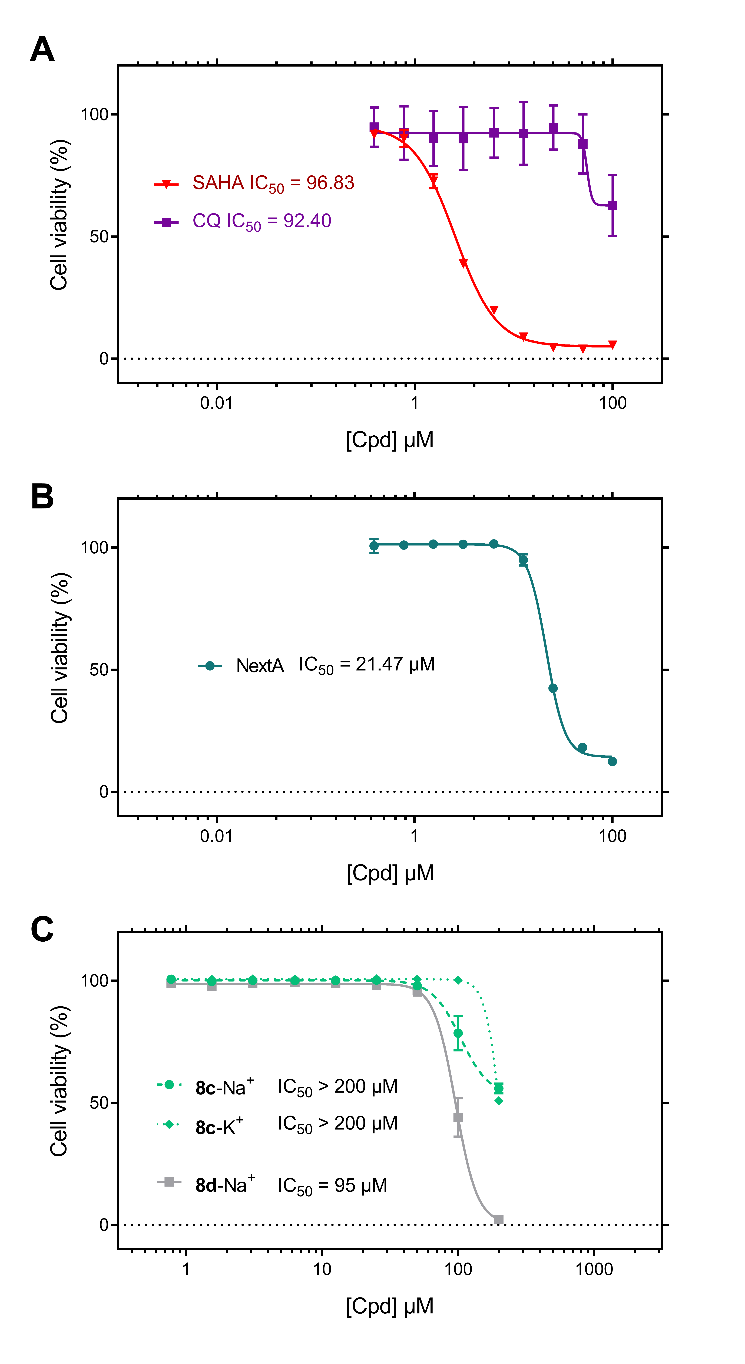


**Figure S4.** Human cytotoxic effect of 8a-d over HepG2 cells. Compounds were tested as DMSO stocks in a two-fold serial dilution from 200 µM to 0.78 µM and viability of cells was assessed by measuring metabolic activity using a WST-1 (Roche) reagent-based assay. Curves calculated using non-linear regression based on means from three independent experiments measured in triplicate. Error bars indicate standard deviation. For curves that do not reach the zero-point IC_50_ was not calculated and is indicated as an approximation.


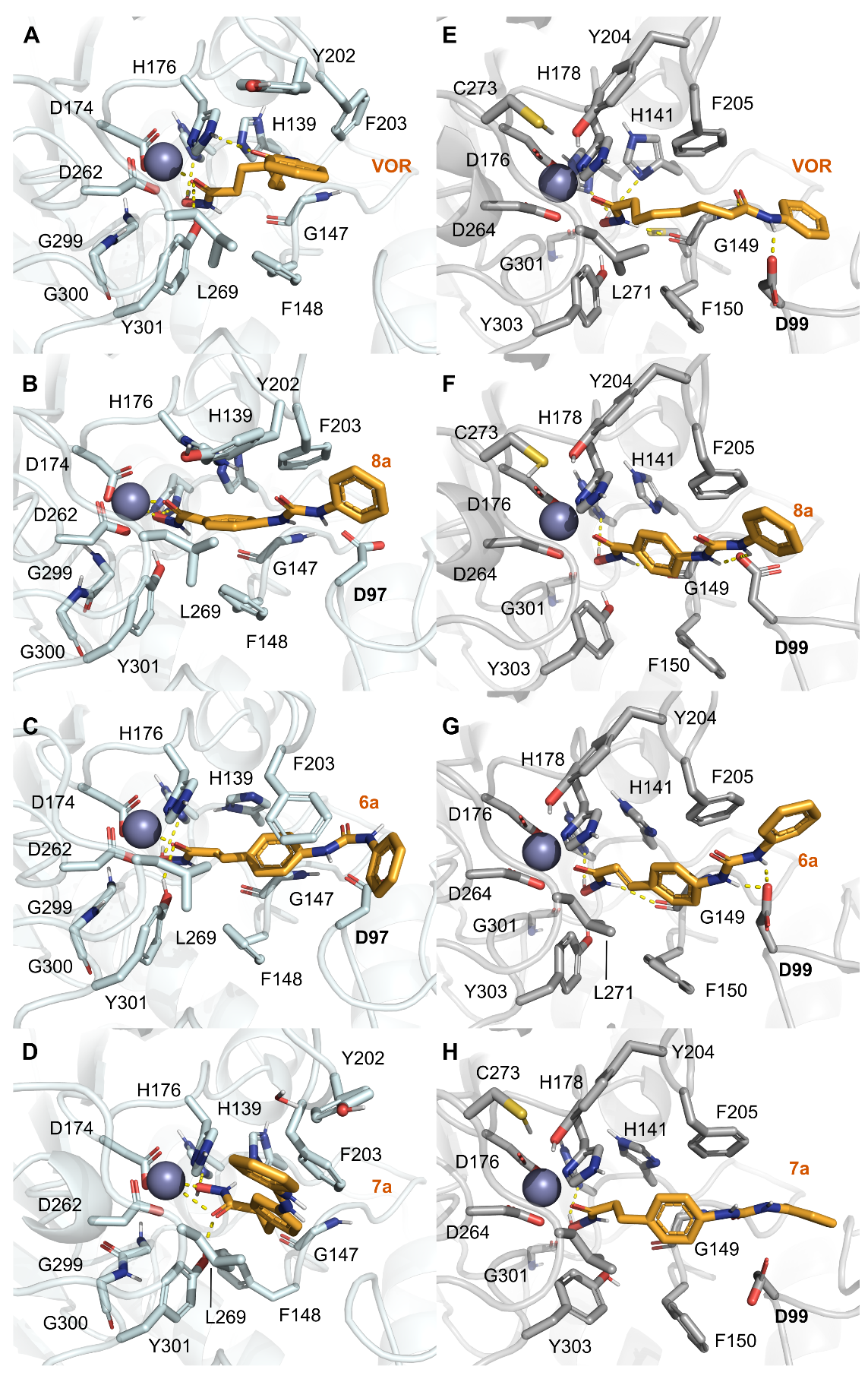


**Figure S5.** Representative binding modes for the *Pf*HDAC1 (**A-D**) and *Hs*HDAC1 (**E-H**) with our compounds *versus* vorinostat simulations.

**Table S1.** Full length of the Protein-ligand interaction frequency during the analyzed trajectory for each compound. Frequency is displayed as (%) of the hydrogen bond, water-mediated interactions or pi-pi interactions and separated according to the compound moiety performing it. W – represents water-mediated hydrogen bonds.

| HsHDAC6/**PfHDAC1** | |  | **HsHDAC1 - MO** | | | |  | **HsHDAC6-MO** | | | | **HsHDAC6-BI** | | |  |  |  | **PfHDAC-MO** | | | **PfHDAC-BI** | | |
| --- | --- | --- | --- | --- | --- | --- | --- | --- | --- | --- | --- | --- | --- | --- | --- | --- | --- | --- | --- | --- | --- | --- | --- |
| HsHDAC1 | | **VOR** | **NextA** | **6c** | **7c** | **8c** |  | **6c** | **7c** | **8c** | **CTR** | **6c** | **7c** | **8c** |  | VOR | NextA | **6c** | **7c** | **8c** | **6c** | **7c** | **8c** |
| **Cap - Hydrophobic** | P23/**P25**/P29 | 0.9 | 16.84 | 1.3 | 12 | 4.6 |  | 4.6 | 3.4 | 8.3 | 0.9 | 8.3 | 32.5 | 21.2 |  | 9.4 | 12 | 1.3 | 6.1 | 2 | 8 | 5.5 |  |
|  | F142/**F148**/F150 | 12.3 | 21.88 | 6 | 15.3 | 8.4 |  | 25.6 | 11.6 | 33.9 | 17.9 | 20.3 | 21.1 | 31.8 |  | 11.5 | 17.3 | 6.8 | 8.2 | 10 | 14.8 | 11.8 | 3.1 |
|  | F202/**F203**/F205 | 49.9 | 16.76 | 23.3 | 10.1 | 45.6 |  | 21.9 | 32.1 | 31.9 | 11.9 | 19.9 | 2.4 | 3 |  | 6.9 | 5 | 22.9 | 26.4 | 8.2 | 14.6 | 30.6 | 25.8 |
|  | L271/**L269**/L271 | 9.2 | 34.72 | 18.6 | 14.4 | 11.1 |  | 0.7 | 4.9 | 6.1 | 3.7 | 10.4 | 9.7 | 2.4 |  | 12.4 | 21.9 | 14.6 | 16.7 | 7.9 | 9.9 | 6.6 | 6.7 |
|  | Y304/**Y301**/Y303 | 5.4 | 27.72 | 27.9 | 37 | 29.7 |  | 5.2 | 7.4 | 4.3 | 2.8 | 13.2 | 25.7 | 10.5 |  | 28.3 | 25.2 | 4.8 | 10.6 | 7.3 | 21.9 | 0.7 | 0.4 |
|  |  |  |  |  |  |  |  |  |  |  |  |  |  |  |  |  |  |  |  |  |  |  |  |
| **pi-mediated** | H21 |  |  |  |  |  |  | 10 |  |  |  |  | 18 |  |  |  |  |  |  |  |  |  |  |
|  | H22/H28 |  | 7.56 |  |  |  |  | **16** |  | **27** |  |  | **12** | **24** |  |  |  |  |  |  |  |  |  |
|  | H142/**H139**/H141 | 0.6 | 1.0 |  | 16.0 |  |  |  |  | 39.0 |  |  |  |  |  |  |  | 20.1 | 11.5 | 46.6 | 9.5 | 29.8 | 9.6 |
|  | F142/**F148**/F150 | 12.4 | 36.6 |  | 14.0 | 11.0 |  | 19.0 |  | 39.0 | 20.0 | 17.0 | 17.0 | 21.0 |  | 4.4 | 10.0 | 2.2 | 2.0 | 6.7 | 7.5 | 6.3 | 1.0 |
|  | H173/**H176**/H178 | 1.3 | 10.5 | 32.0 | 10.0 | 27.0 |  |  | 21 |  | 31.0 | 14.0 |  |  |  | 5.4 | 31.0 | 13.9 | 31.8 | 40.2 | 15.3 | 37.7 | 19.6 |
|  | F202**/F203**/F205 | 1.4 | 6.4 |  |  |  |  | 49.0 | 47 |  | 41.0 | 23.0 |  |  |  | 1.7 |  | 16.6 | 13.5 | 1.0 | 10.2 | 9.3 | 20.2 |
|  | Y204 | 35.0 | 3.3 |  |  |  |  |  |  |  |  |  |  |  |  |  |  |  |  |  |  |  |  |
|  | F304/**Y301**/Y303 | 0.7 | 11.6 | 11.0 |  | 12.0 |  |  |  |  |  |  |  | 15.0 |  | 0.5 |  | 1.4 | 6.2 | 2.7 | 4.9 | 4.7 | 0.1 |
|  |  |  |  |  |  |  |  |  |  |  |  |  |  |  |  |  |  |  |  |  |  |  |  |
| **ZGB - polar** | G141/**G136**/G138 | 42w |  | 12 |  | 38 |  |  |  |  | 38 | 20 | 14w |  |  | 15w | 10w | 13w | 39 | 20w | 11w | 22w |  |
|  | H132/**H138**/H140 | 14w |  |  |  | 35 |  |  | 34 |  | 41 |  |  |  |  | 56 |  | 29 | 15 | 15 | 75 | 77 | 50 |
|  | H133/**H139**/H141 |  |  | 14w |  | 19 |  | 50 |  | 33 |  |  | 37 | 60 |  |  |  |  | 12 |  | 23 | 13 | 15 |
|  | Q260 | 52 |  | 22 |  |  |  |  |  |  |  |  |  |  |  |  |  |  |  |  |  |  |  |
|  | E301/**E262** |  |  |  |  |  |  | 57 |  | 28 |  | 18 | 17 |  |  | 16 |  |  |  |  |  |  |  |
|  | G303/**G299**/G301 | 41 |  | 39 | 31 | 51 |  | 24w |  |  | 13 | 16 |  |  |  | 25 | 40 | 65 |  | 10 | 52 | 17 | 33 |
|  |  |  |  |  |  |  |  |  |  |  |  |  |  |  |  |  |  |  |  |  |  |  |  |
| **Linker - polar** | S90/**D97**/D99 |  |  |  | 29 |  |  | **16** |  | **86** |  | **12** |  | **51** |  |  |  | **32** |  | **13** | **39** | **14** | **23** |
|  | **H176**/H178 | 56 |  |  |  | 22w |  |  |  |  |  |  |  |  |  | 13 | 13w | 57 |  | 58 | 60 | 66 | 67 |
|  | F205 |  |  | 29 |  | 14w |  |  |  |  |  |  |  |  |  |  |  |  | 50 |  |  |  |  |
|  | Y304/**Y301**/Y303 | 13 |  |  | 17 |  |  |  |  |  | 13 |  |  |  |  |  | 14 | 37 | 23 |  |  | 17 |  |

**Table S2**. Mean and standard deviation of the binding energy prediction (Kcal/mol normalized by the Heavy Atoms Count, HAC) calculated using MM/GBSA along the molecular dynamics simulation trajectories (5x200 ns)

|  |  | **Lipophilic terms** | | | | | | | | | |
| --- | --- | --- | --- | --- | --- | --- | --- | --- | --- | --- | --- |
|  |  | **HDAC1** | | **HDAC6-MO** | | **HDAC6-BI** | | **PfHDAC-MO** | | **PfHDAC-BI** | |
| **CPD** | **R_1_** | **AVG** | **SD** | **AVG** | **SD** | **AVG** | **SD** | **AVG** | **SD** | **AVG** | **SD** |
| **6a** | H | -14.28 | 2.7 |  |  |  |  |  |  | -12.11 | 3.1 |
| **6b** | Cl | -13.14 | 2.2 |  |  |  |  |  |  | -14.46 | 3.9 |
| **6c** | **OCH_3_** | -14.19 | 2.1 | -15.01 | 2.87 | -17.05 | 2.0 | -12.91 | 3.3 | -13.08 | 2.7 |
| **7a** | H | -13.89 | 2.1 |  |  |  |  |  |  | -14.58 | 3.7 |
| **7b** | Cl | -16.01 | 3.8 |  |  |  |  |  |  | -12.43 | 2.9 |
| **7c** | **OCH_3_** | -13.87 | 2.8 | -15.27 | 2.6 | -12.21 | 2.1 | -13.76 | 3.5 | -10.82 | 3.7 |
| **8a** | H | -10.97 | 2.9 |  |  |  |  |  |  | -13.08 | 2.6 |
| **8b** | Cl | -14.03 | 2.4 |  |  |  |  |  |  | -11.20 | 3.3 |
| **8c** | **OCH_3_** | -15.76 | 1.9 | -14.48 | 1.9 | -11.40 | 2.4 | -11.16 | 3.0 | -11.65 | 3.4 |
| 16 | Next | -15.08 | 3.9 |  |  |  |  |  |  | -14.40 | 3.00 |
| 1 | VOR | -13.94 | 2.8 |  |  |  |  |  |  | -12.72 | 3.3 |
|  |  |  |  |  |  |  |  |  |  |  |  |
|  |  | **Ligand Efficiency** | | | | | | | | | |
|  |  | **HDAC1** | | **HDAC6-MO** | | **HDAC6-BI** | | **PfHDAC-MO** | | **PfHDAC-BI** | |
| **CPD** | **R_1_** | **AVG** | **SD** | **AVG** | **SD** | **AVG** | **SD** | **AVG** | **SD** | **AVG** | **SD** |
| **6a** | H | -4.65 | 2.1 |  |  |  |  |  |  | -1.35 | 2.1 |
| **6b** | Cl | -3.64 | 1.6 |  |  |  |  |  |  | -3.60 | 1.8 |
| **6c** | **OCH_3_** | -4.36 | 2.5 | -2.01 | 2.3 | -3.37 | 3.1 | -2.26 | 2.3 | -2.73 | 2.9 |
| **7a** | H | -5.74 | 1.9 |  |  |  |  |  |  | -2.57 | 2.3 |
| **7b** | Cl | -3.39 | 2.0 |  |  |  |  |  |  | -2.64 | 1.7 |
| **7c** | **OCH_3_** | -4.62 | 1.7 | -0.83 | 2.4 | -1.31 | 2.1 | -2.73 | 2.1 | -0.81 | 2.8 |
| **8a** | H | -4.86 | 2.5 |  |  |  |  |  |  | -0.62 | 2.4 |
| **8b** | Cl | -4.81 | 2.0 |  |  |  |  |  |  | -1.45 | 2.2 |
| **8c** | **OCH_3_** | -4.43 | 1.5 | -3.69 | 2.5 | -1.29 | 2.4 | -1.51 | 2.6 | -0.80 | 2.4 |
| 16 | Next | -3.27 | 2.1 |  |  |  |  |  |  | -2.78 | 2.2 |
| 1 | VOR | -5.02 | 2.1 |  |  |  |  |  |  |  |  |


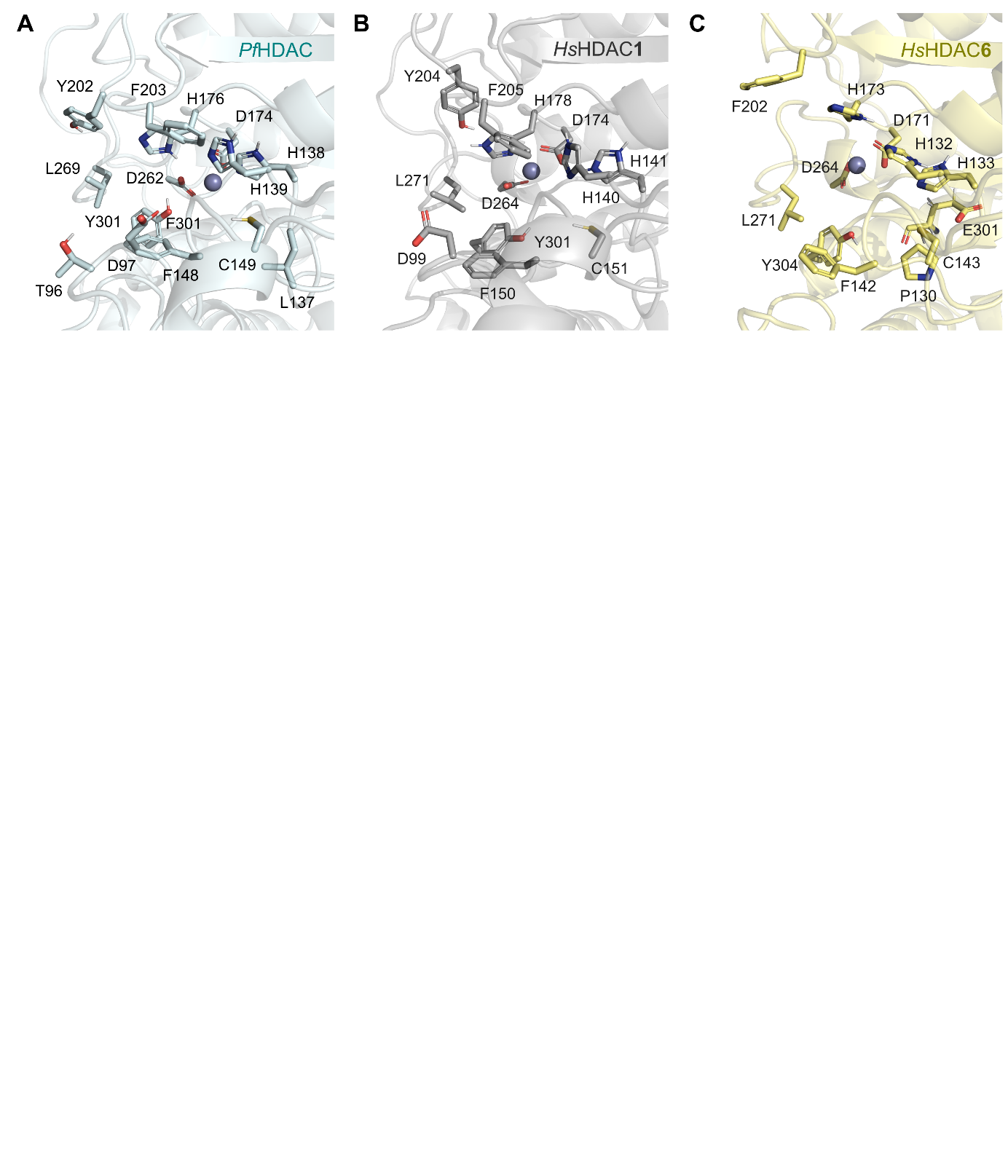


**Figure S6**. Comparison between the amino acid composition of PfHDAC1 (A), HsHDAC1 (B) and HsHDAC6 (C)


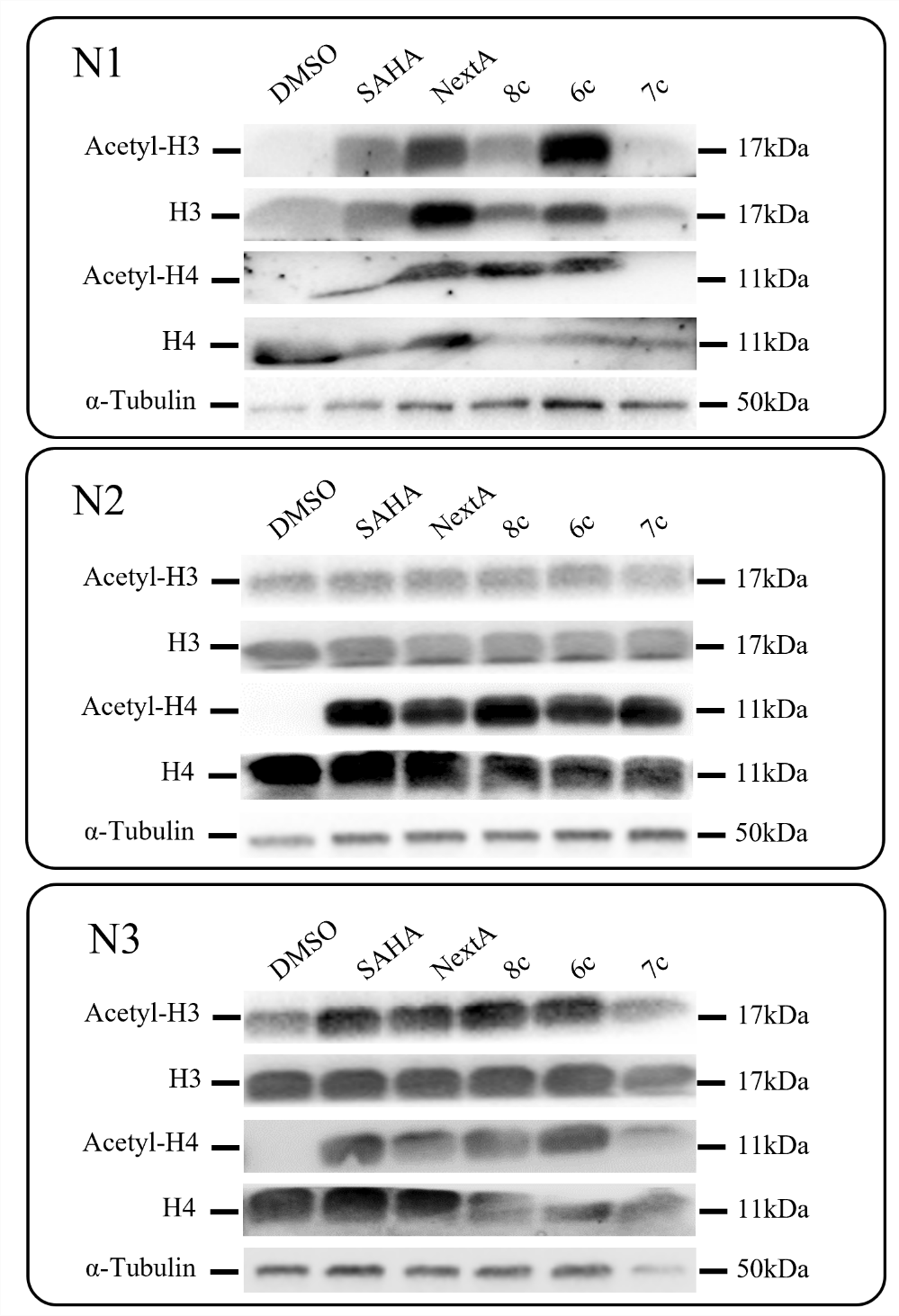


**Figure S7**. Immunoblotting analysis for histones (H3 and H4, unmodified and acetylated), and α-tubulin (αTub) in infected erythrocytes, incubated with **DMSO** (0.05%, control), **SAHA**, **NextA** or compounds **6c**, **7c** or **8c**, with 10x IC_50_ for 4 h, representative gel from N=3, each gel displayed separated N’s 1 – 3. Numbers on side of the bands represent the predicted mass (kDa) for each protein. Raw unedited plots are provided as a separated file.

**
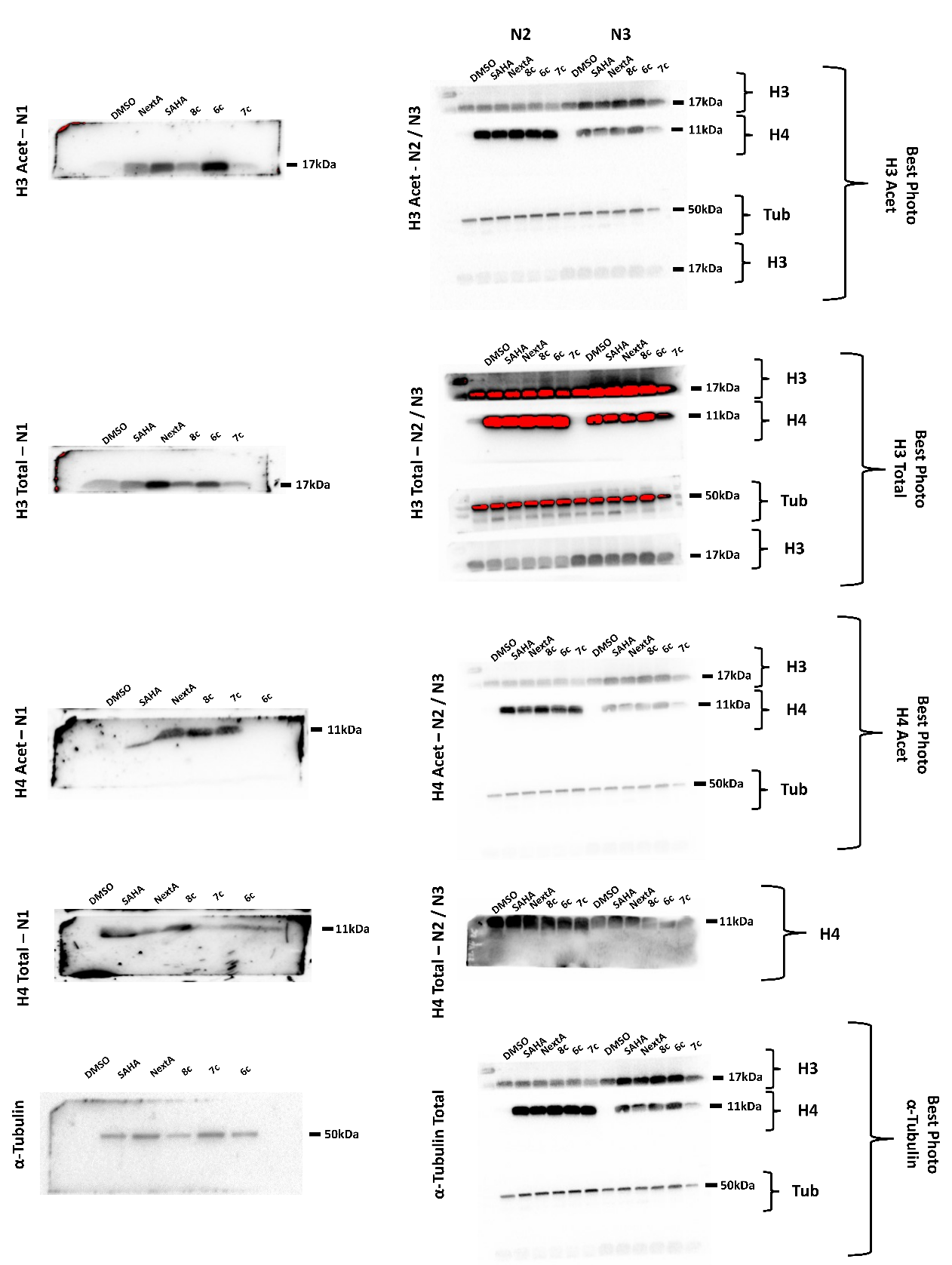
**

**Figure S8**. Raw data for the immunoblotting analysis for histones (H3 and H4, unmodified and acetylated), and α-tubulin (αTub) in infected erythrocytes, incubated with **DMSO** (0.05%, control), **SAHA**, **NextA** or compounds **6c**, **7c** or **8c**, with 10x IC_50_ for 4 h, representative gel from N=3, each gel displayed separated N’s 1 – 3. It is relevant to highlight that each immunoblotting was incubated with all antibodies at the same time, no membrane stripping was involved, and that for each two photos were taken, which were chosen randomly for analyses. Numbers on side of the bands represent the predicted mass (kDa) for each protein. Raw unedited plots are provided as a separated file.

# **NMR spectra**

## Ethyl (*E*)-3-(4-(3-phenylureido)phenyl)acrylate (**10a**)


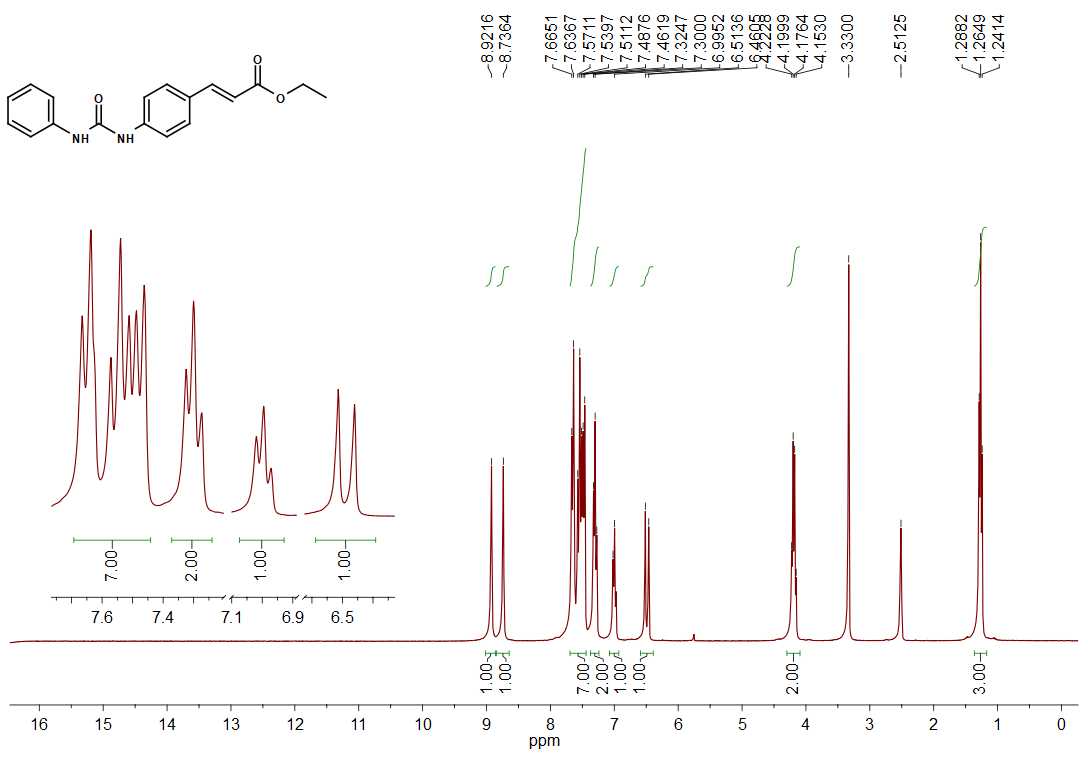


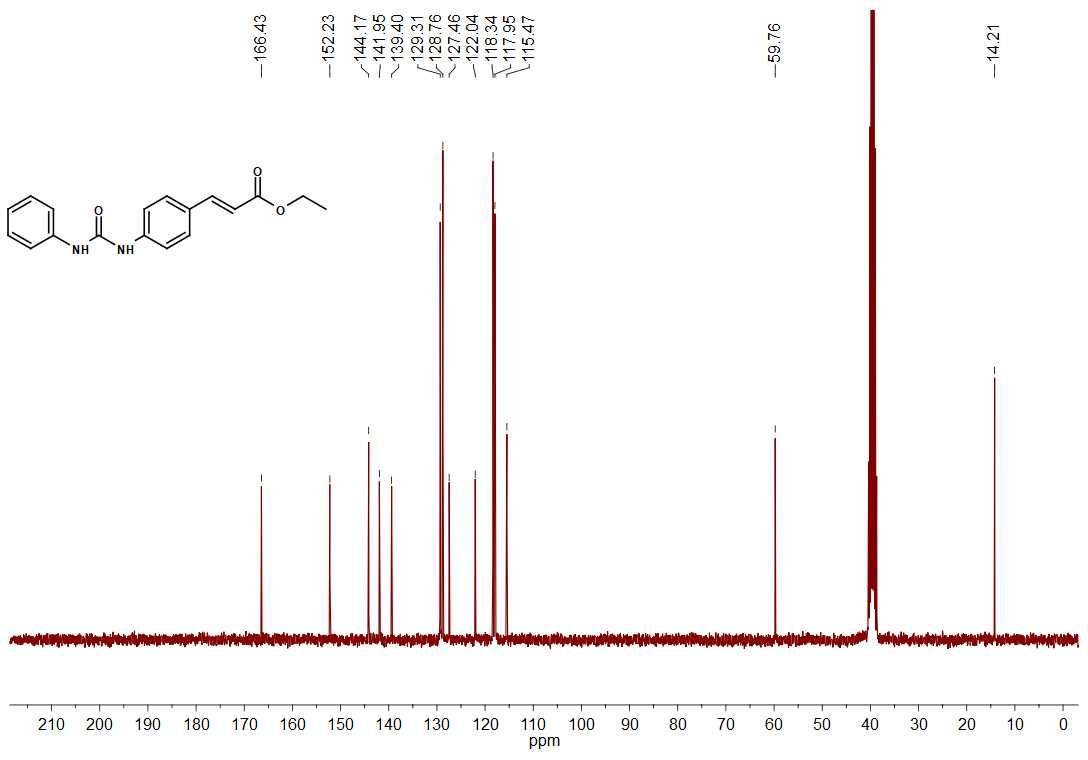


## (*E*)-*N*-hydroxi-3-(4-(3-phenylureido)phenyl)acrylamide (**6a**)


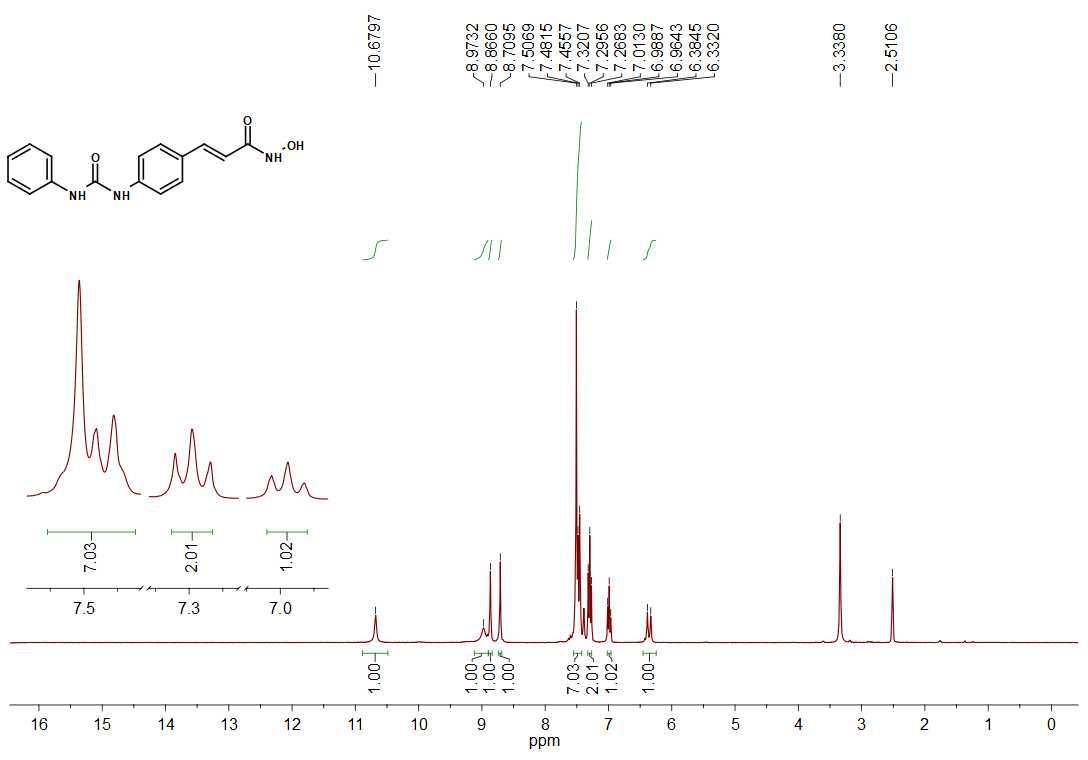


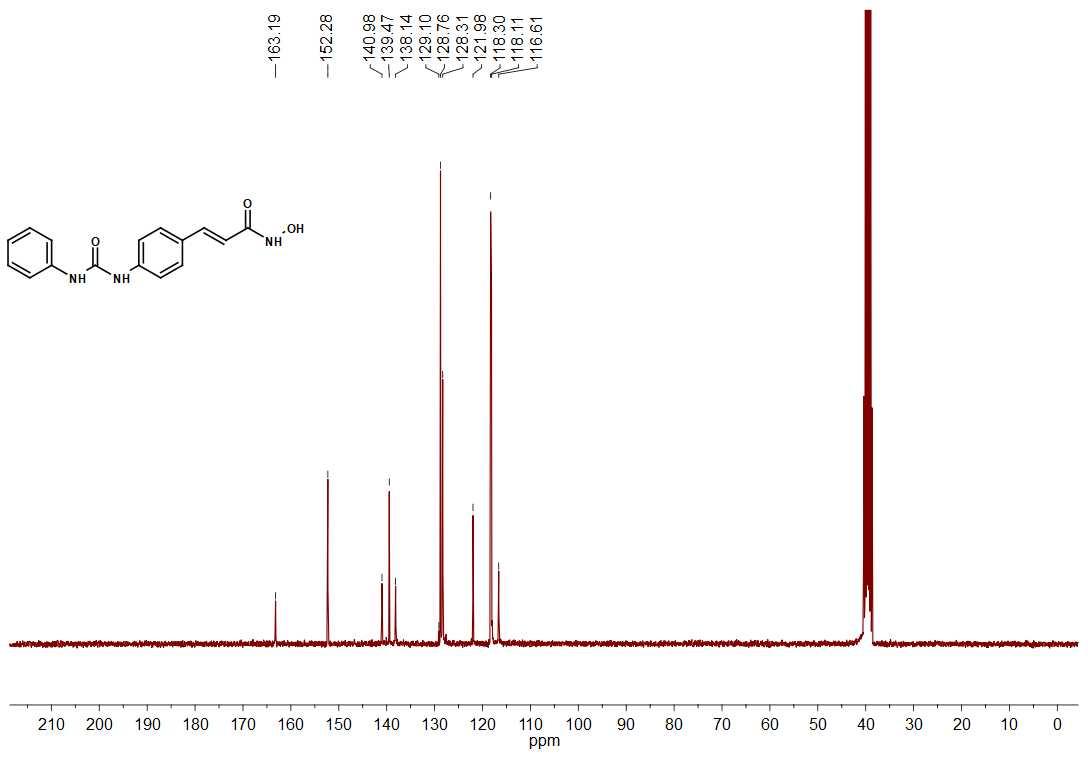


## Ethyl (E)-3-(4-(3-(4-chlorophenyl)ureido)phenyl)acrylate (**10b**)


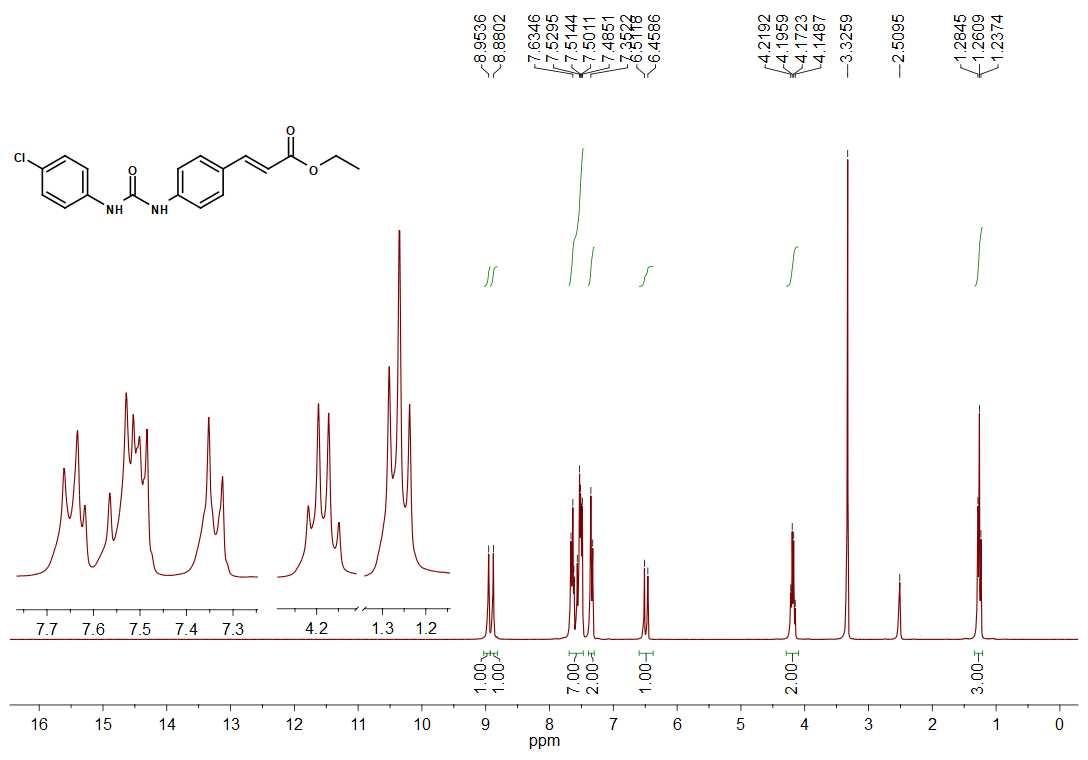


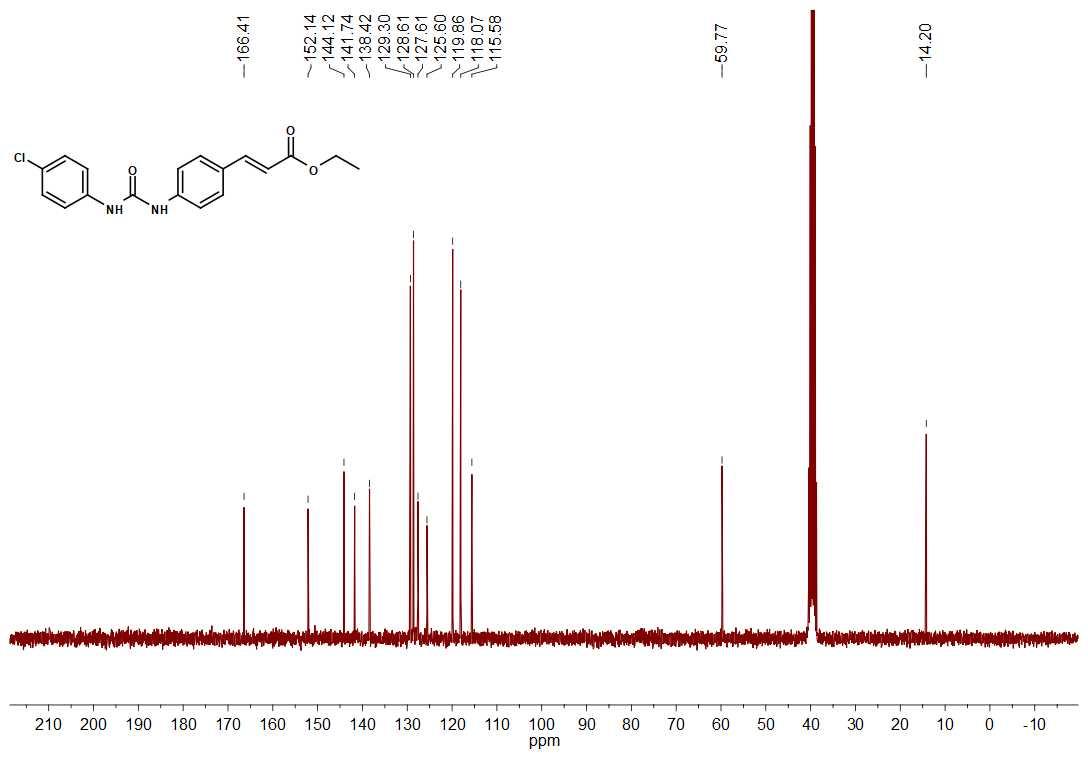


## (*E*)-3-(4-(3-(4-chlorophenyl)ureido)phenyl)-*N*-hydroxiacrylamide (**6b**)


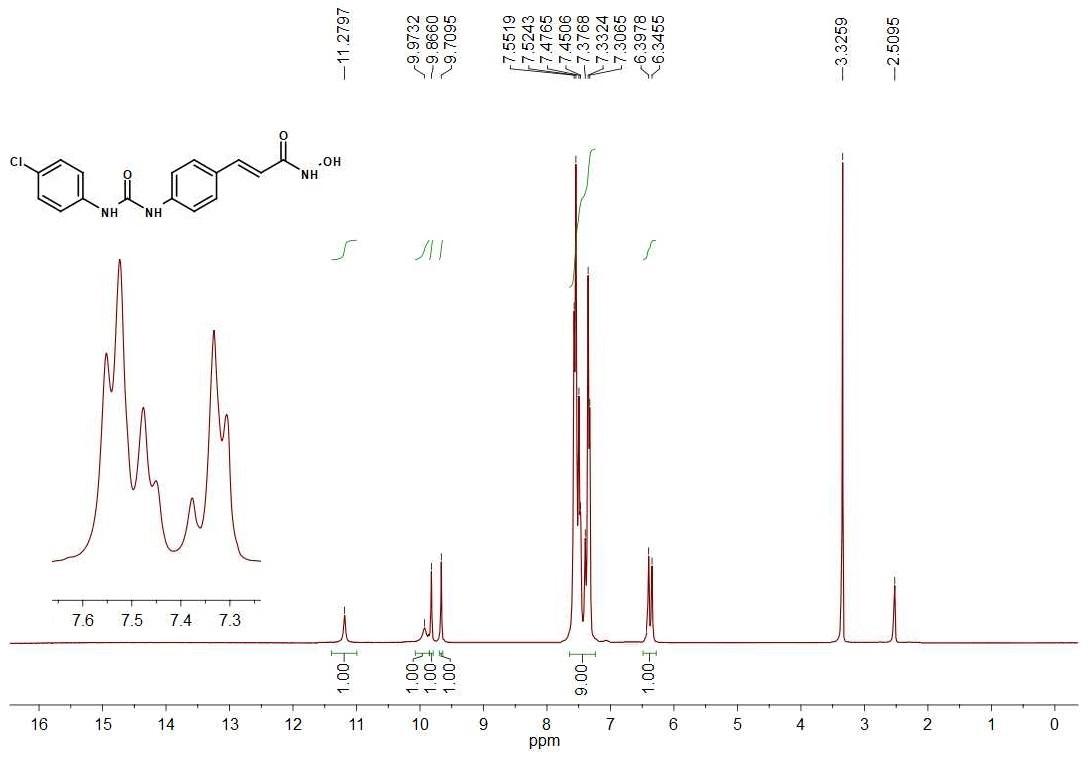


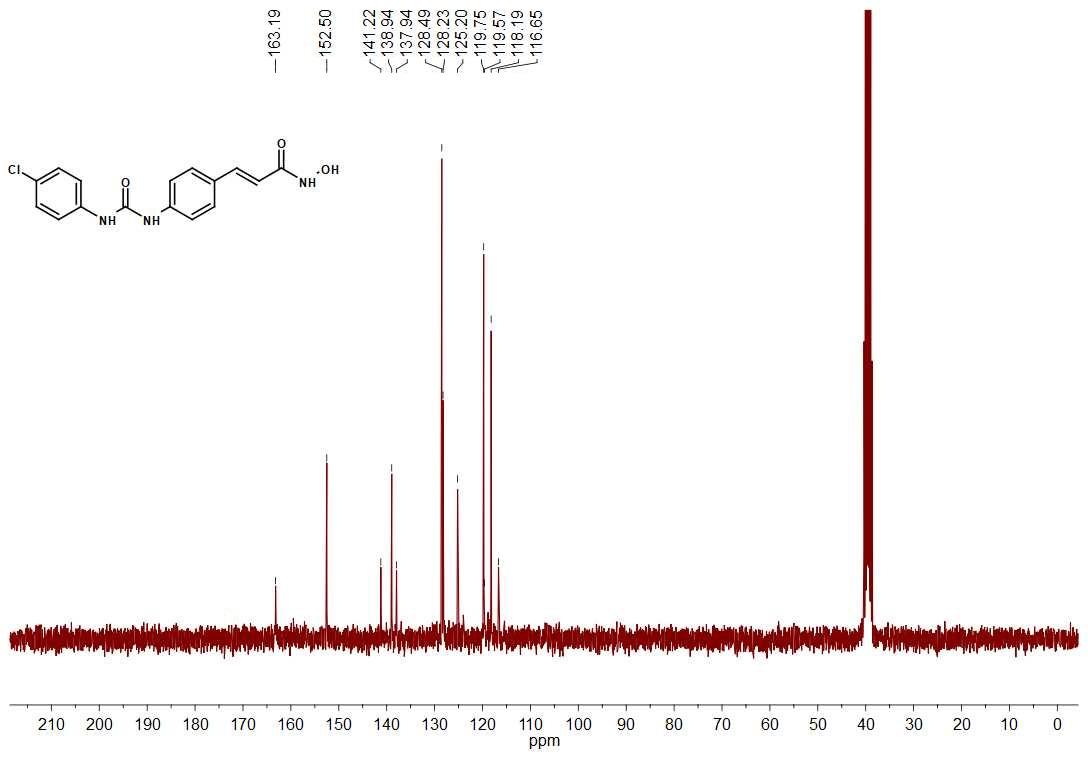


## Ethyl (*E*)-3-(4-(3-(4-methoxyphenyl)ureido)phenyl)acrylate (**10c**)


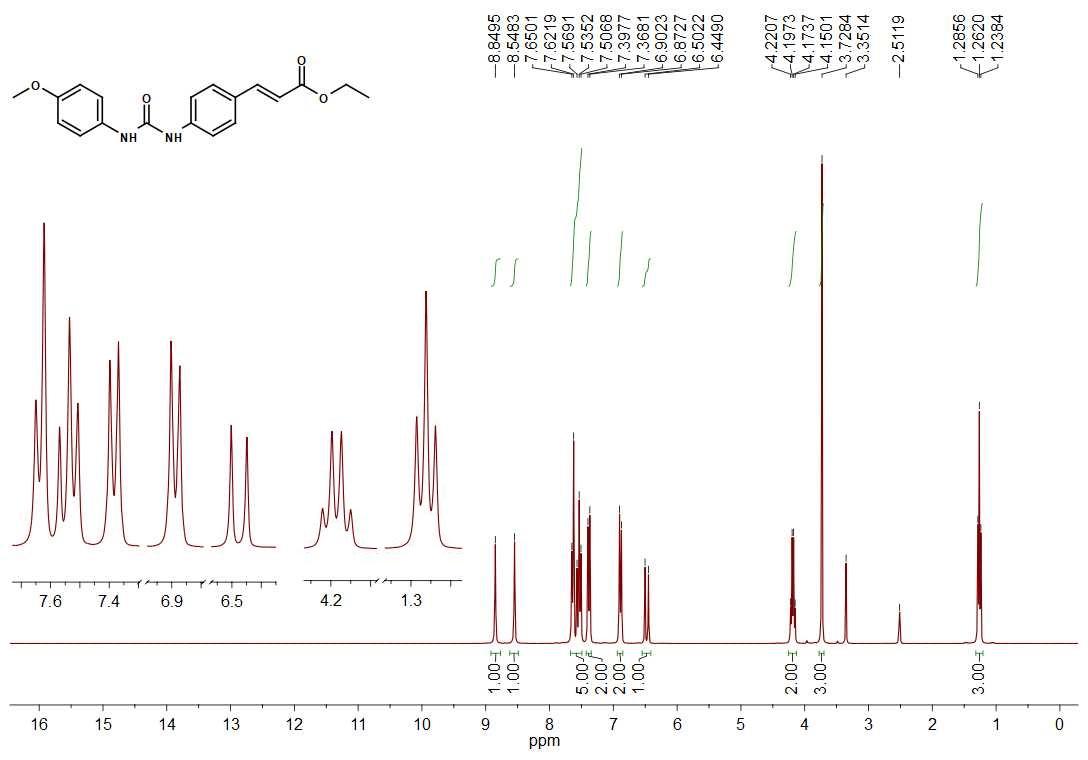


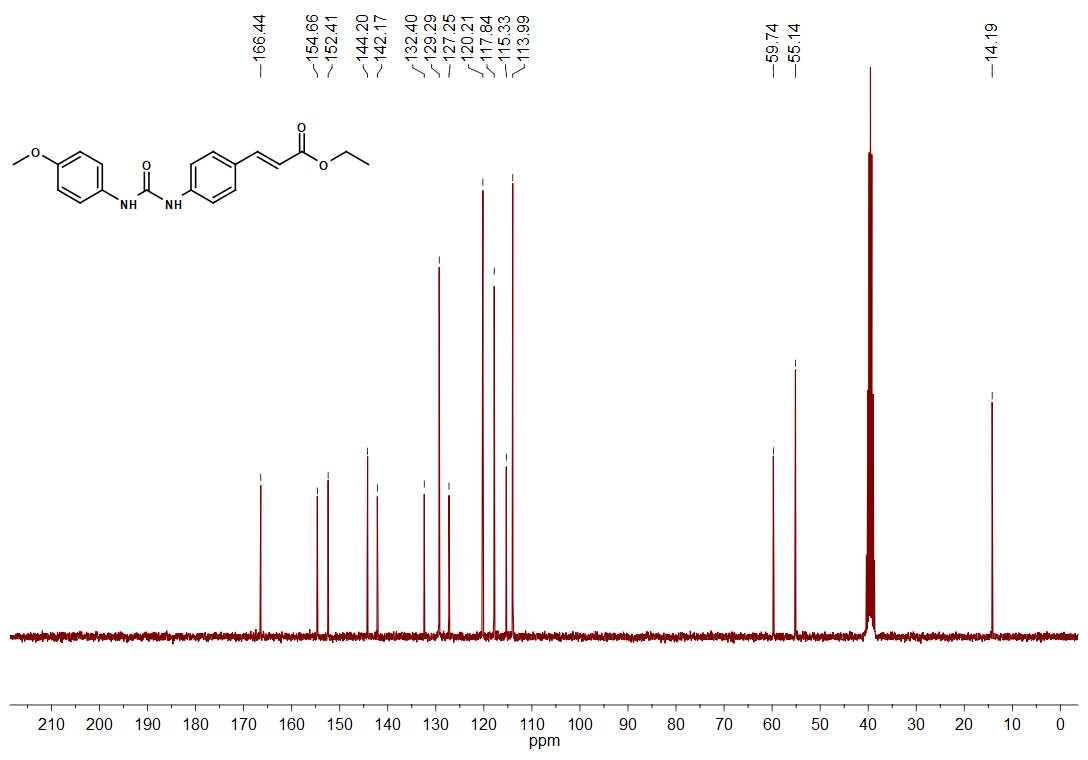


## (*E*)-*N*-hydroxi-3-(4-(3-(4-methoxyphenyl)ureido)phenyl)acrylamide (**6c**)


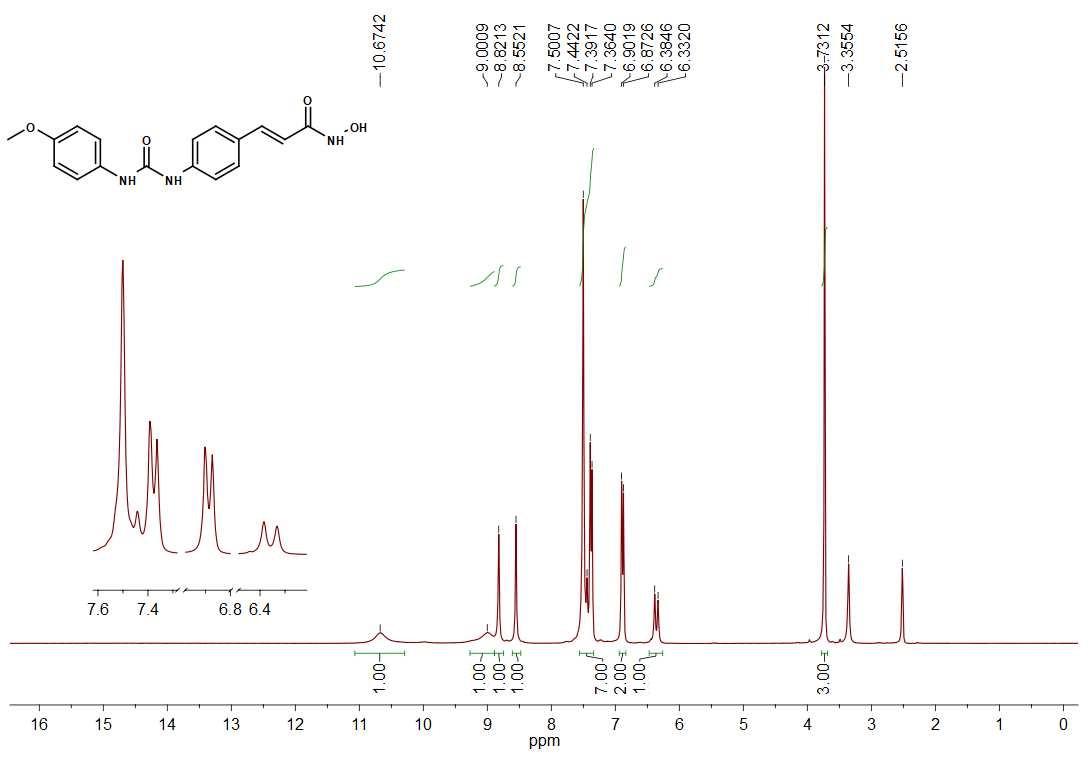


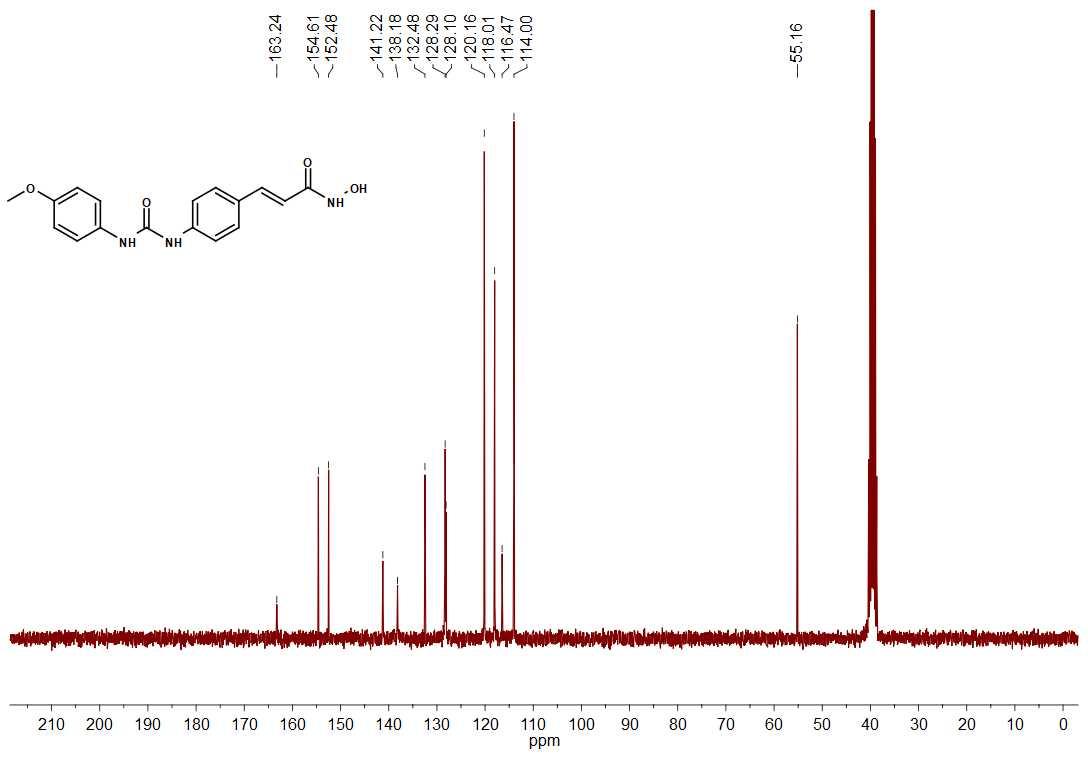


## Ethyl (*E*)-3-(4-(3-(4-nitrophenyl)ureido)phenyl)acrylate (**10d)**


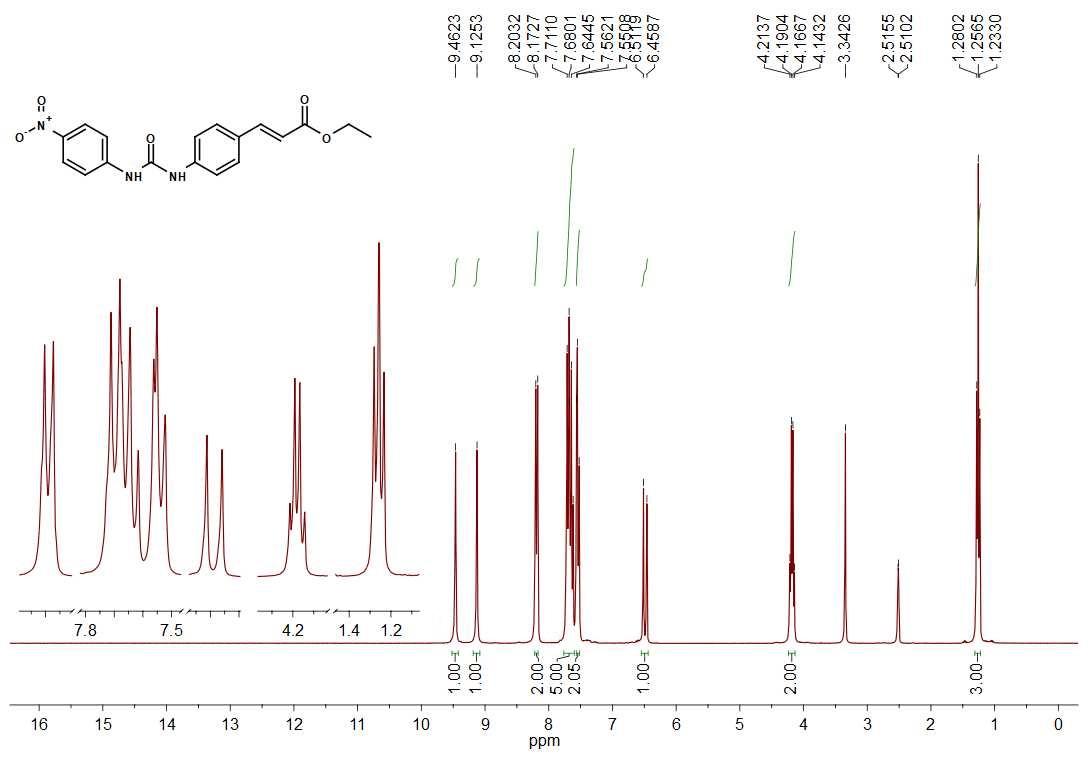


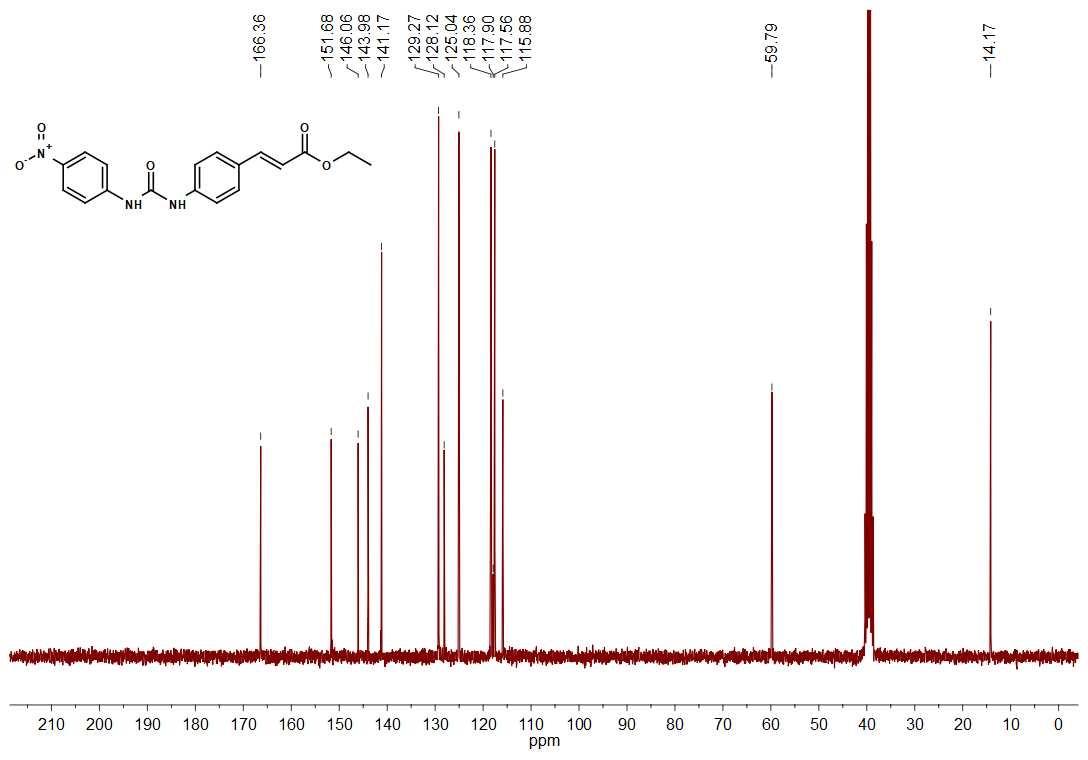


## (*E*)-*N*-hydroxi-3-(4-(3-(4-nitrophenyl)ureido)phenyl)acrylamide (**6d**)


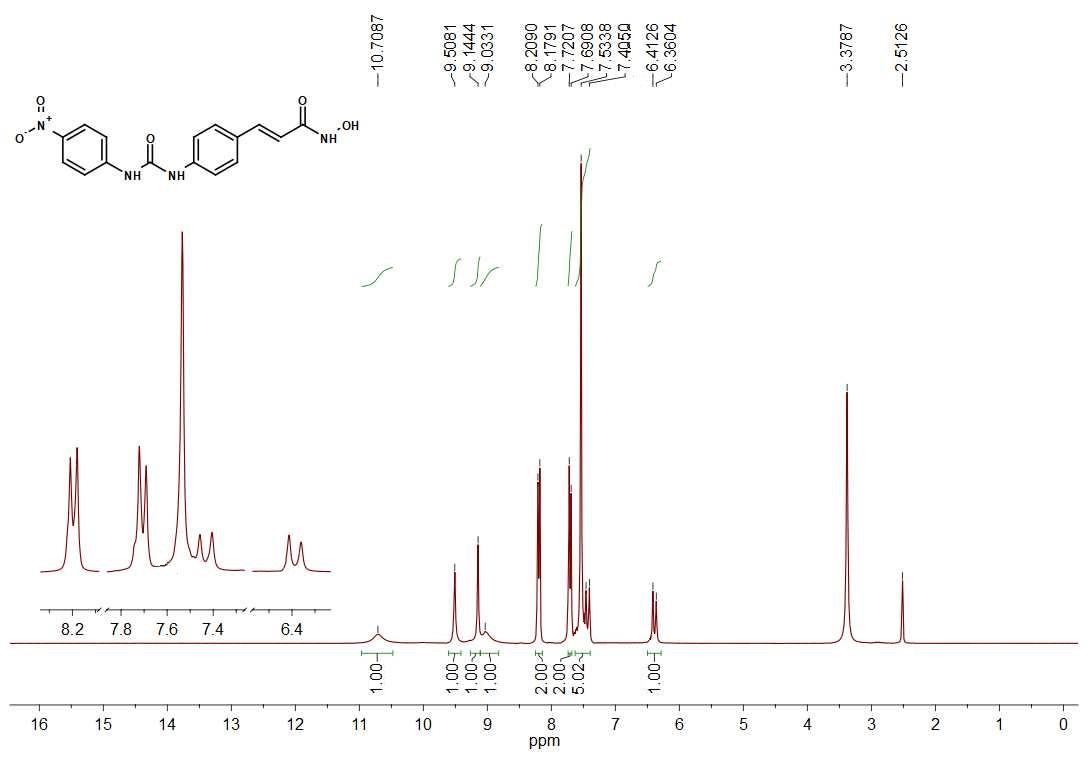


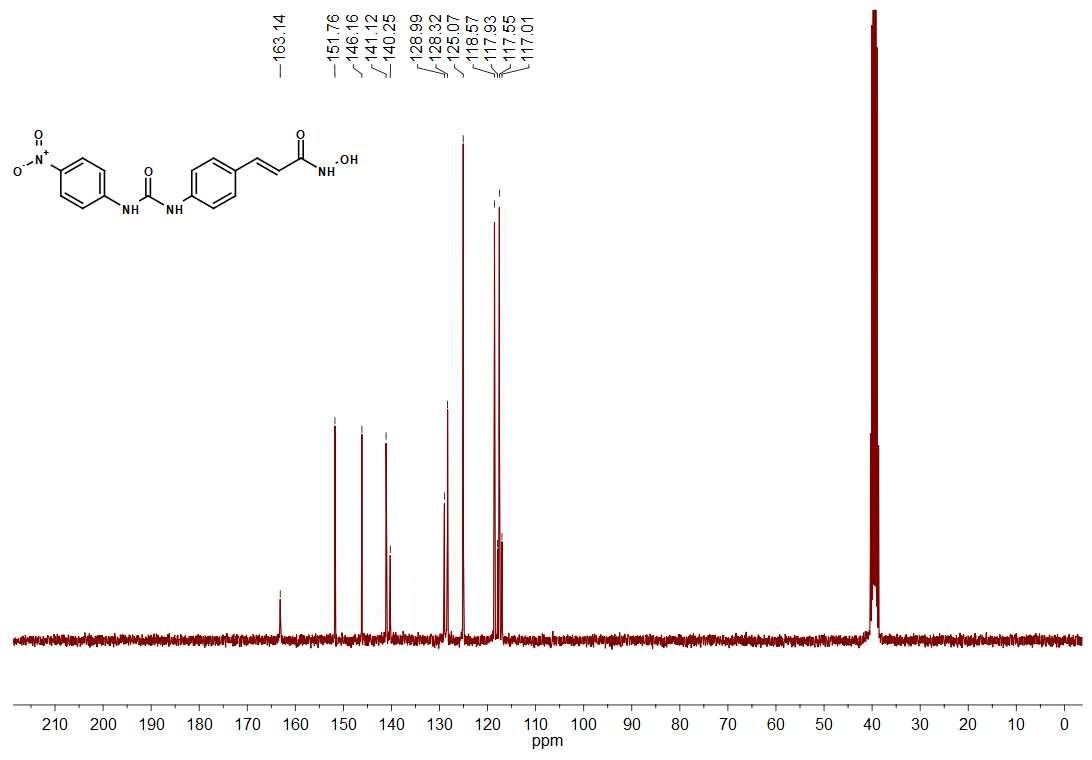


## Ethyl 3-(4-(3-phenylureido)phenyl)propanoate (**11a**)


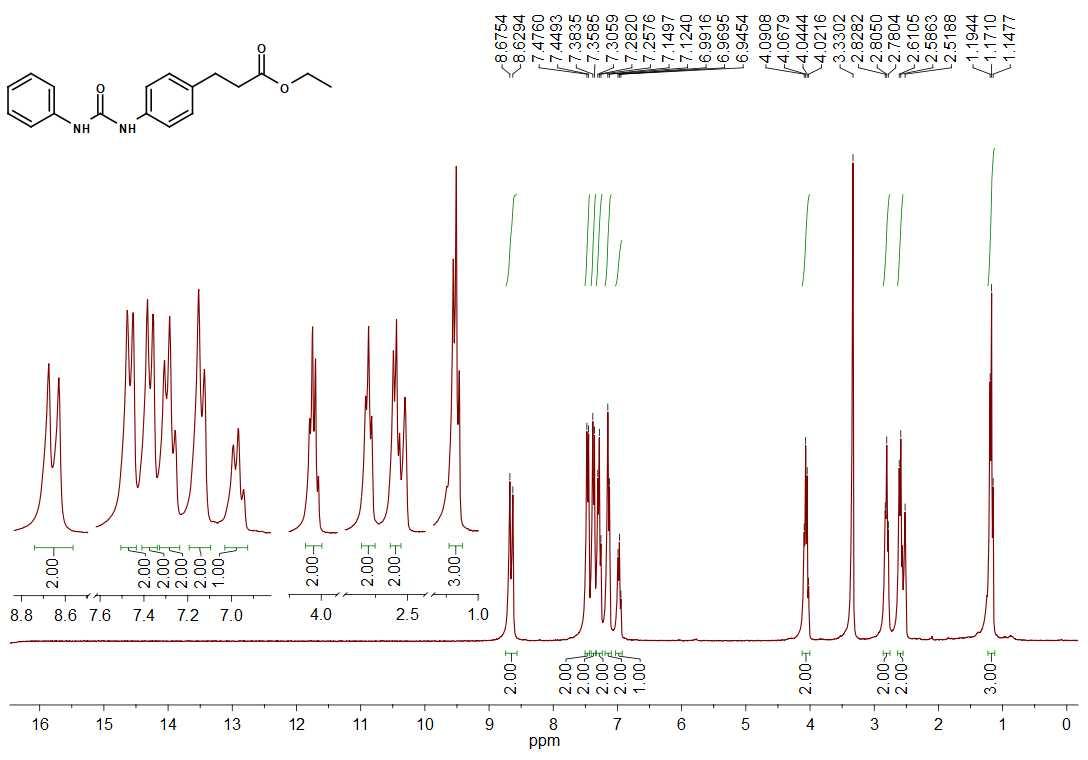


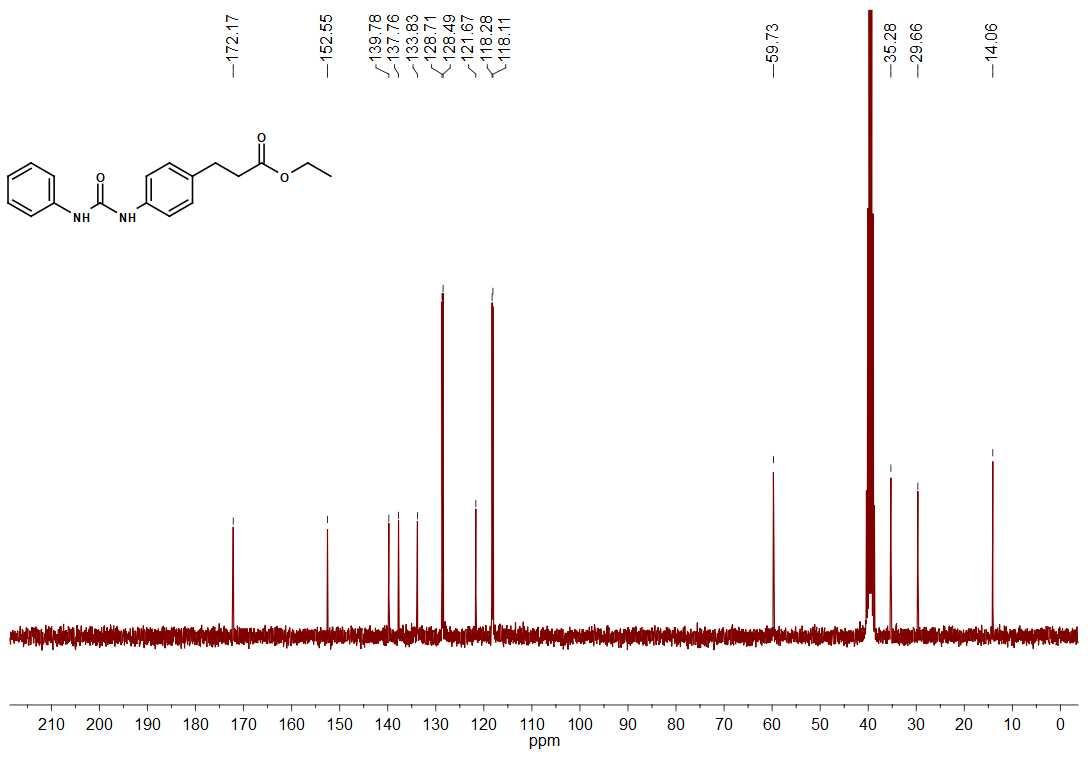


## *N*-hydroxi-3-(4-(3-phenylureido)phenyl)propanamide (**7a**)


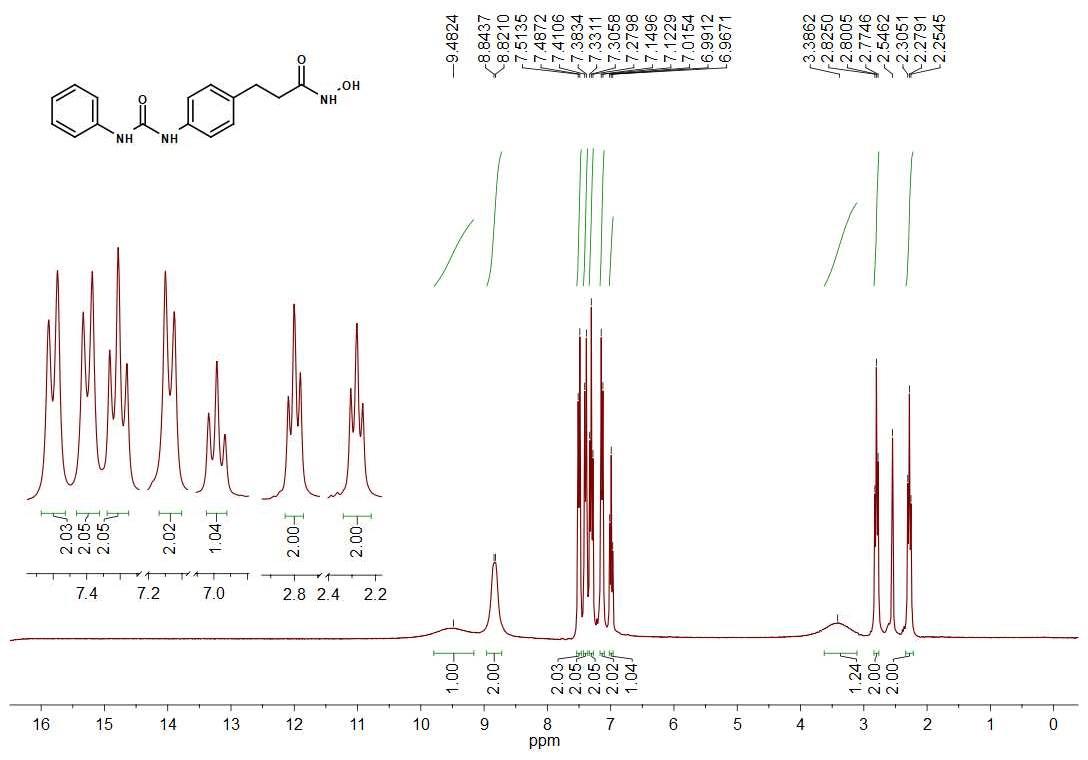


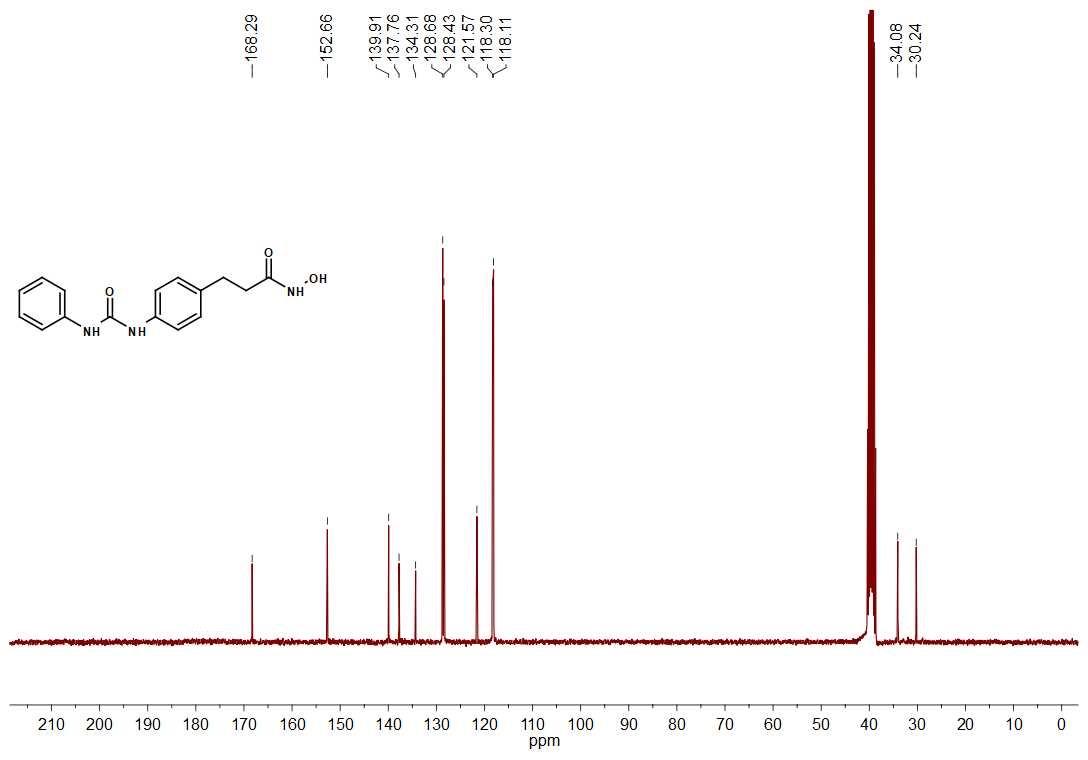


## Ethyl 3-(4-(3-(4-chlorophenyl)ureido)phenyl)propanoate (**11b**)


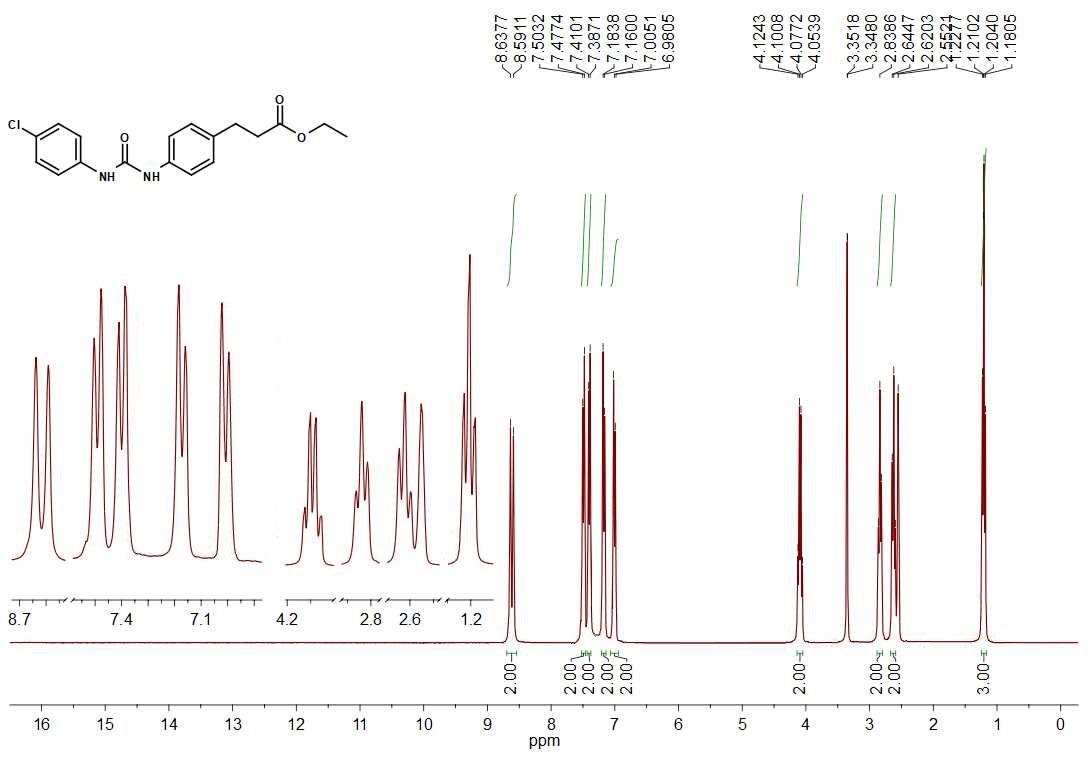


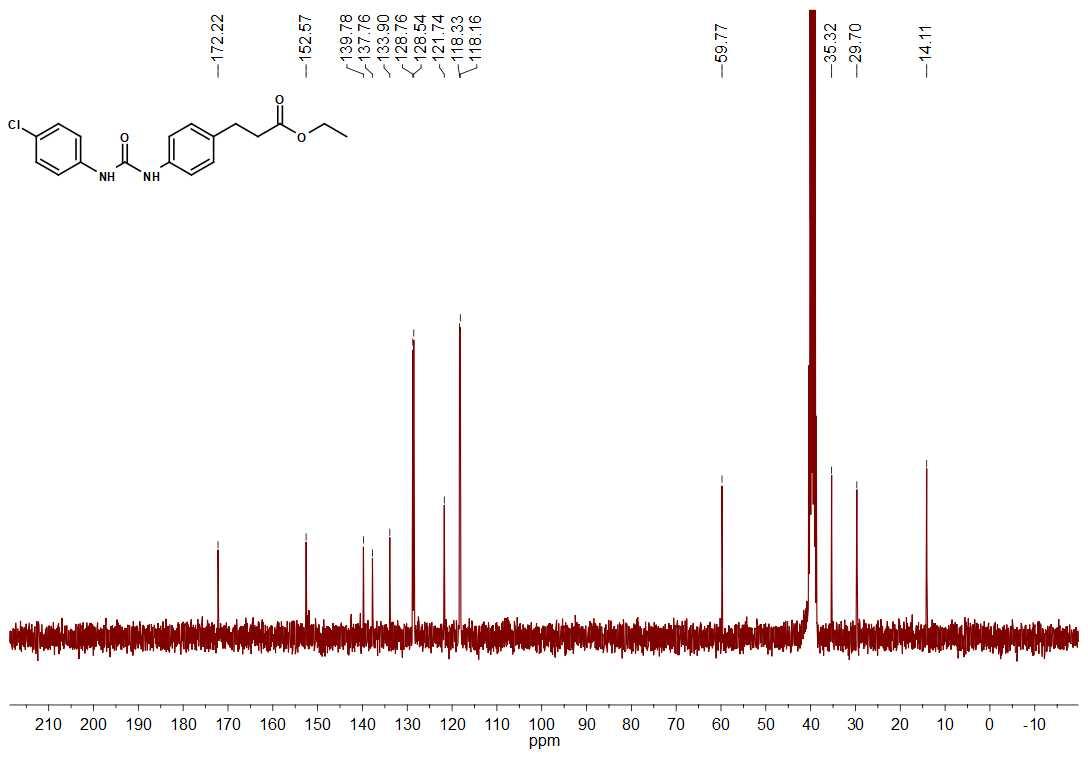


## 3-(4-(3-(4-chlorophenyl)ureido)phenyl)-*N*-hydroxipropanamide (**7b**)


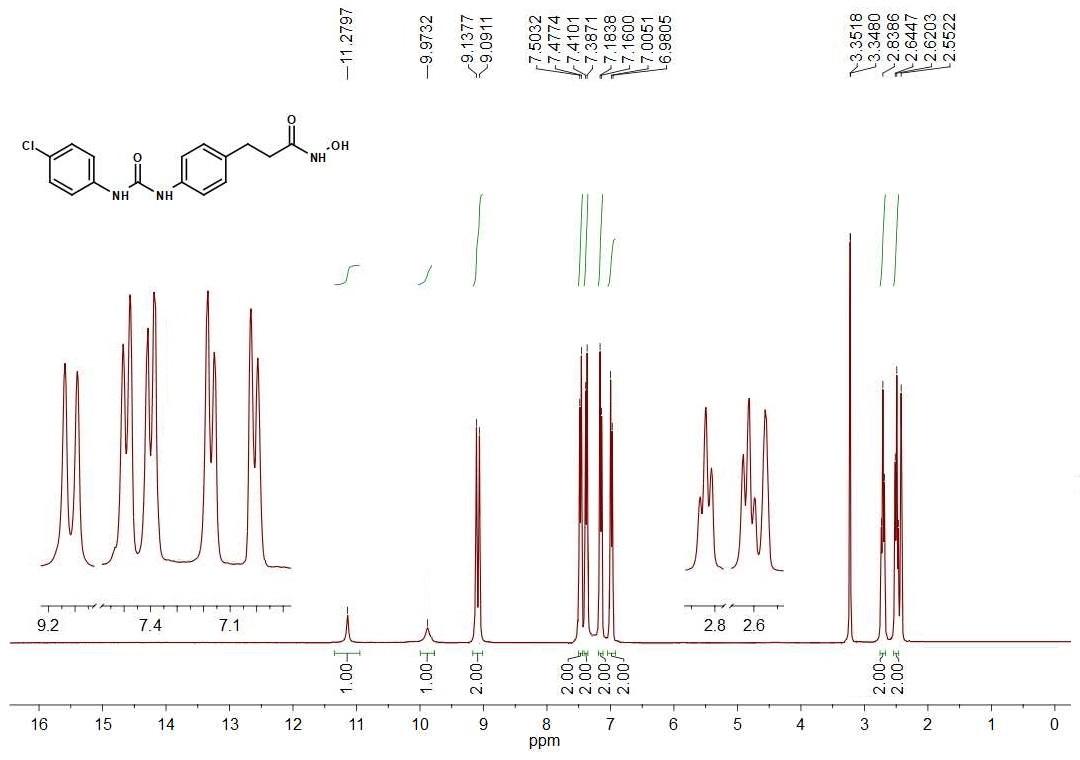


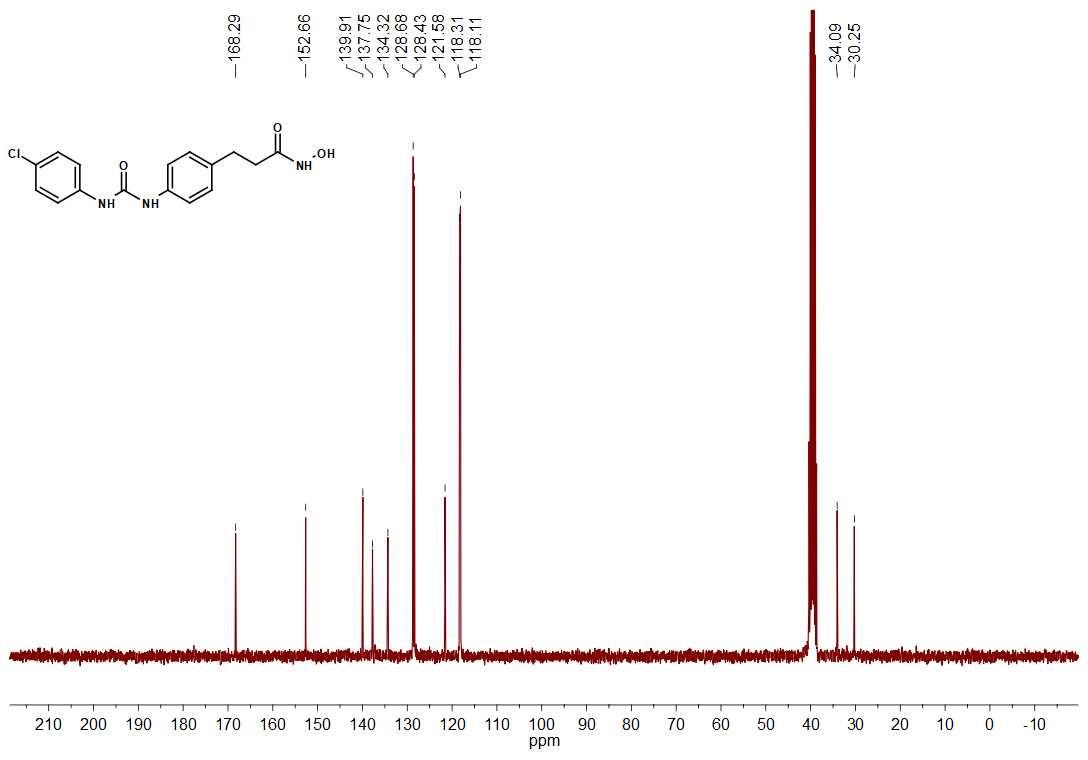


## Ethyl 3-(4-(3-(4-methoxyphenyl)ureido)phenyl)propanoate (**11c**)


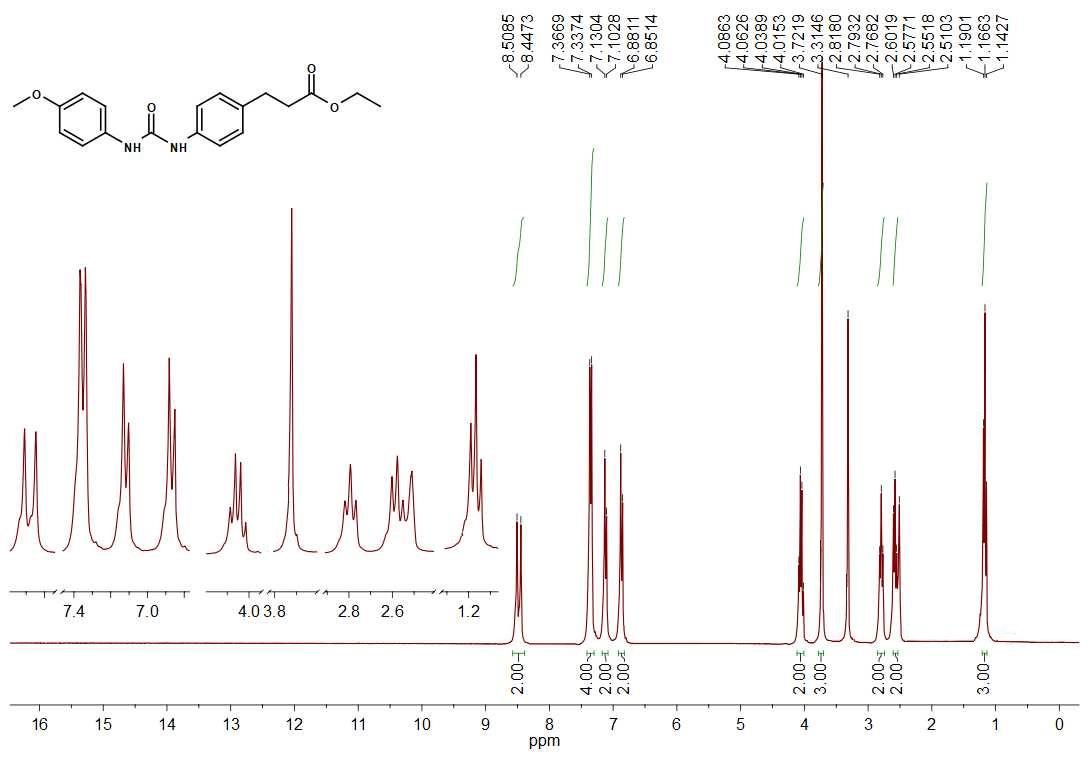


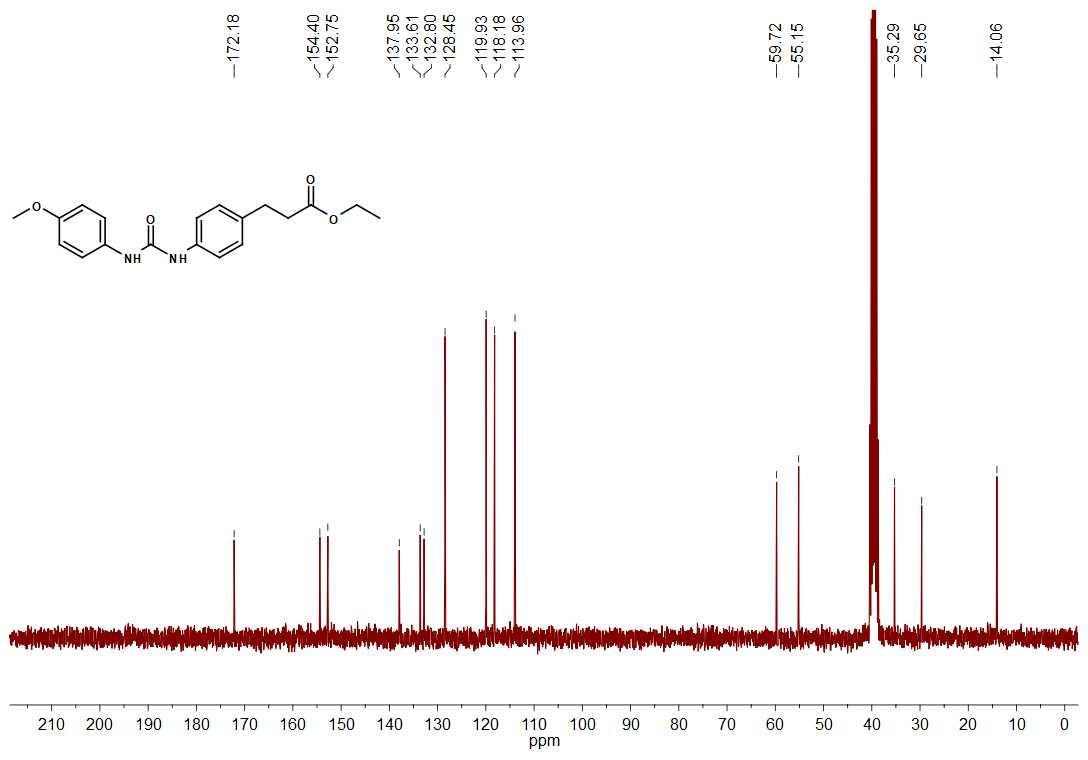


## *N*-hydroxi-3-(4-(3-(4-methoxyphenyl)ureido)phenyl)propanamide (**7c**)


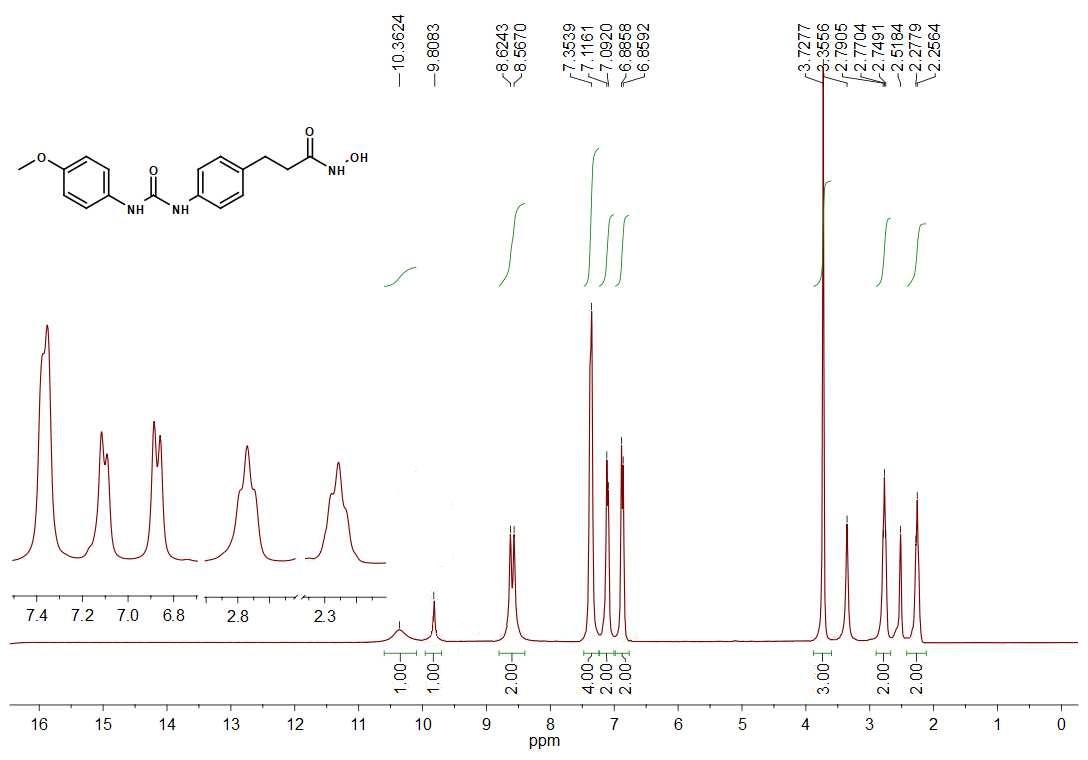


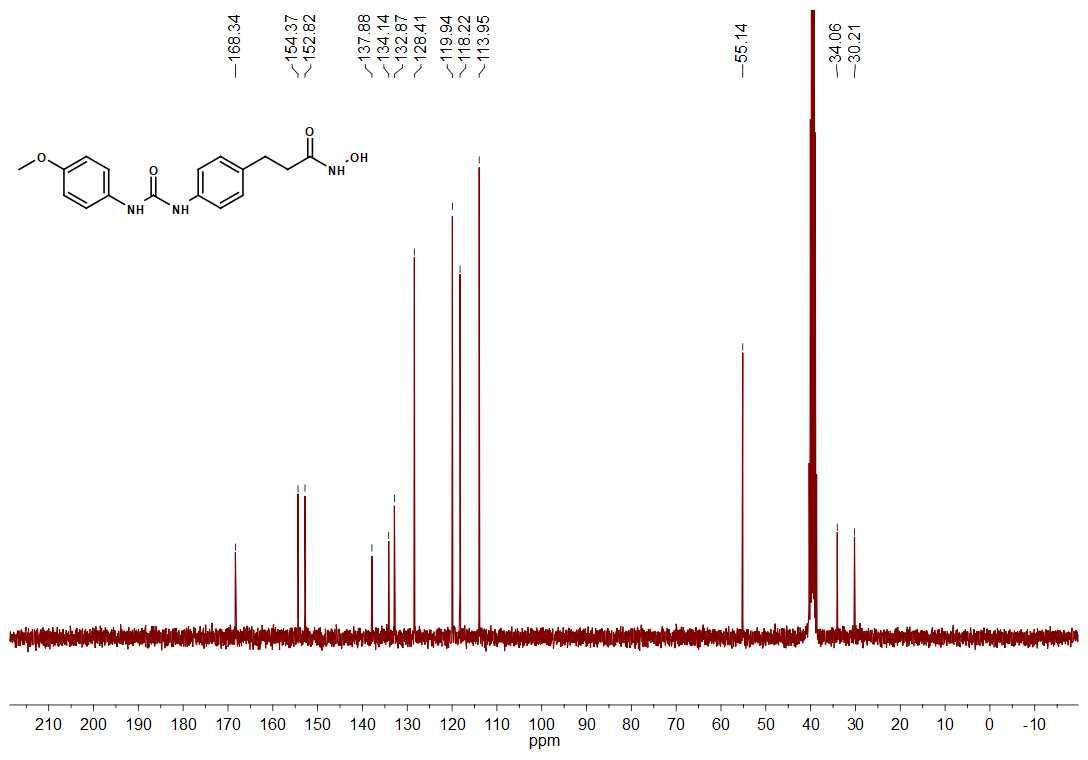


## Methyl 4-(3-phenylureido)benzoate (**13a**)


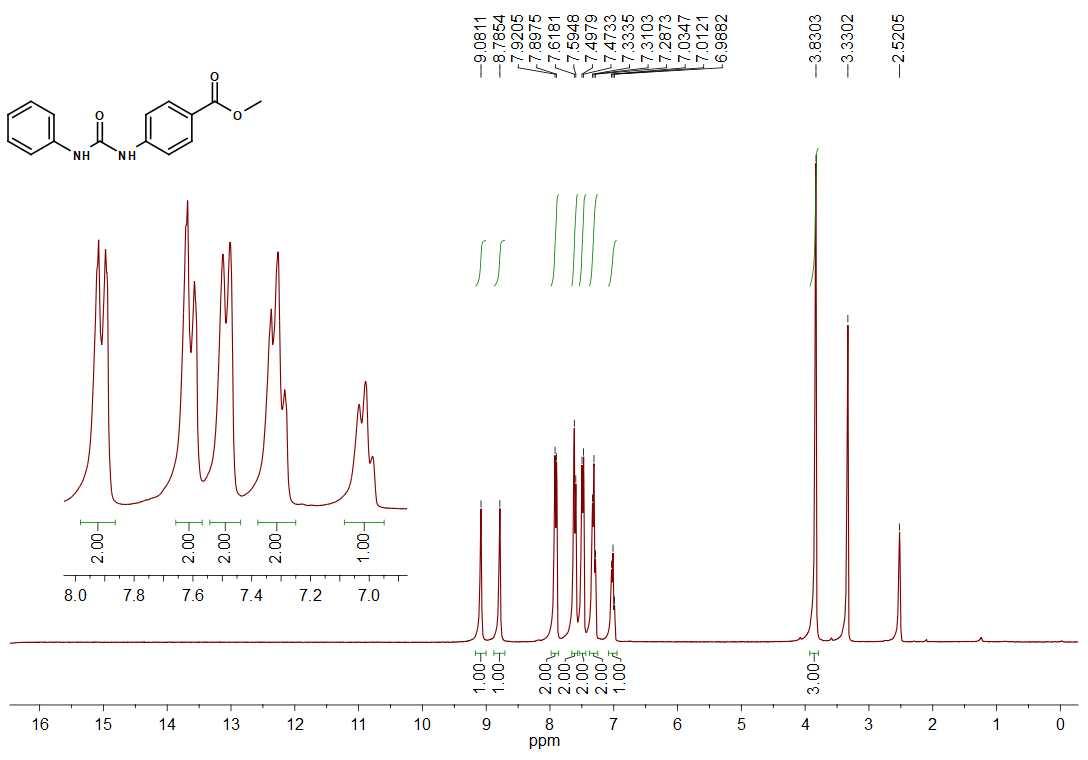


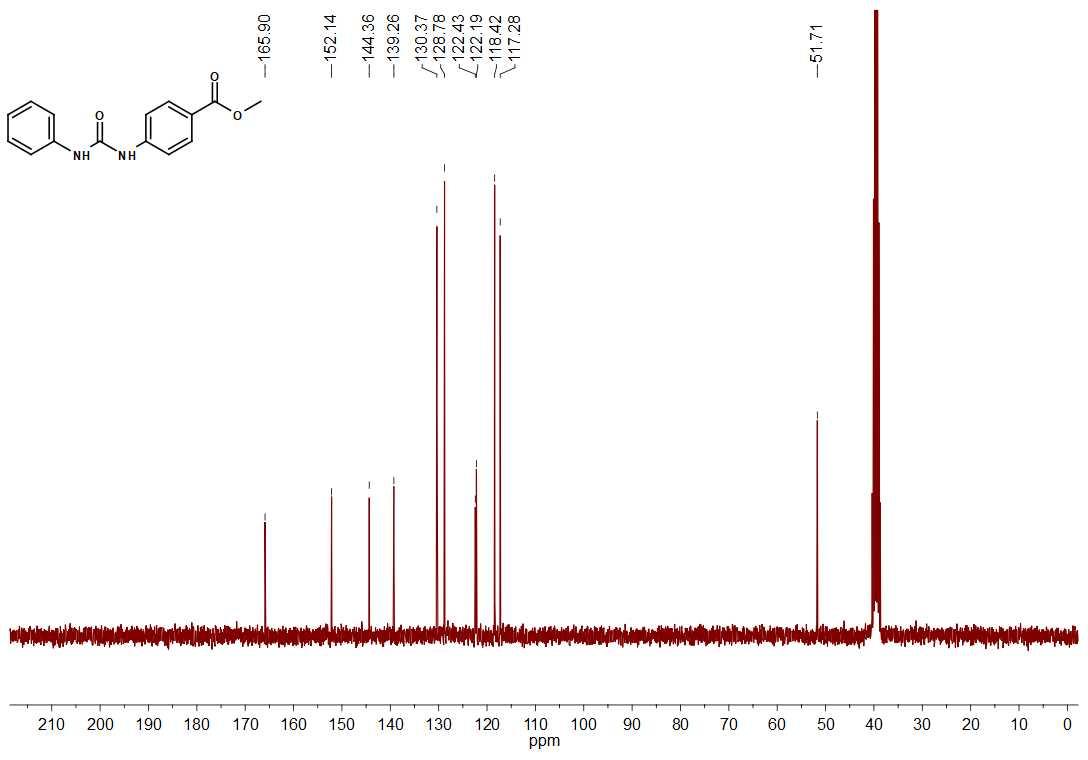


## *N*-hydroxi-4-(3-phenylureido)bezamide (**8a**)


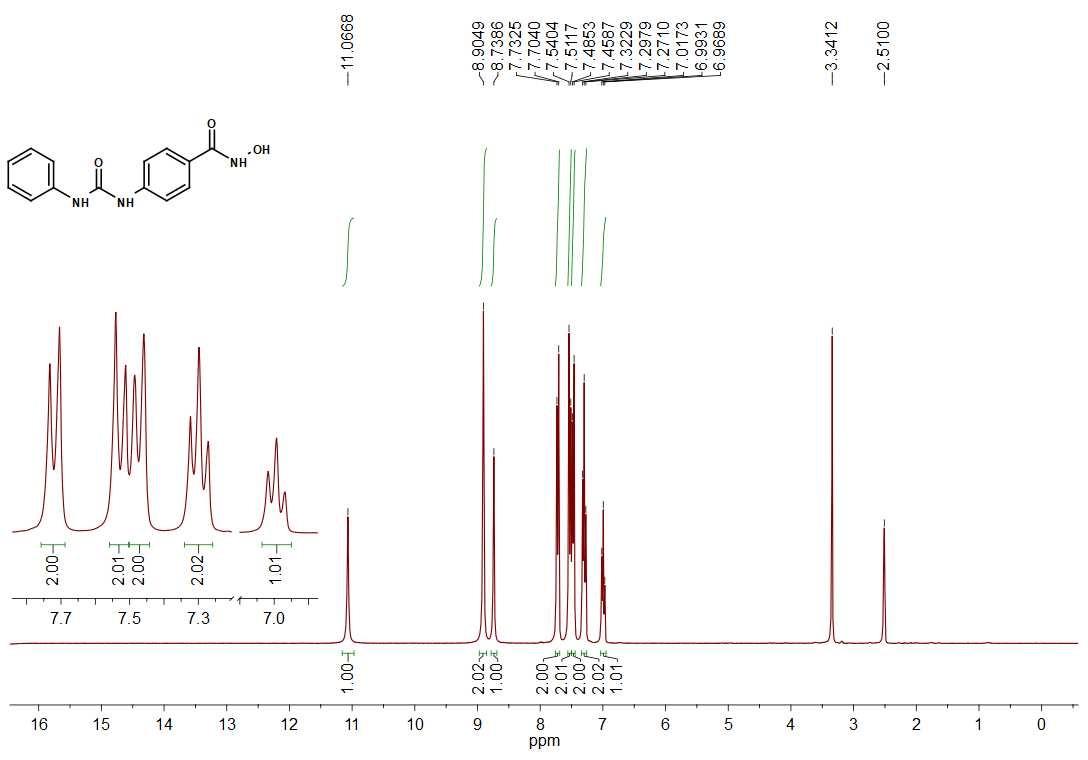


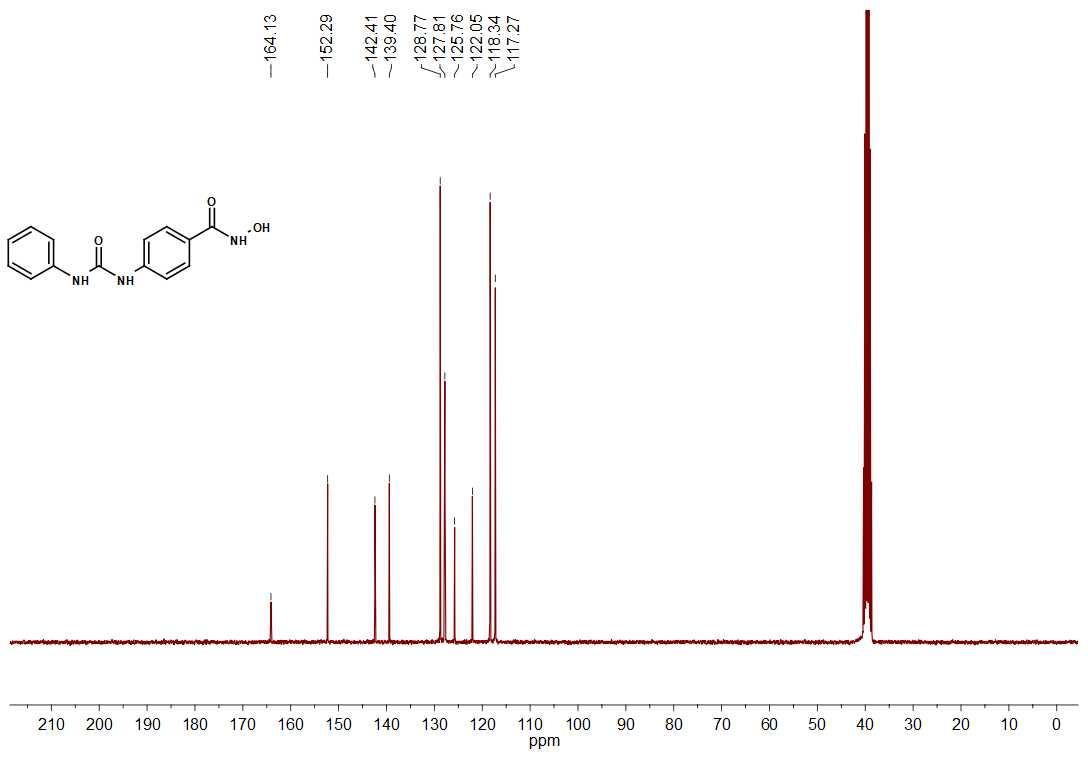


## Methyl 4-(3-(4-chlorophenyl)ureido)benzoate (**13b**)


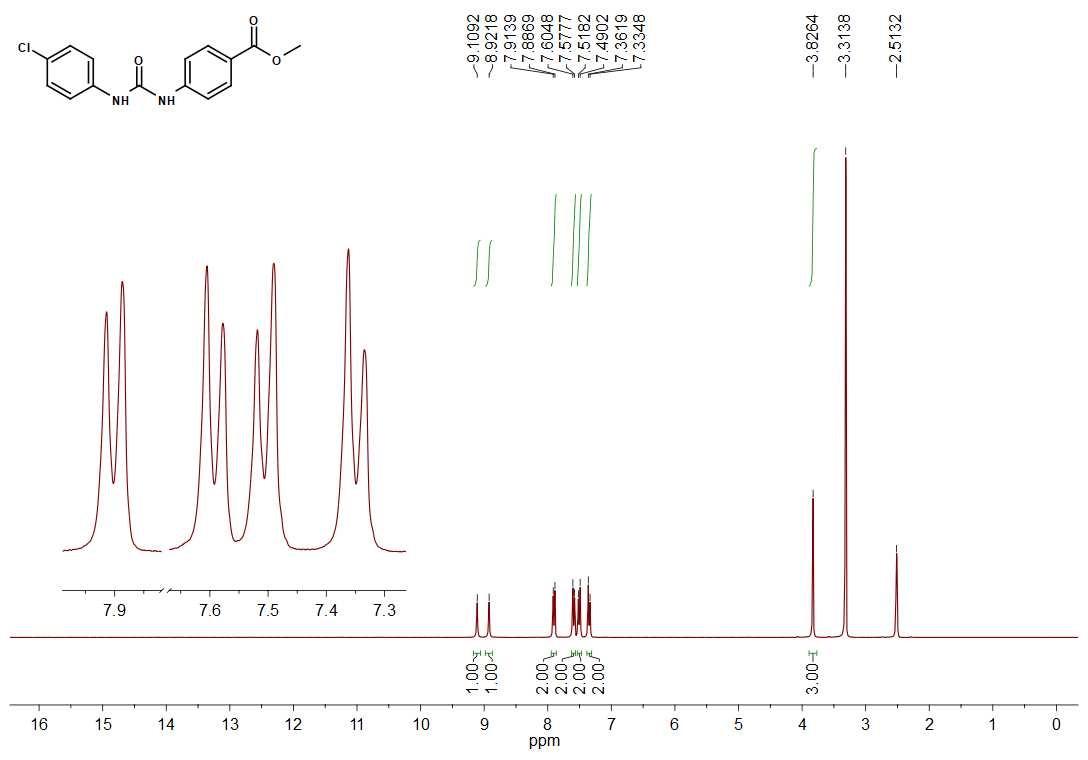


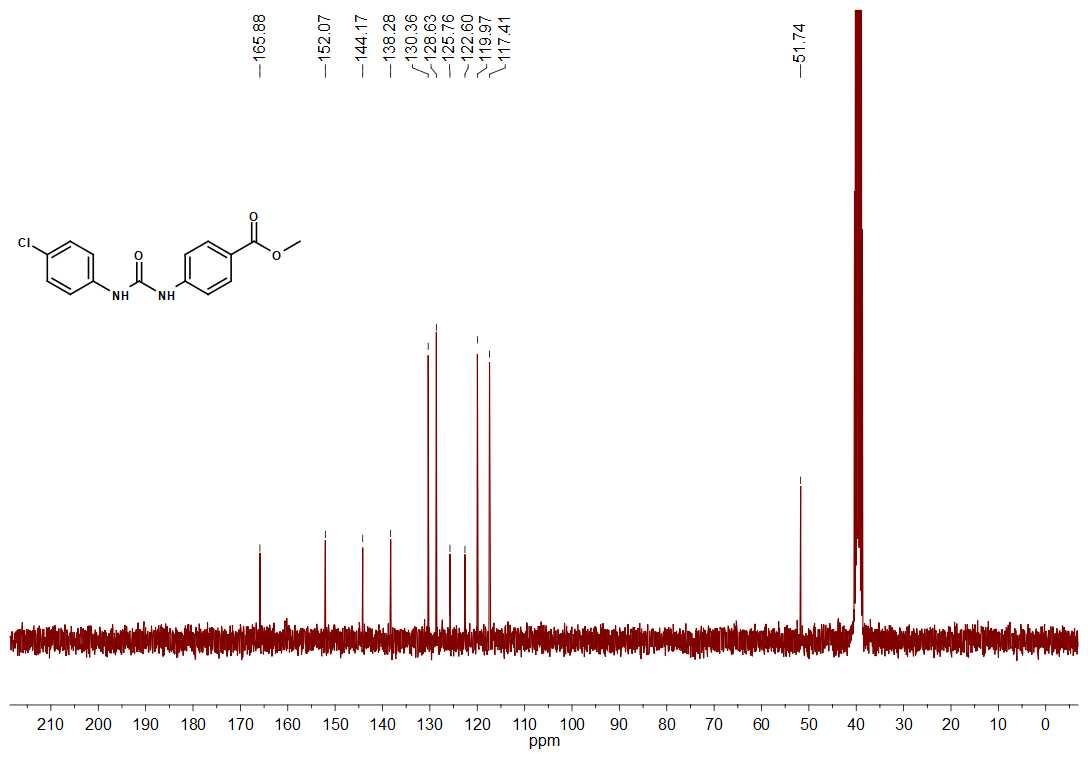


## 4-(3-(4-chlorophenyl)ureido)-*N*-hydroxibenzamide (**8b**)


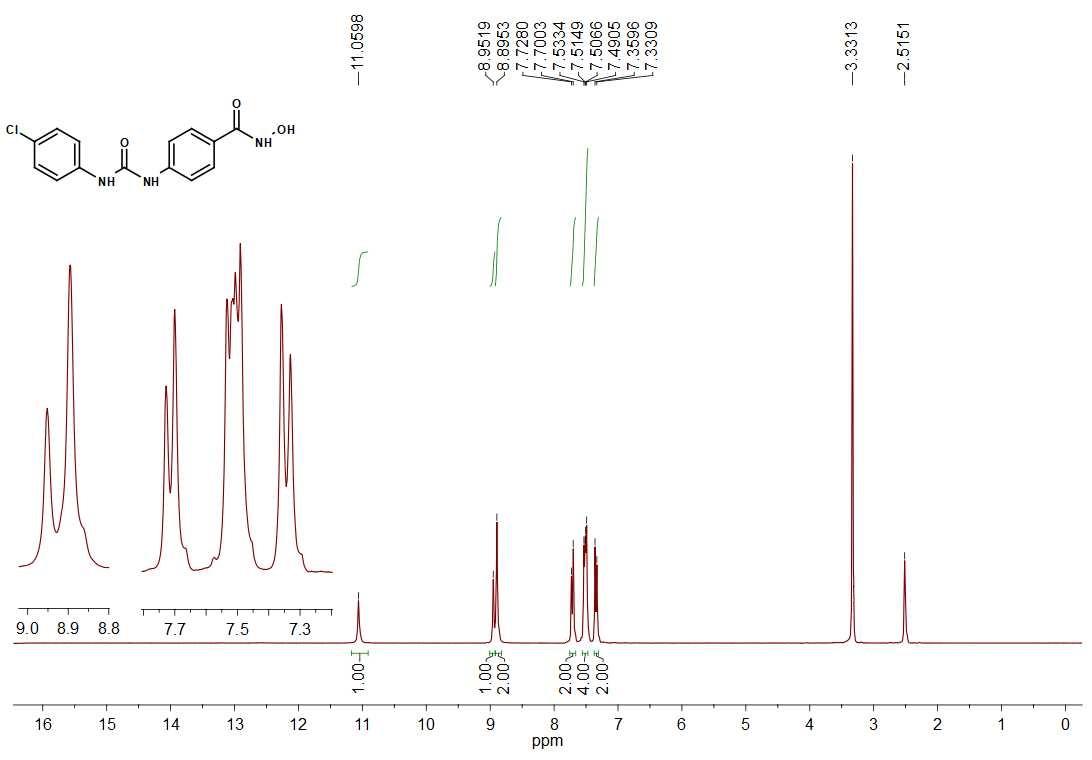


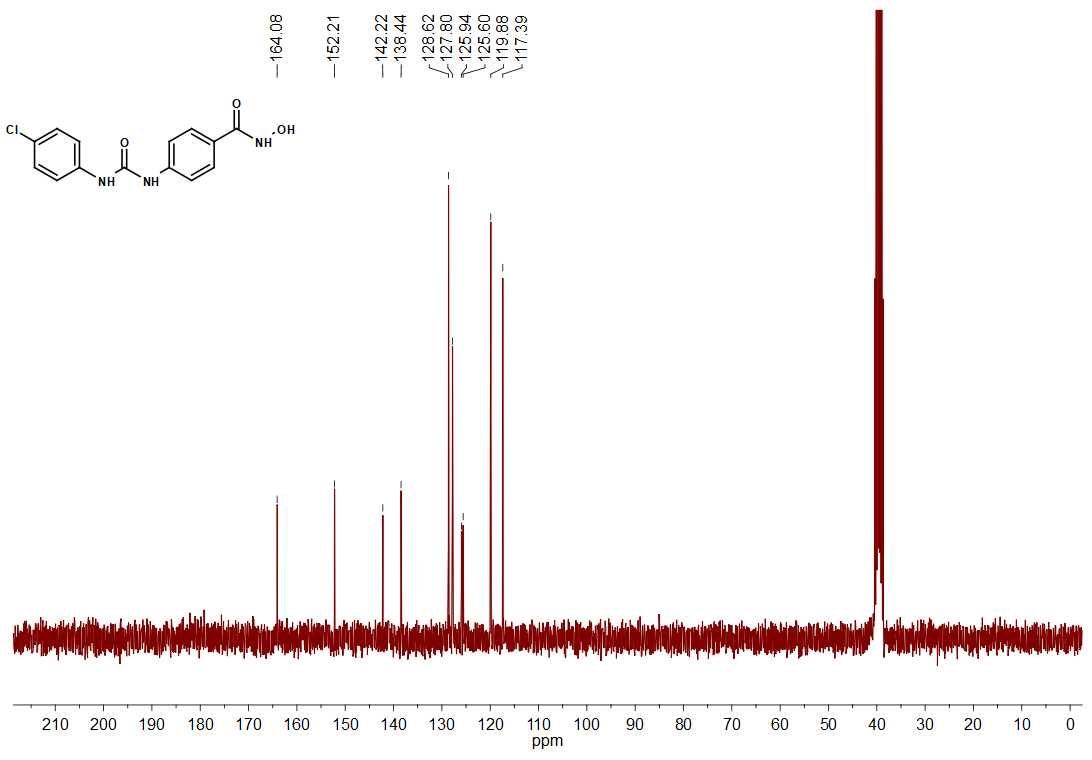


## Methyl 4-(3-(4-methoxyphenyl)ureido)benzoate (**13c**)


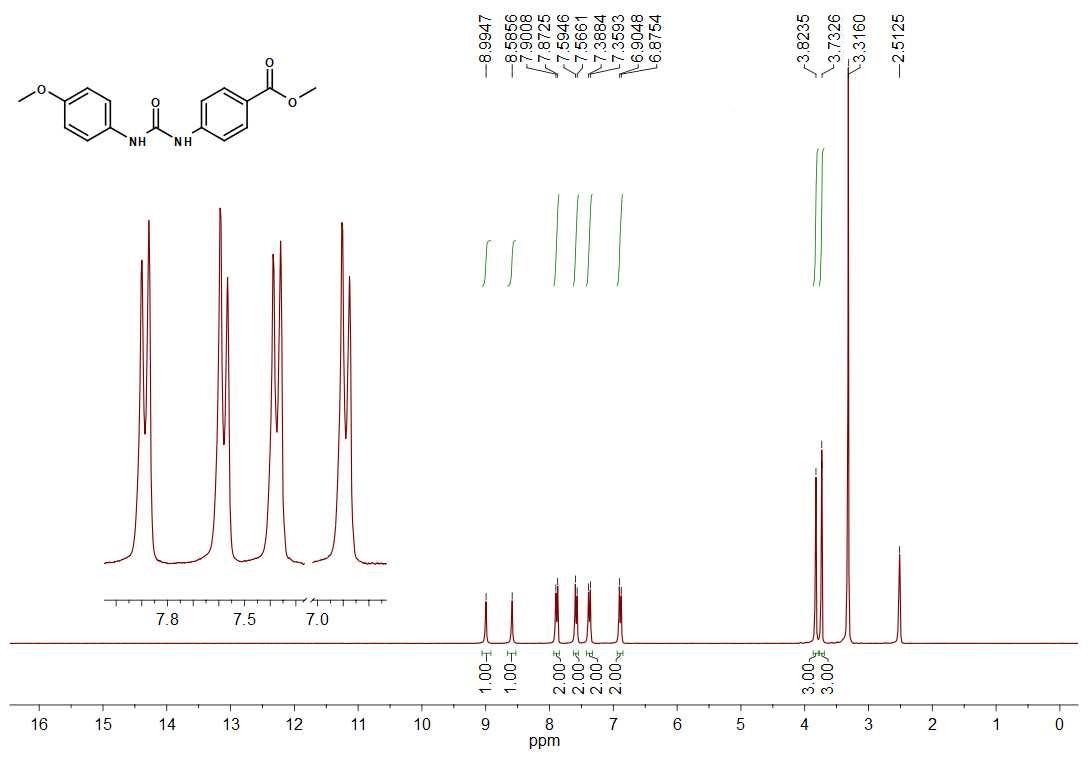


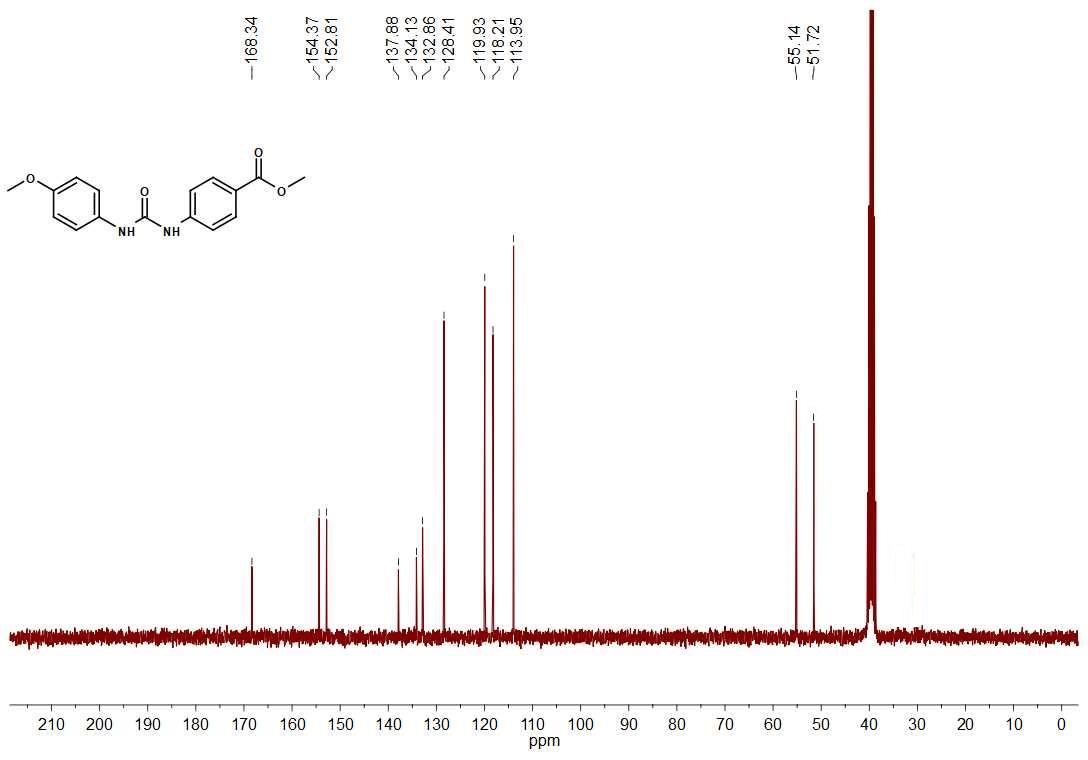


## *N*-hydroxi-4-(3-4(-methoxyphenyl)ureido)benzamide (**8c**)


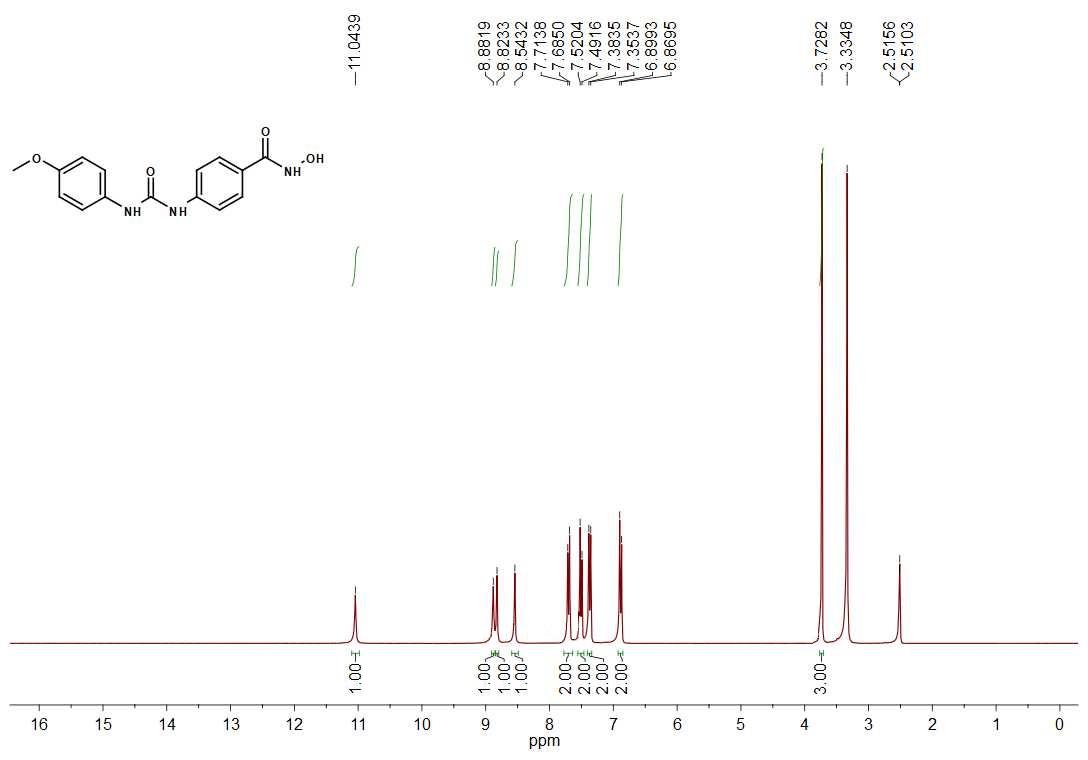


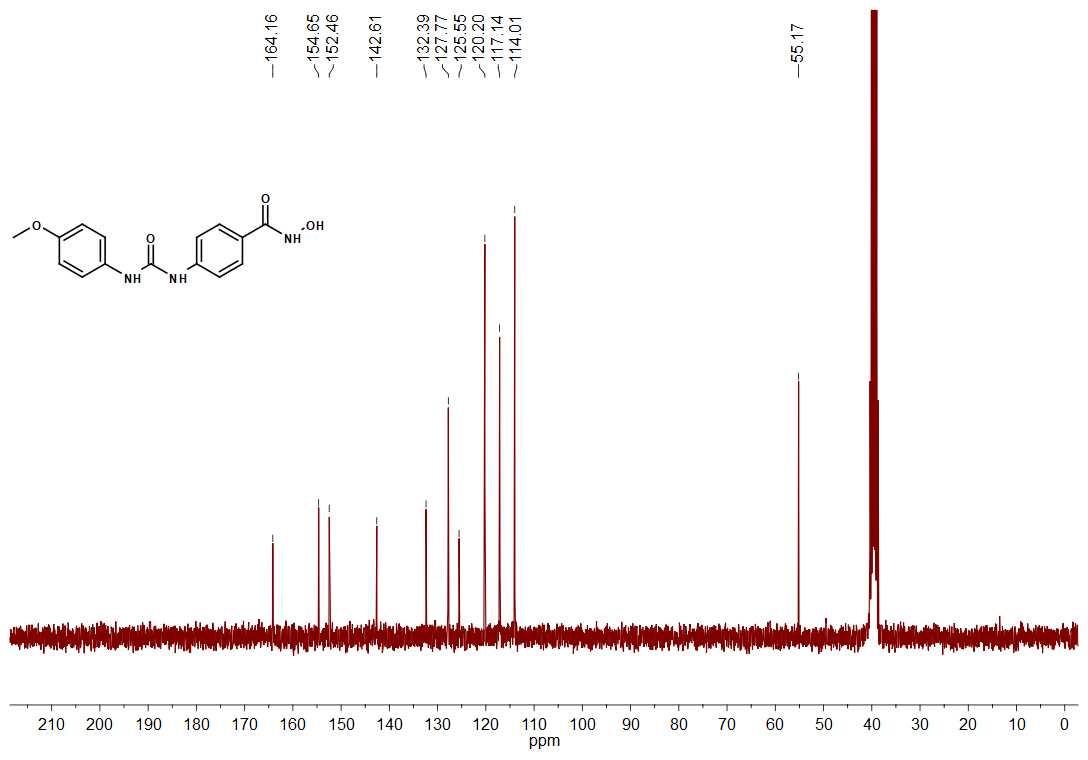


## Methyl 4-(3-(4-nitrophenyl)ureido)benzoate (**13d**)


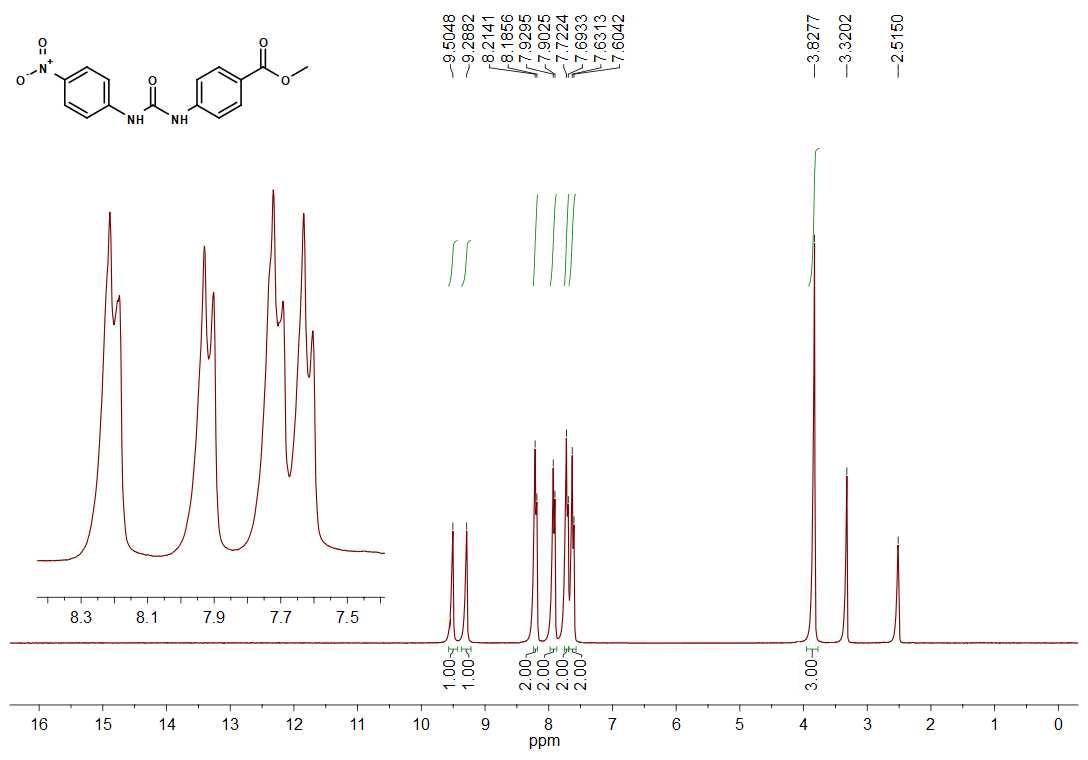


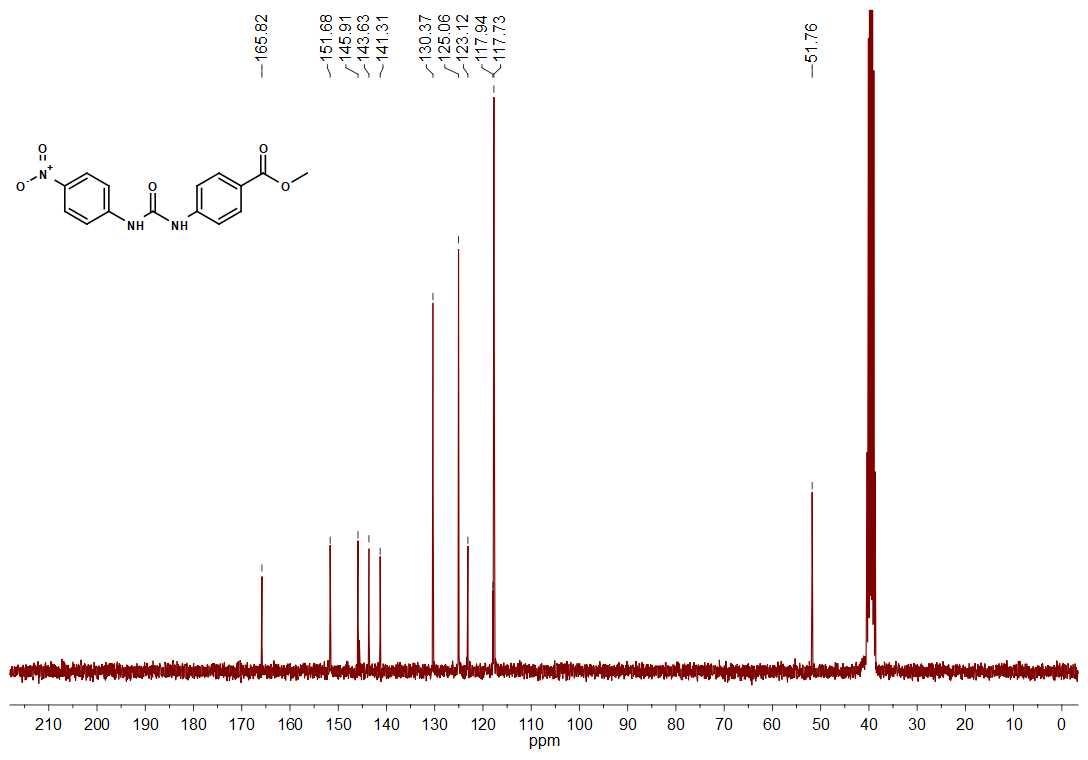


## *N-*hydroxi-4-(3-(4-nitrophenyl)ureido)benzamide (**8d**)


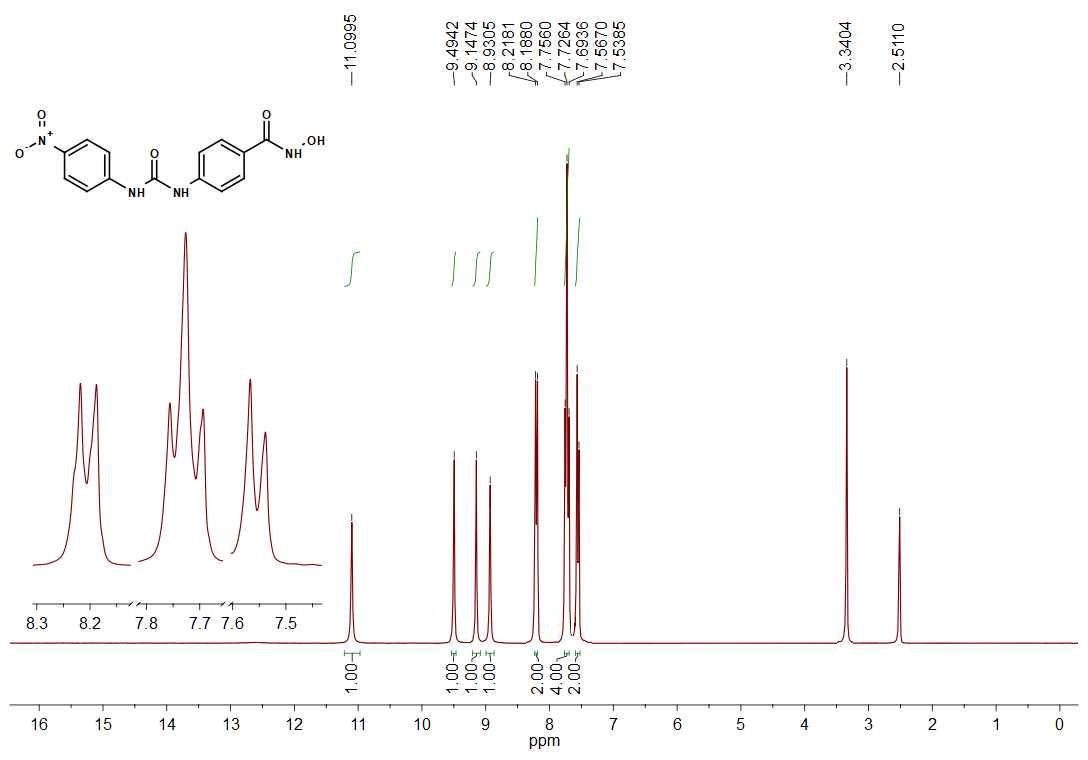


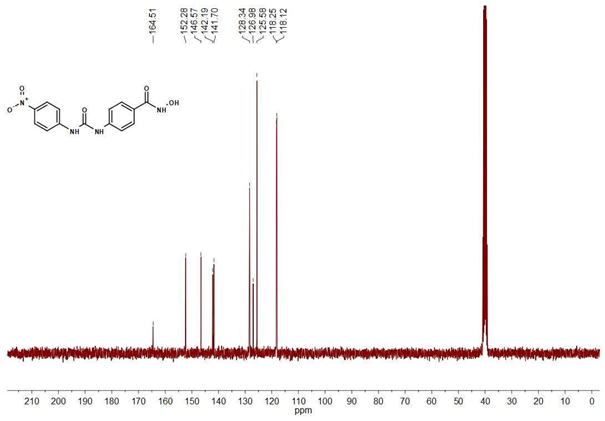


## *N-*hydroxy-4-(3-(*p*-tolyl)ureido)benzamide (**8e**)


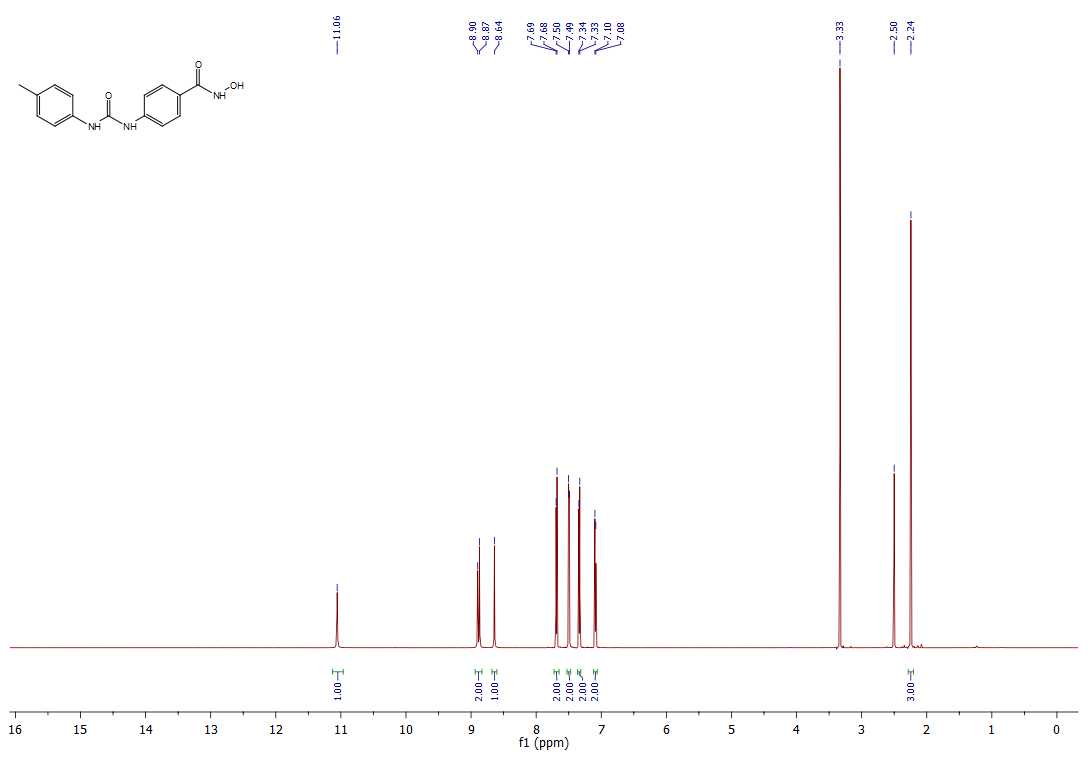


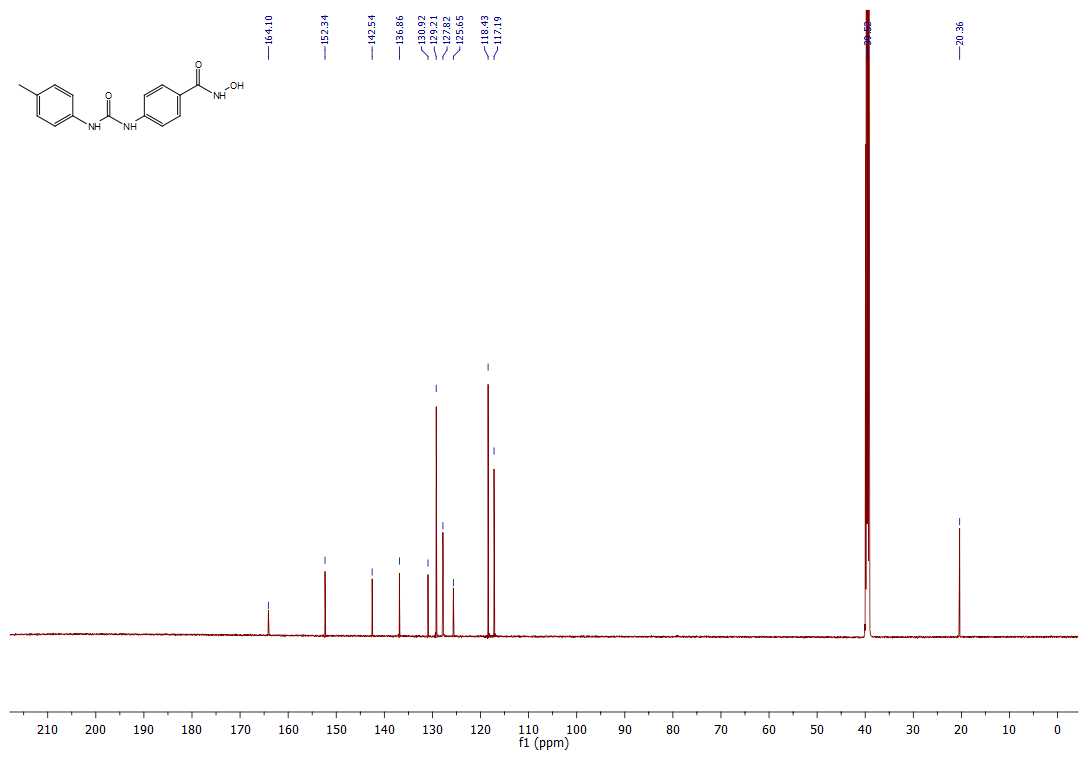


## 4-(3-(4-fluorophenyl)ureido)-*N*-hydroxybenzamide (**8f**)


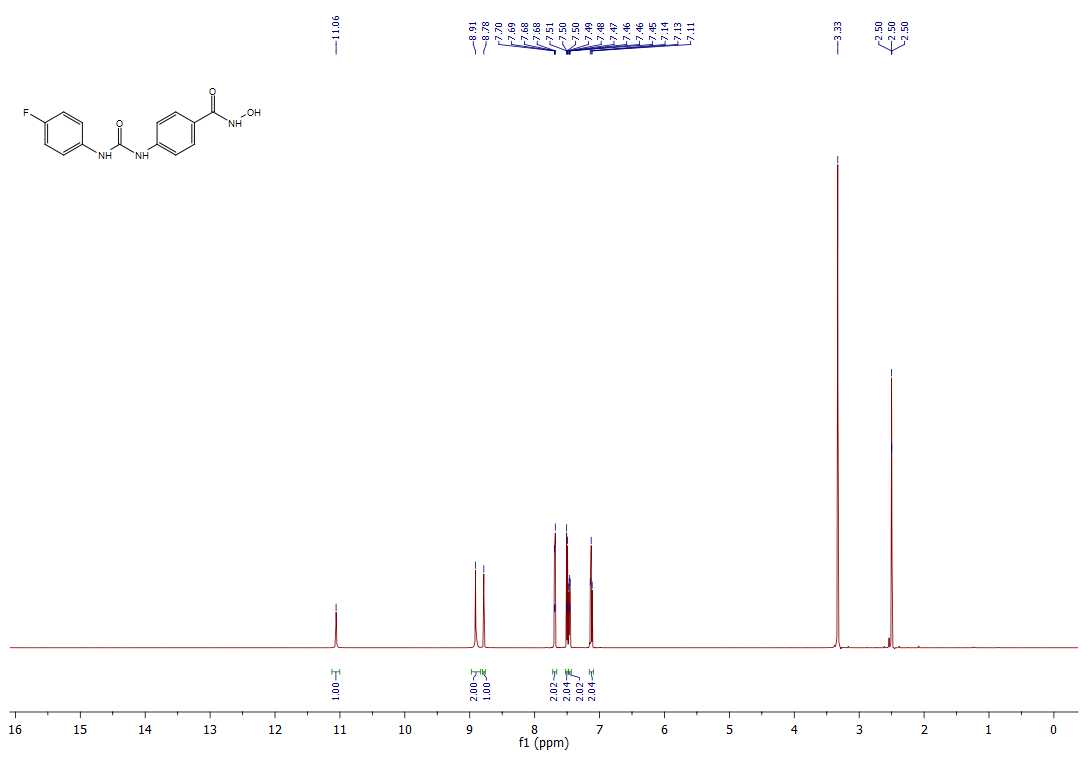


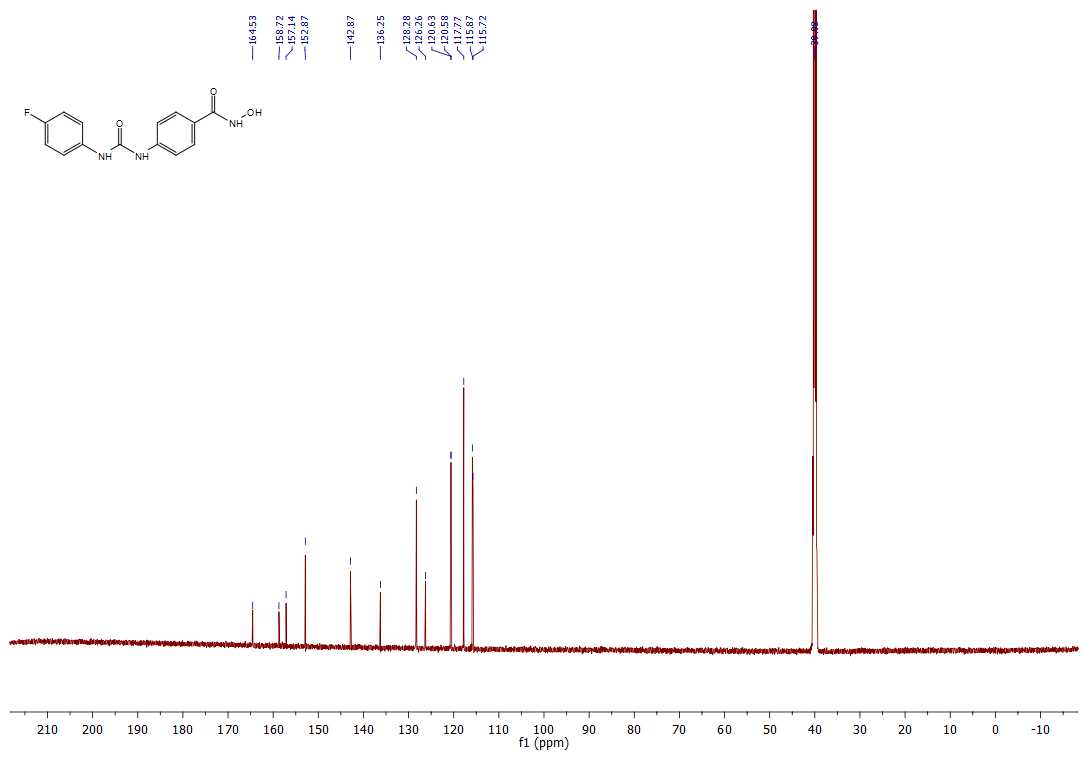


## *N-*hydroxy-4-(3-(4-(trifluoromethoxy)phenyl)ureido)benzamide (**8g**)


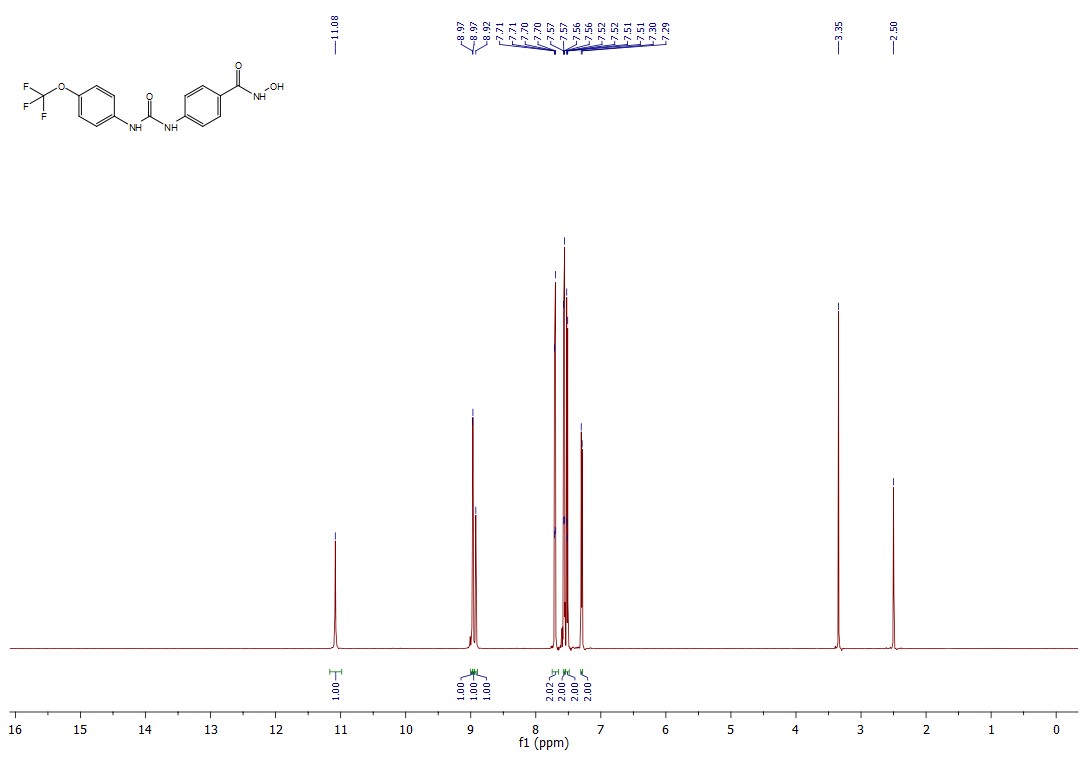


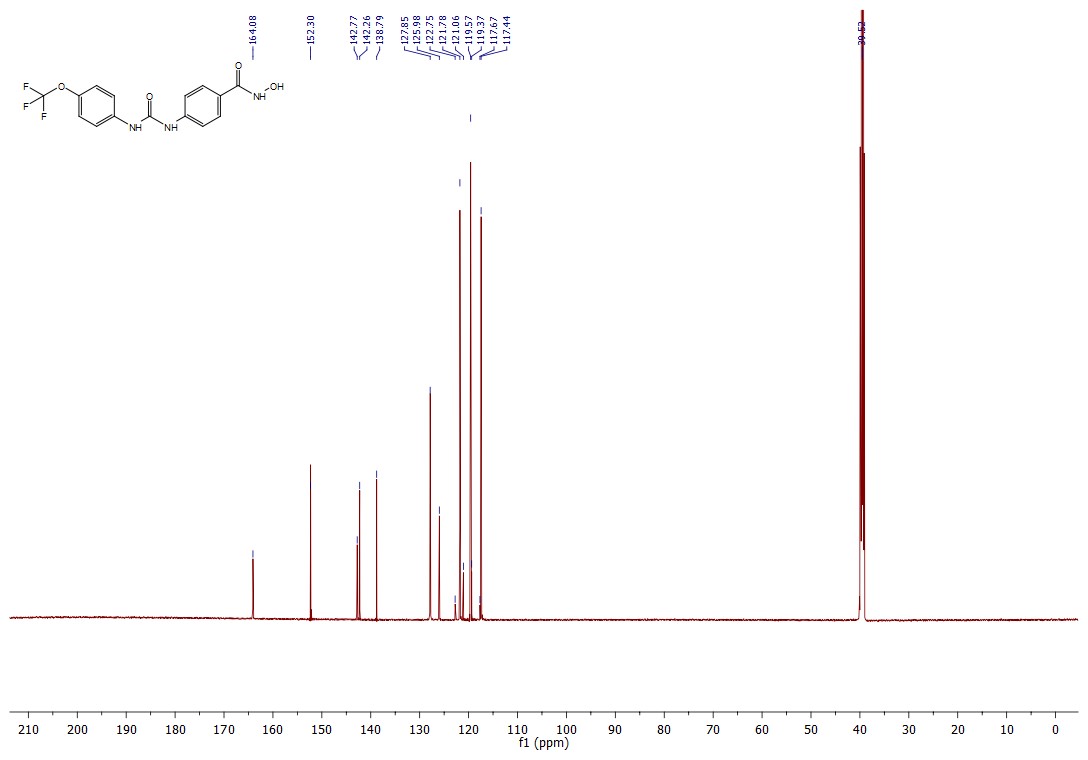


## (*Z*)-*N*-hydroxy-4-(3-(4-(*N'*-hydroxycarbamimidoyl)phenyl)ureido)benzamide (**8h**)


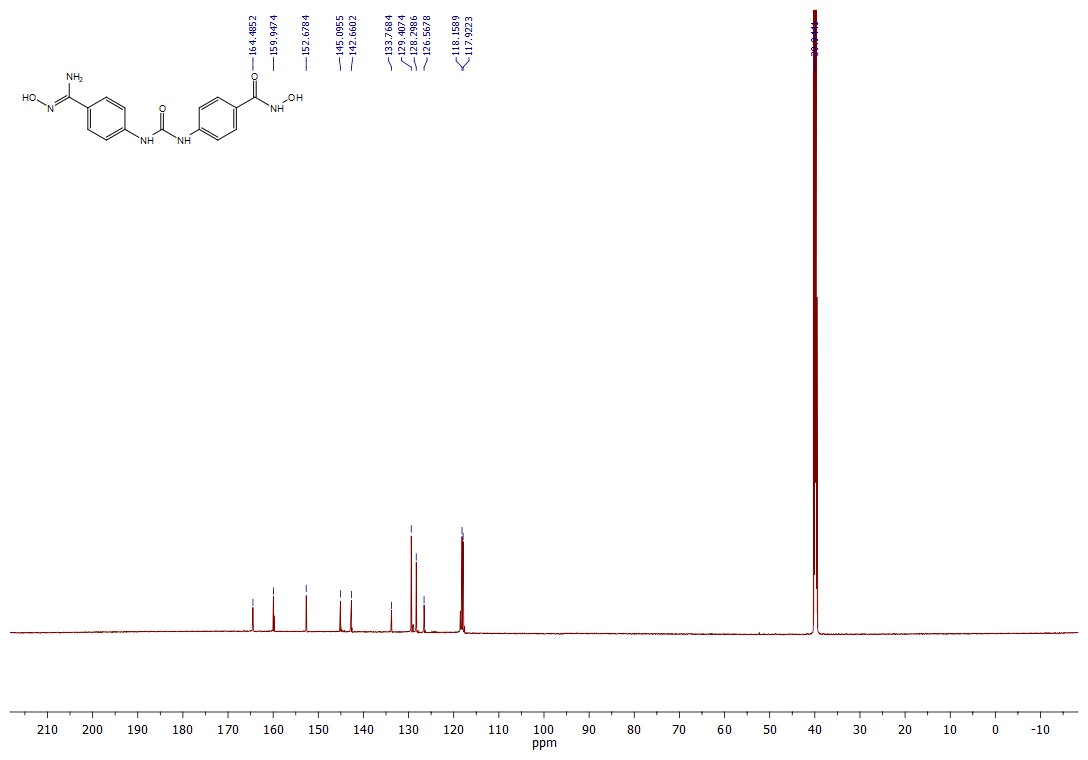

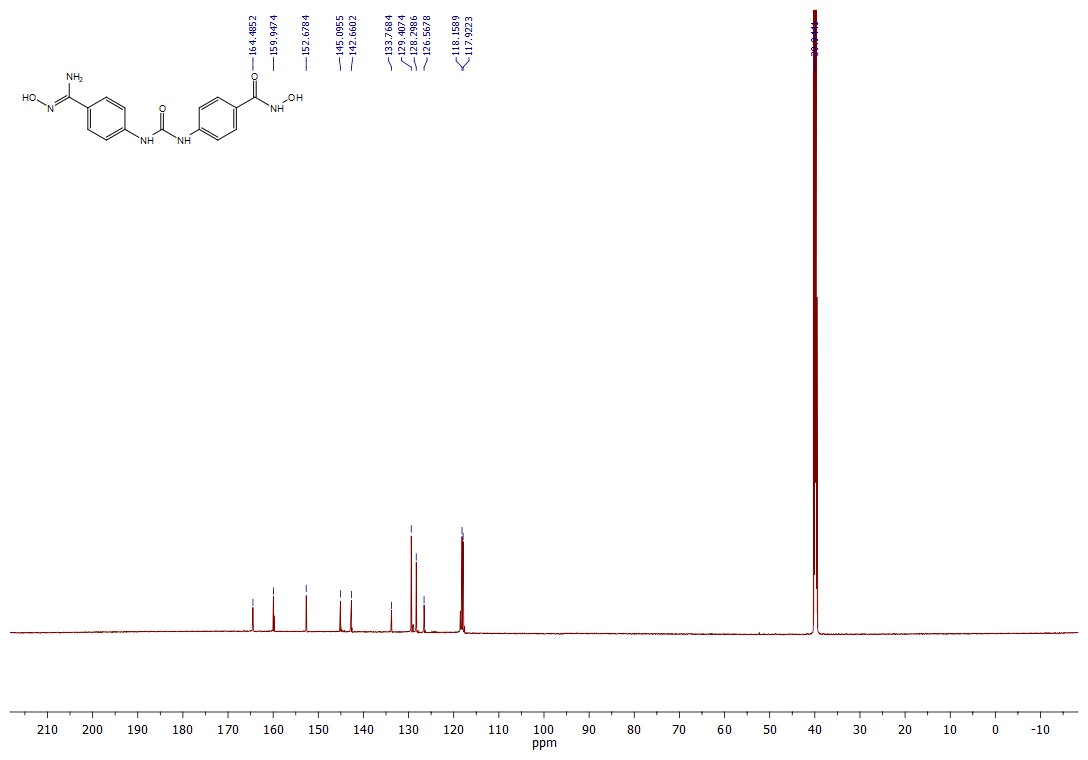


## *N-*hydroxy-4-(3-(4-(trifluoromethyl)phenyl)ureido)benzamide (**8i**)


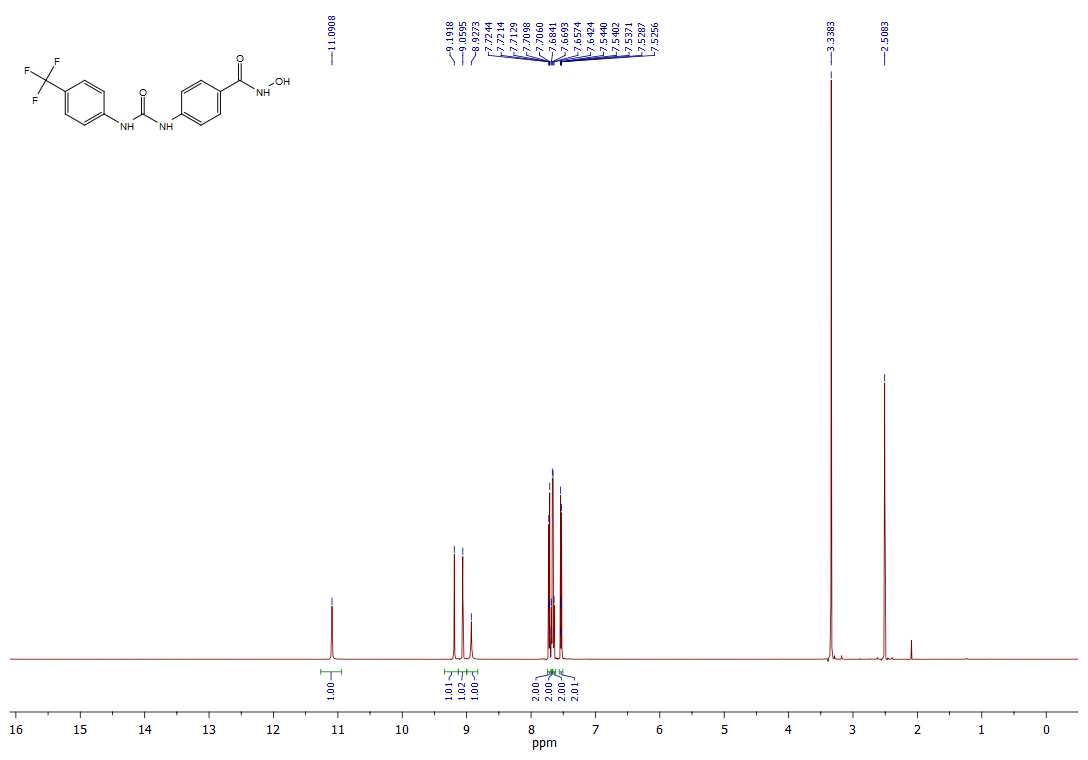

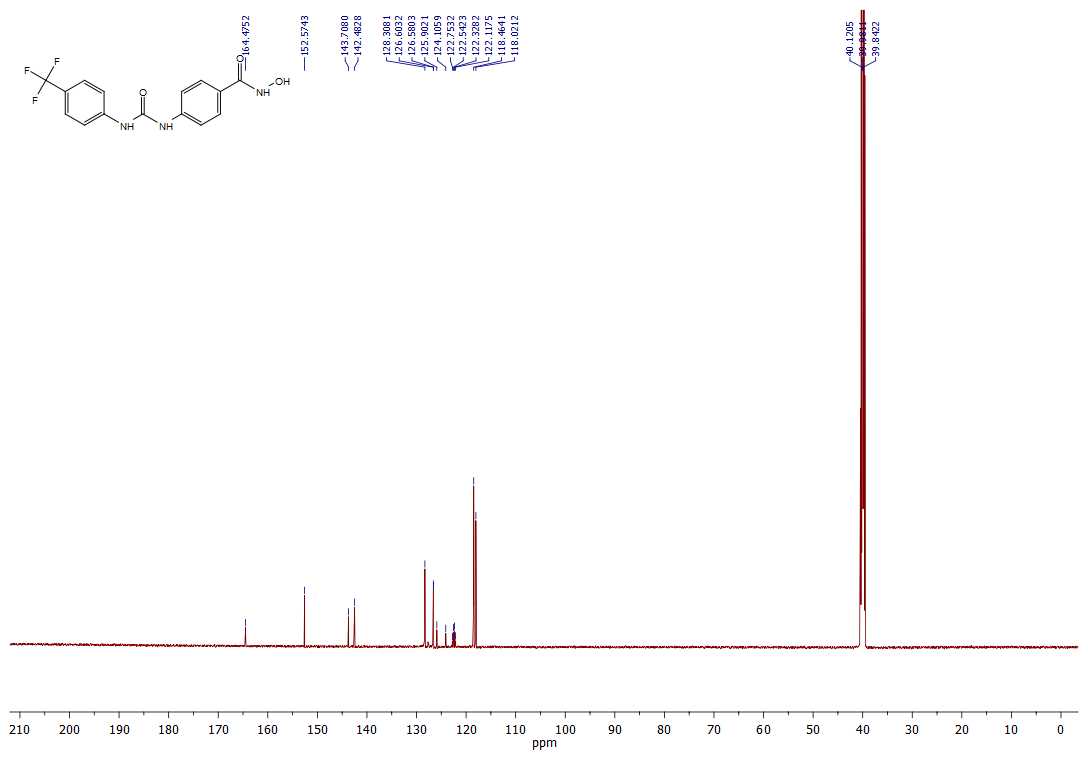


## 4-(3-(4-aminophenyl)ureido)-*N*-hydroxybenzamide (**8j**)


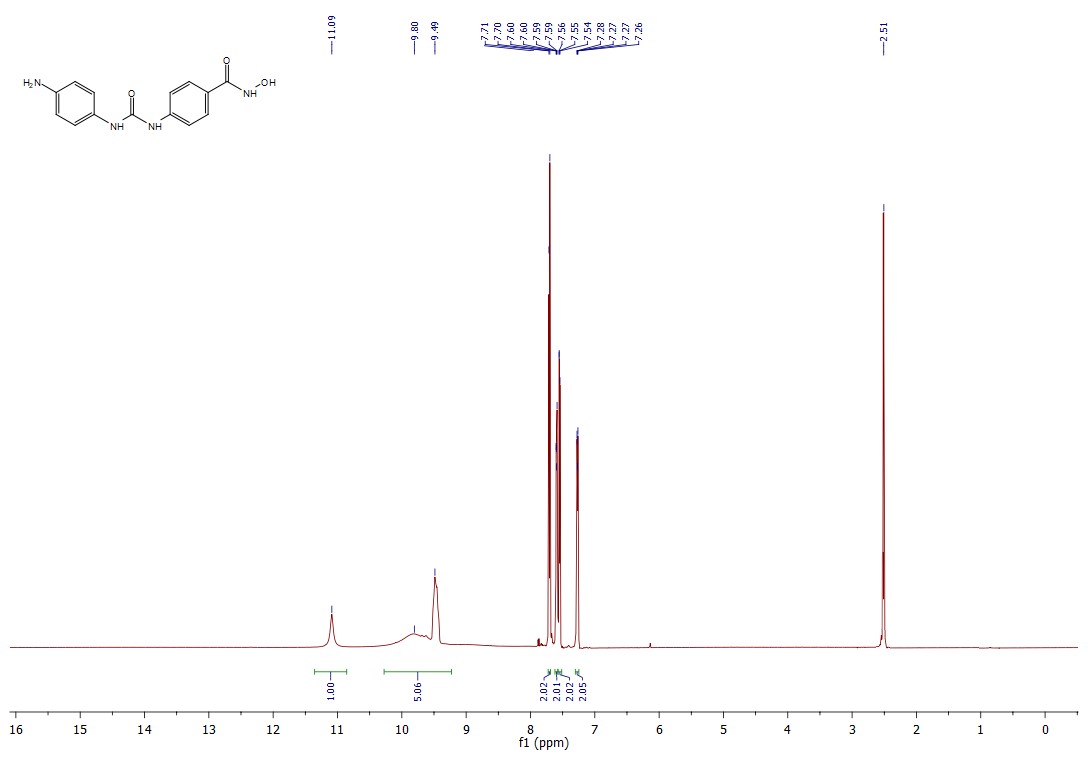


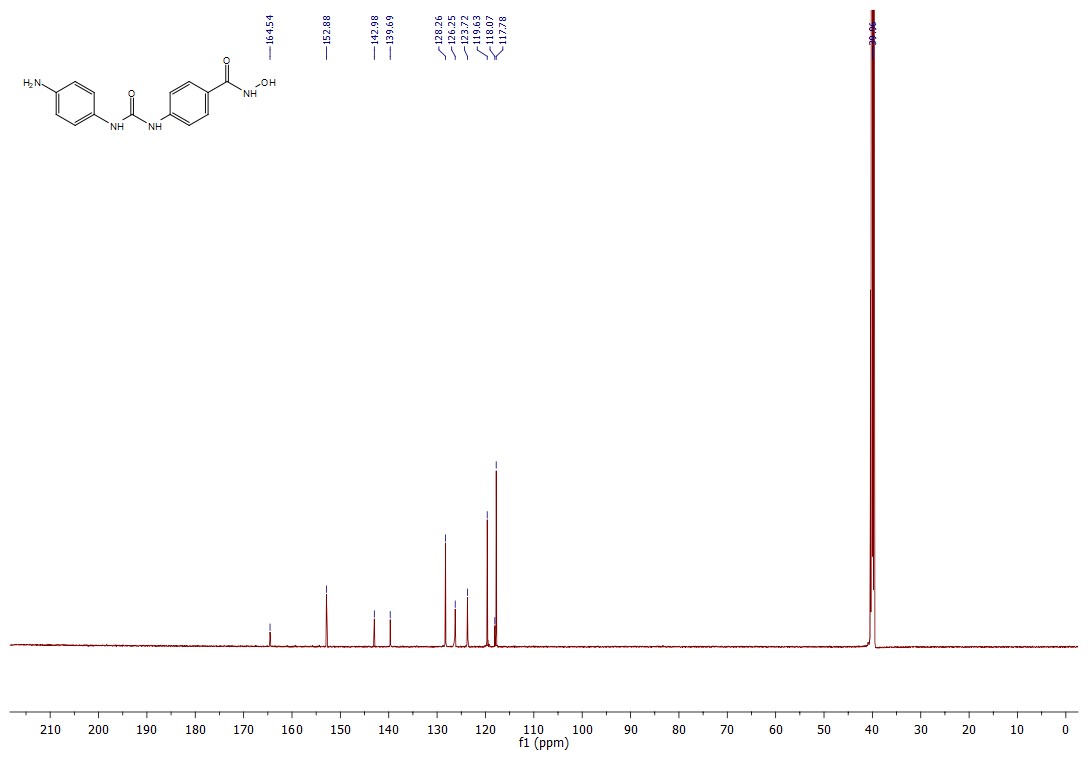


## 4-amino-*N*-hydroxybenzamide (**15**)


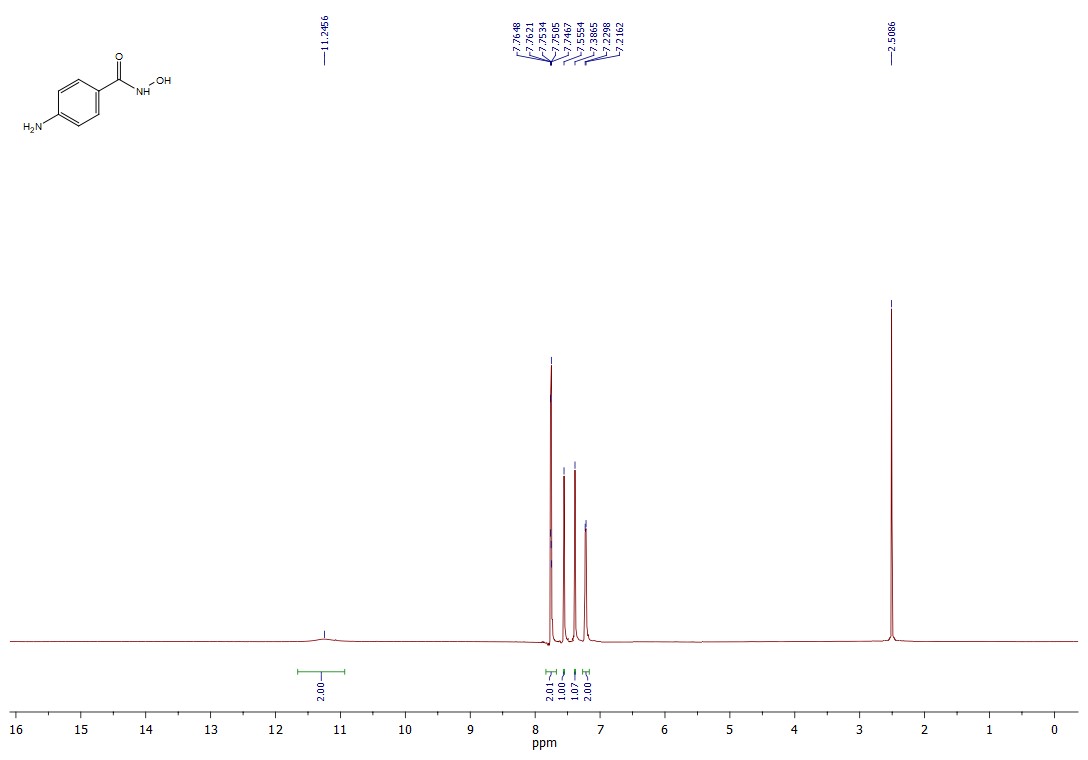

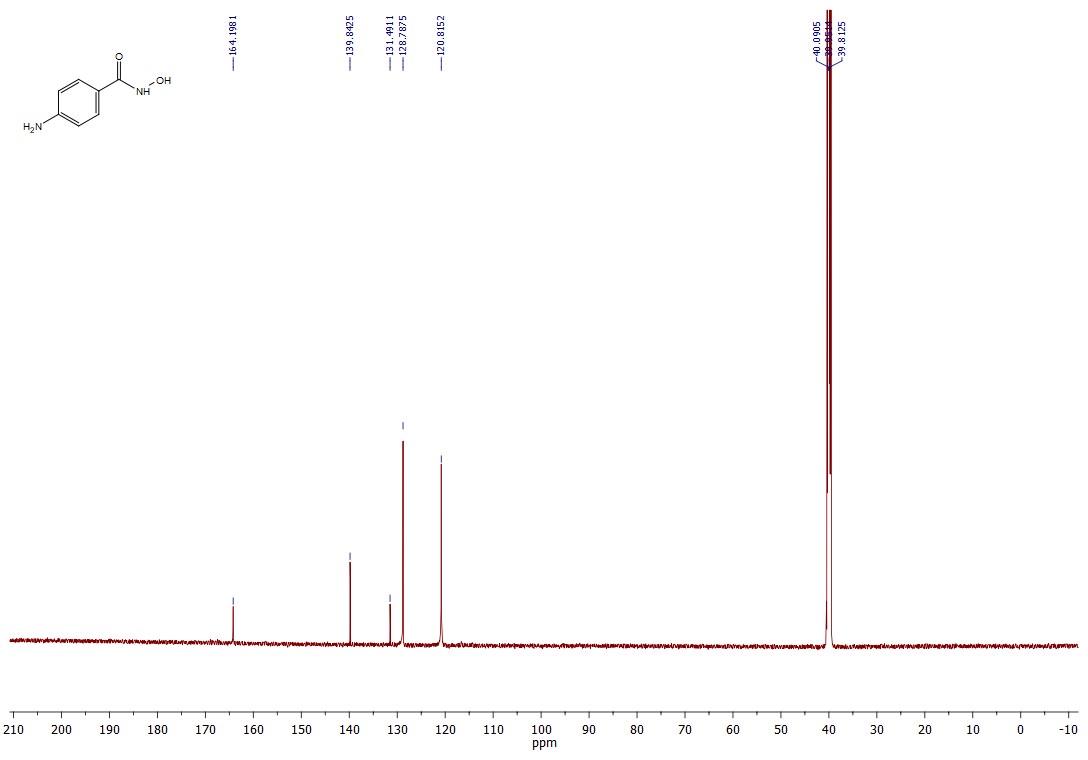


## 4-(3-(4-cyanophenyl)ureido)-*N*-hydroxybenzamide (**8k**)


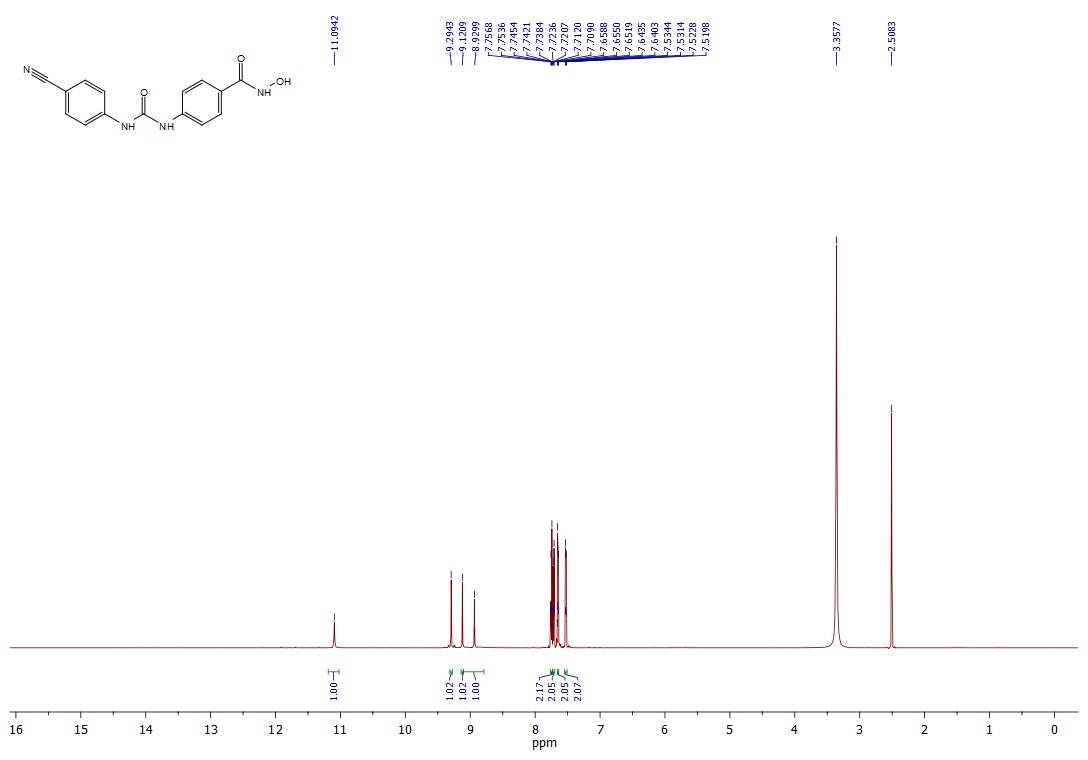


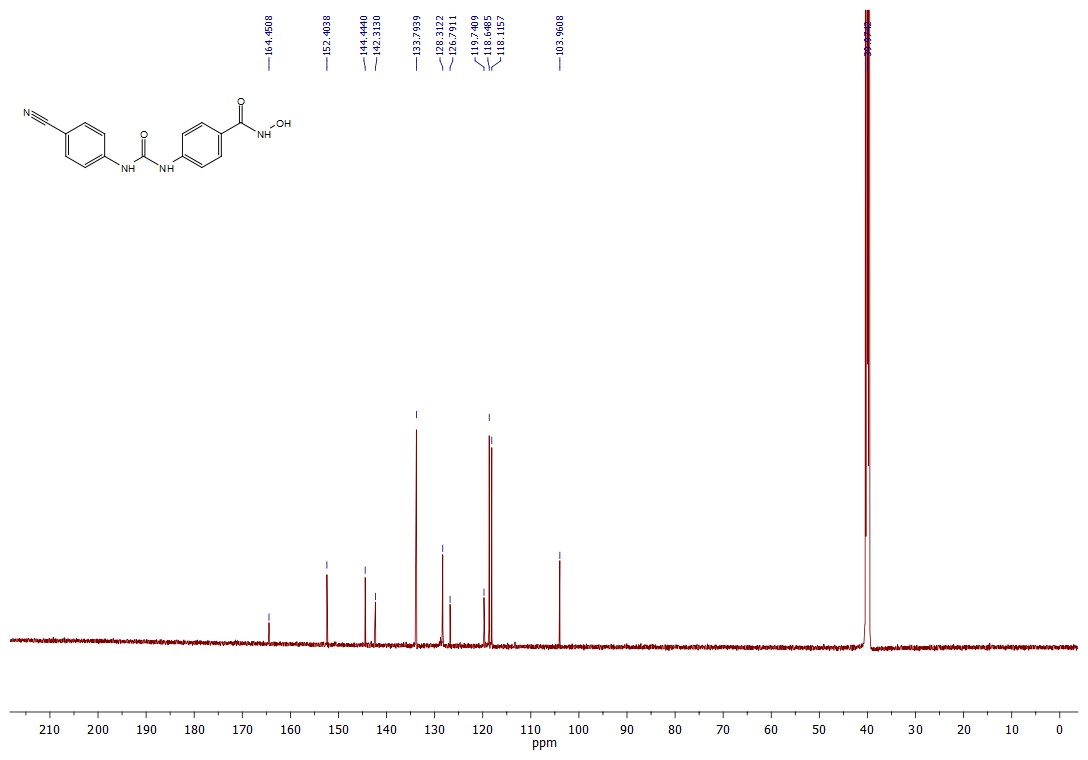


# **Chromatograms**

## (*E*)-*N*-hydroxy-3-(4-(3-phenylureido)phenyl)acrylamide (**6a**)


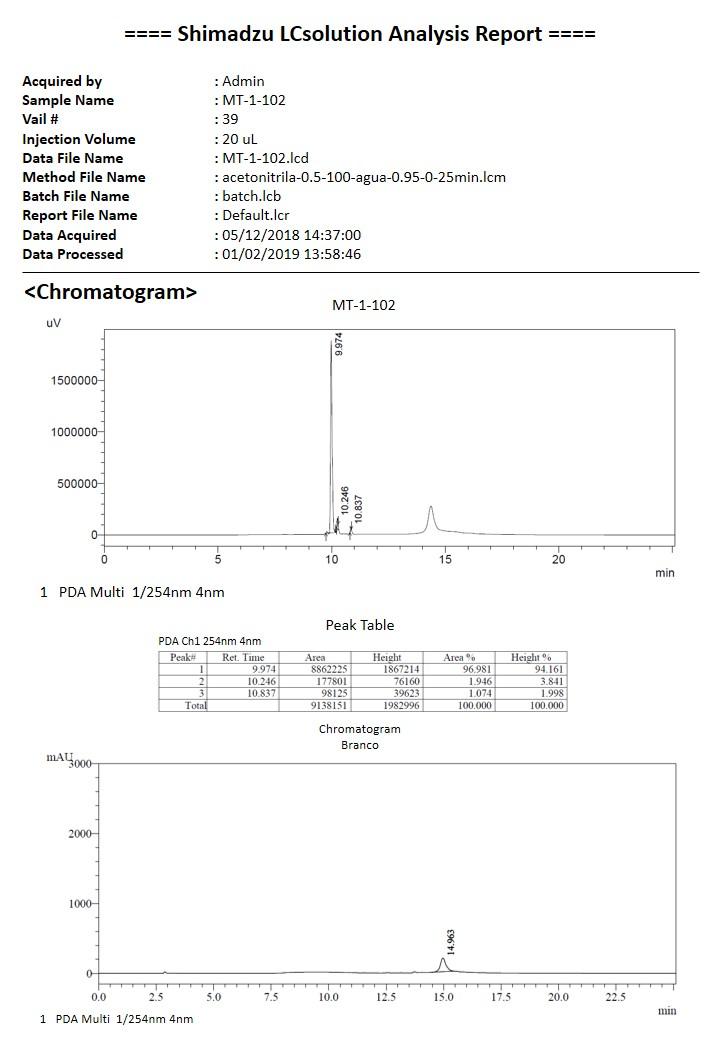


## (*E*)-3-(4-(3-(4-chlorophenyl)ureido)phenyl)-*N*-hydroxyacrylamide (**6b**)


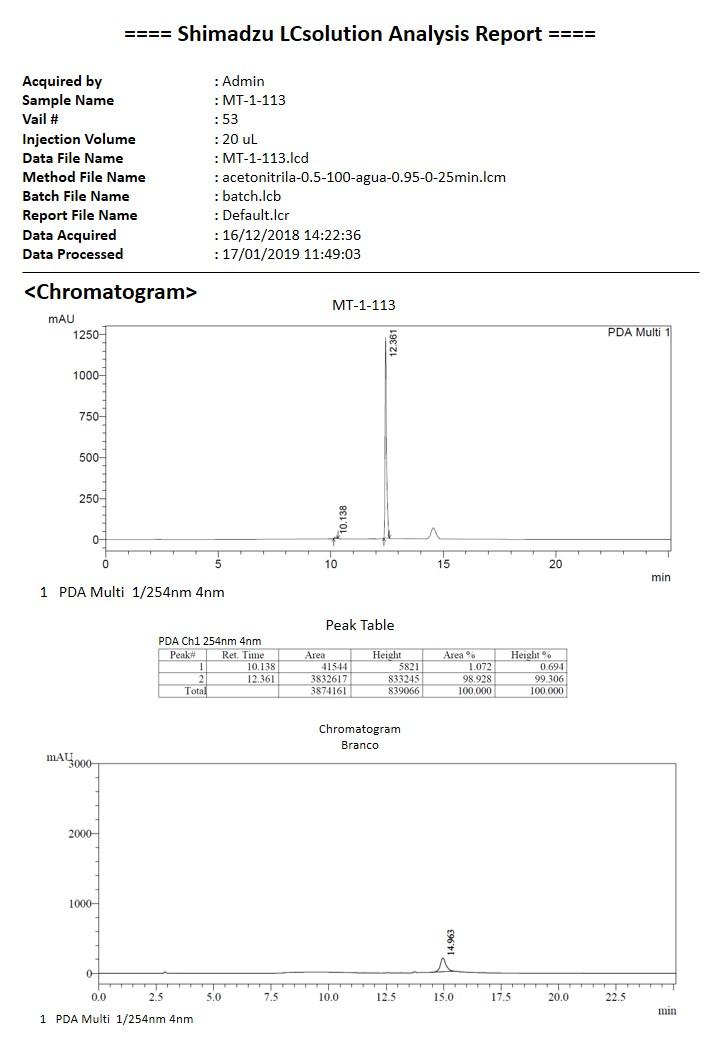


## (*E*)-*N*-hydroxy-3-(4-(3-(4-methoxyphenyl)ureido)phenyl)acrylamide (**6c**)


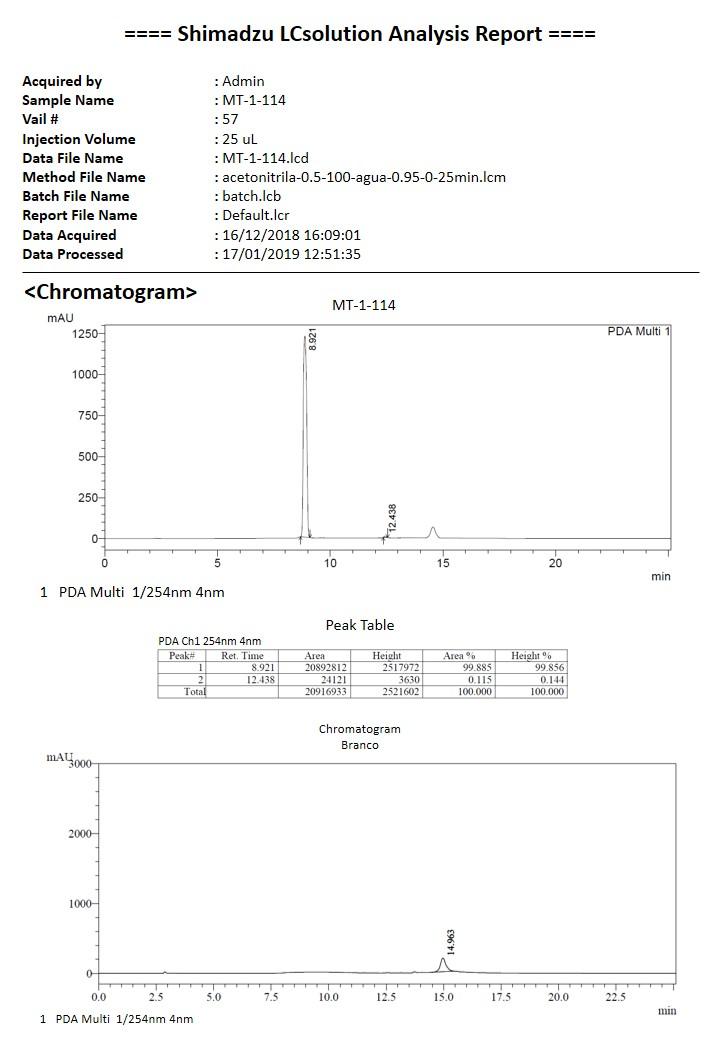


## (*E*)-*N*-hydroxy-3-(4-(3-(4-nitrophenyl)ureido)phenyl)acrylamide (**6d**)


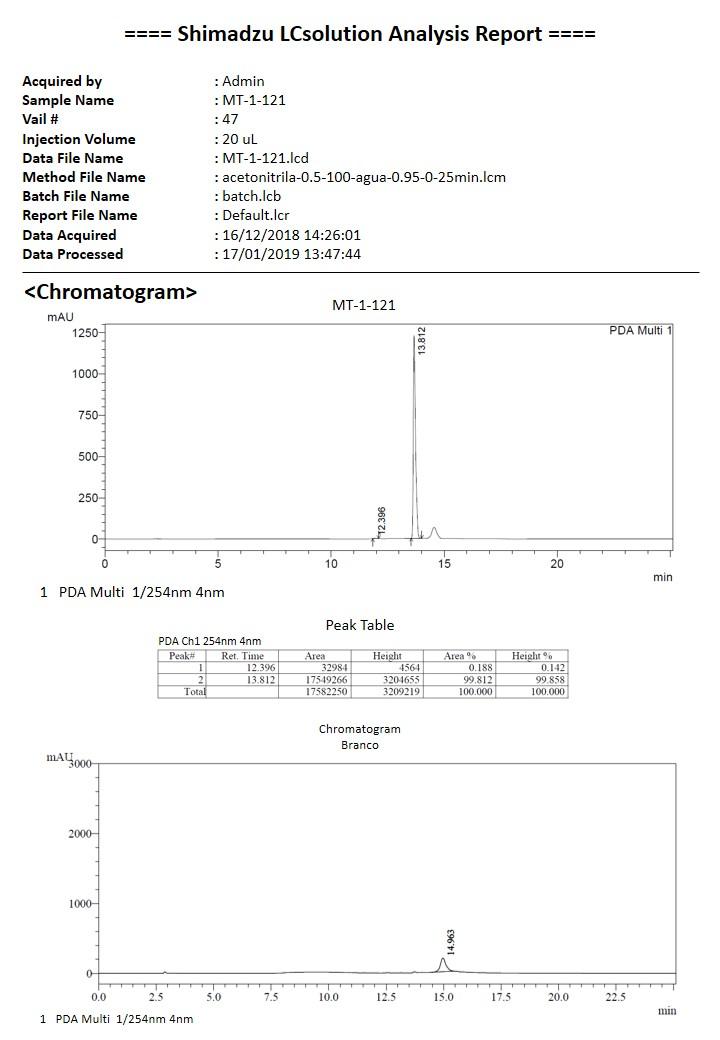


## *N*-hydroxy-3-(4-(3-phenylureido)phenyl)propanamide (**7a**)


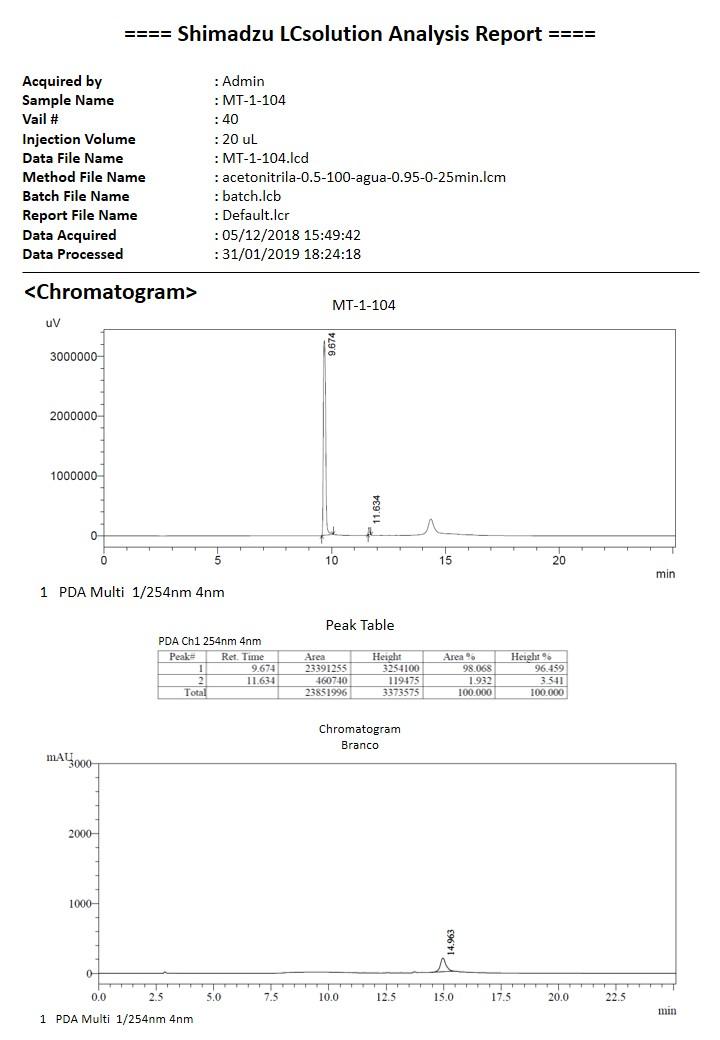


## 3-(4-(3-(4-chlorophenyl)ureido)phenyl)-*N*-hydroxypropanamide (**7b**)


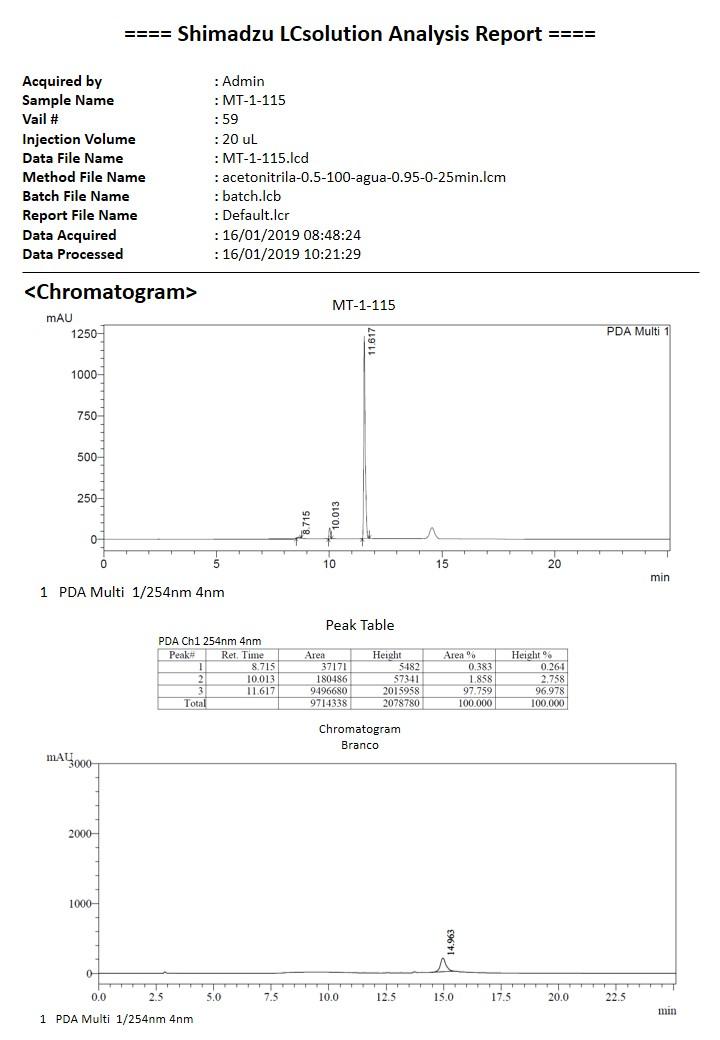


## *N*-hydroxy-3-(4-(3-(4-methoxyphenyl)ureido)phenyl)propanamide (**7c**)


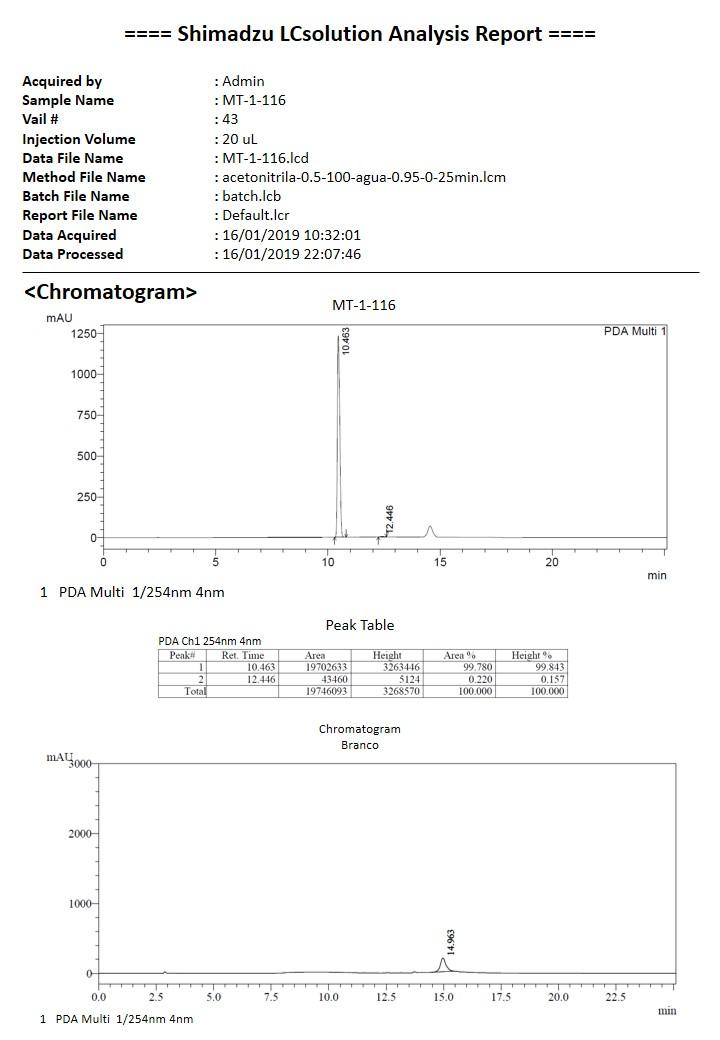


## *N*-hydroxy-4-(3-phenylureido)bezamide (**8a**)


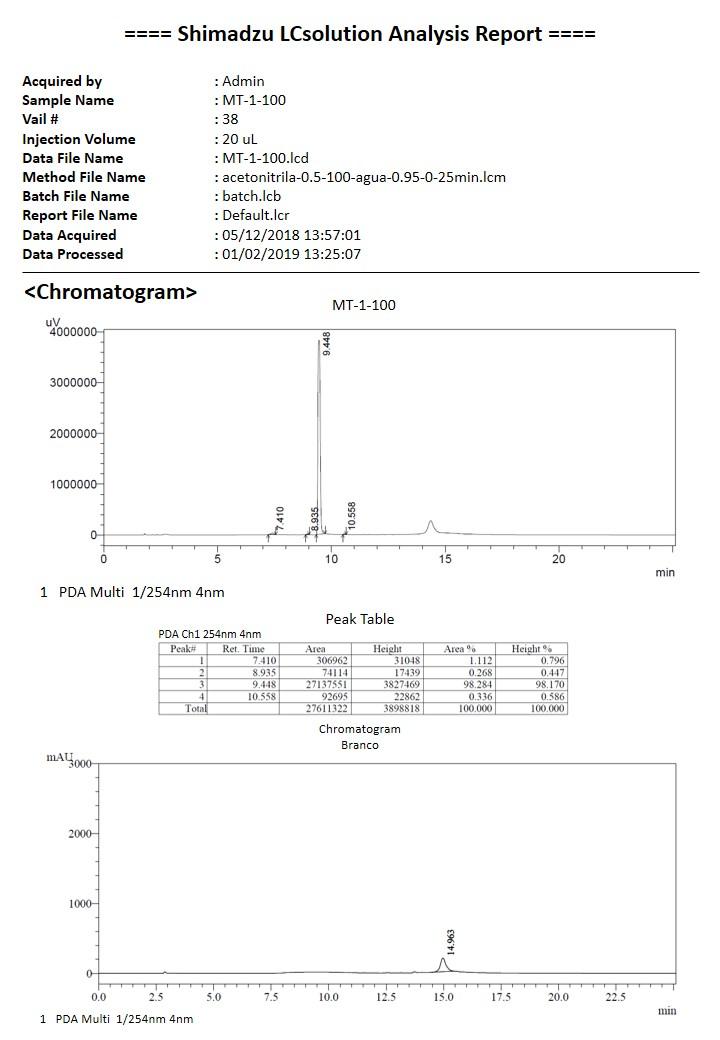


## 4-(3-(4-chlorophenyl)ureido)-*N*-hydroxybenzamide (**8b**)


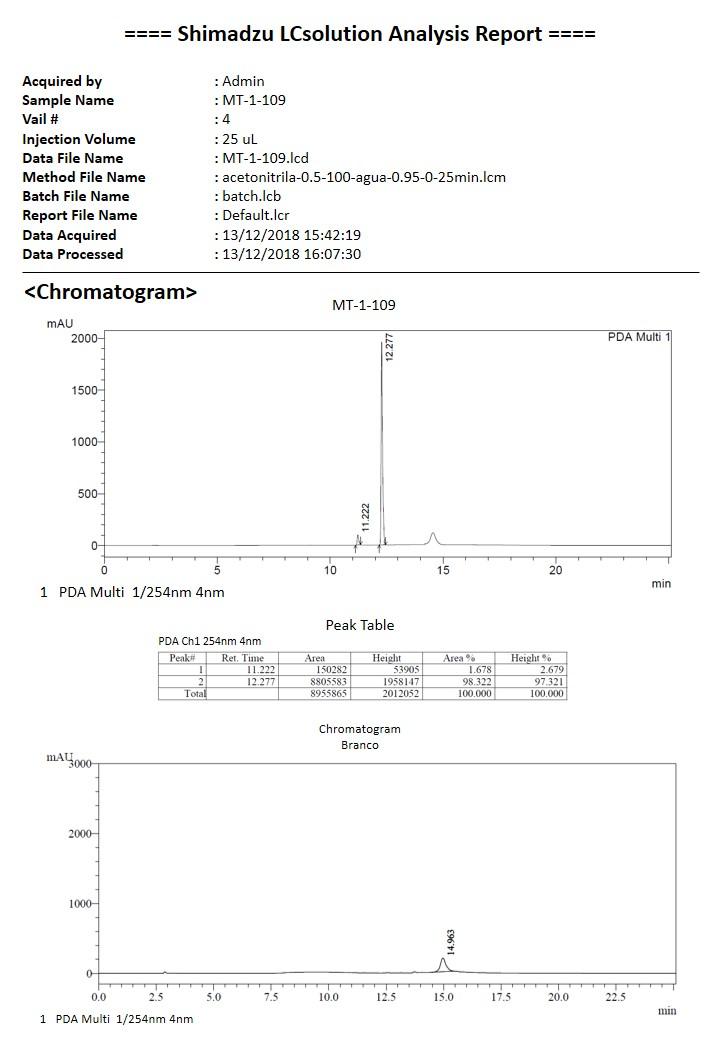


## *N*-hydroxi-4-(3-4(-methoxyphenyl)ureido)benzamide (**8c**)


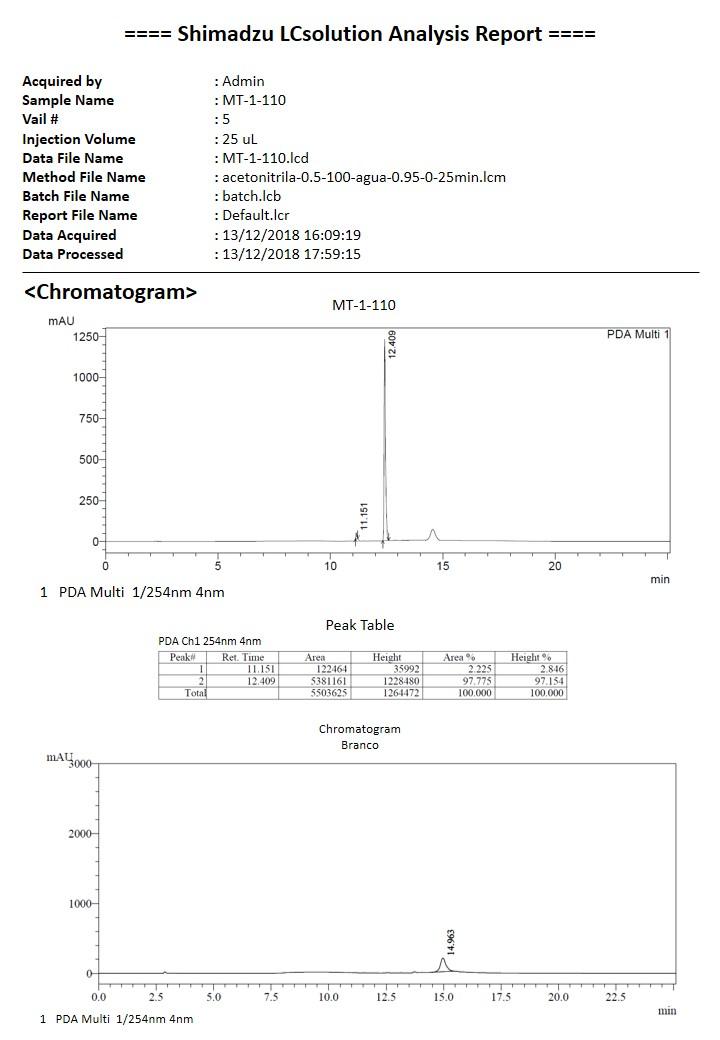


## *N-*hydroxy-4-(3-(4-nitrophenyl)ureido)benzamide (**8d**)


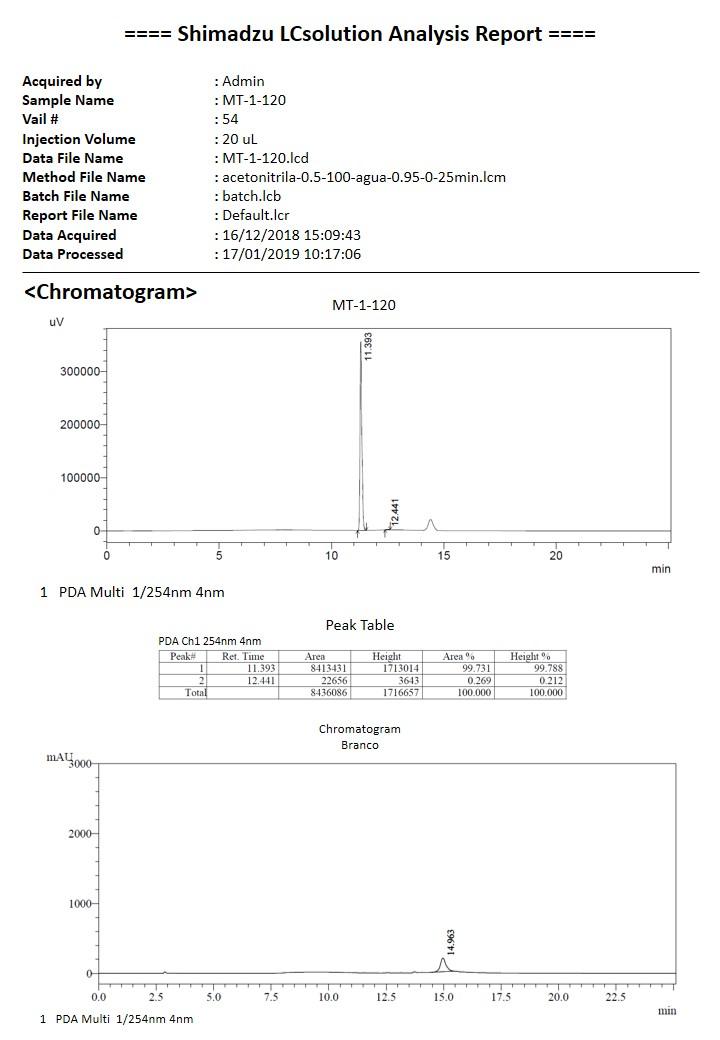


1. **Mass spectra**

## (*E*)-*N*-hydroxy-3-(4-(3-phenylureido)phenyl)acrylamide (**6a**)

## (*E*)-3-(4-(3-(4-chlorophenyl)ureido)phenyl)-*N*-hydroxyacrylamide (**6b**)

## (*E*)-*N*-hydroxi-3-(4-(3-(4-methoxyphenyl)ureido)phenyl)acrylamide (**6c**)

## (*E*)-*N*-hydroxy-3-(4-(3-(4-nitrophenyl)ureido)phenyl)acrylamide (**6d**)

## *N*-hydroxy-3-(4-(3-phenylureido)phenyl)propanamide (**7a**)

## 3-(4-(3-(4-chlorophenyl)ureido)phenyl)-*N*-hydroxypropanamide (**7b**)

## 3 *N*-hydroxy-3-(4-(3-(4-methoxyphenyl)ureido)phenyl)propanamide (**7c**)

## *N*-hydroxi-4-(3-phenylureido)bezamide (**8a**)

## 4-(3-(4-chlorophenyl)ureido)-*N*-hydroxybenzamide (**8b**)

## *N*-hydroxy-4-(3-4(-methoxyphenyl)ureido)benzamide (**8c**)

## *N*-hydroxy-4-(3-(4-nitrophenyl)ureido)benzamide (**8d**)
